# Amphilectene Diterpene Isonitriles and Formamido Derivatives from the Hainan Nudibranch *Phyllidia Coelestis*

**DOI:** 10.3390/md17110603

**Published:** 2019-10-24

**Authors:** Marianna Carbone, Maria Letizia Ciavatta, Emiliano Manzo, Xiao-Lu Li, Ernesto Mollo, I Wayan Mudianta, Yue-Wei Guo, Margherita Gavagnin

**Affiliations:** 1 Consiglio Nazionale delle Ricerche (CNR), Istituto di Chimica Biomolecolare (ICB), Via Campi Flegrei, 34, 80078 Pozzuoli (Na), Italyemanzo@icb.cnr.it (E.M.); emollo@icb.cnr.it (E.M.); 2 State Key Laboratory of Drug Research, Shanghai Institute of Materia Medica (SIMM), Chinese Academy of Sciences, Zuchongzhi Road 555 Zhangjiang Hi-Tech Park, Shanghai 201203, China; lululi666666@126.com; 3 Study Program of Chemical Analysis, Universitas Pendidikan Ganesha, Bali 81116, Indonesia; mudianta@undiksha.ac.id

**Keywords:** nudibranch mollusks, isonitrile diterpenes, stereochemistry, ecological activity

## Abstract

Terpene content of two distinct collections of the nudibranch *Phyllidia coelestis* from the South China Sea has been chemically analyzed. A series of amphilectene diterpenes, most likely of dietary origin, with isocyano and formamido functionalities have been isolated from both collections and spectroscopically characterized by an exhaustive nuclear magnetic resonance (NMR) analysis. Interestingly, the structural architecture of compounds **5**–**7** and **9** with both 8,13-*cis* and 12,13-*cis* ring junctions is unprecedented in the amphilectene skeleton. Metabolite **3**, which was the most abundant in the nudibranch’s mantle**,** has been shown to deter feeding by a generalist predator, supporting its involvement in chemical defense.

## 1. Introduction

Nudibranchs are a group of gastropod mollusks lacking shells [1] that are widely distributed in all seas through the world. Having lost the physical protection of a shell, nudibranchs have developed alternative defense strategies for their survival that include the use of chemicals, which are acquired by the diet or in very few cases de novo biosynthesized [2,3]. Nudibranchs colonized many different ecological niches in benthic communities because of feeding on a variety of marine organisms comprising sponges, cnidarians, tunicates and bryozoans. Due to this, the chemistry of nudibranchs is characterized by a very high diversity being mostly related to that of the preys, typically some of the most prolific producers of natural products. Most of these dietary compounds display toxicity or feeding deterrence and thus they are assumed to be involved in the defensive mechanisms of the mollusks [4,5,6]. Phyllidiids are a group of nudibranchs well studied from a chemical point of view since the first report in the 1970s, when Scheuer and co-workers identified a unique toxic sesquiterpene with an isocyano group, 9-isocyanopupukeanane, from the defensive mucous secretion of Hawaiian *Phyllidia varicosa* [7]. The compound was also found in a sponge of the *Hymeniacidon* genus on which the mollusk was feeding [7]. Following this, a number of phyllidiid species belonging to the genera *Phyllidia* and *Phyllidiella* have been chemically investigated resulting in the isolation of numerous bioactive sesquiterpenes with different structural architectures all exhibiting nitrogenous functional groups, such as cyano, isocyano, isothiocyano, and formamido functionalities [8,9]. These compounds display pronounced antimalarial [10,11], antifouling [12] and other biological properties [9,13,14]. They are characteristic metabolites of sponges of various genera, mainly *Acanthella*, *Axinella*, and *Axynissa*, that are included in the diet of phyllidiid mollusks.

**Figure 1:**
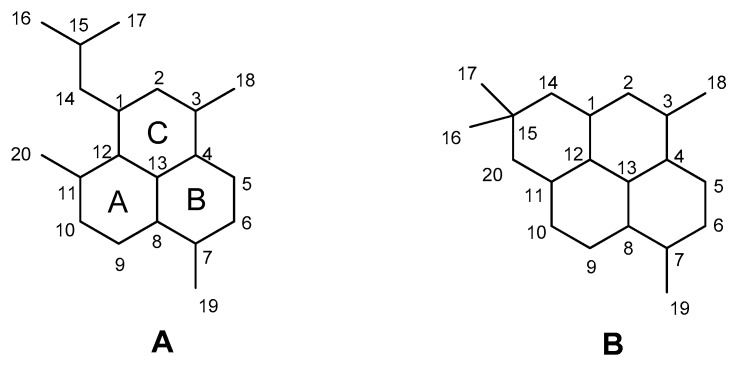
Amphilectane (**A**) and cycloamphilectane (**B**) skeletons.

In continuing our chemical studies on mollusks from South China Sea [15,16,17,18,19], we have investigated the chemistry of the nudibranch *Phyllidia coelestis.* By contrast with our previous report on this species [20], the secondary metabolite pattern of the collections now examined was found to be characterized by the presence of a series of diterpene isonitriles and formamido derivatives with the amphilectane and cycloamphilectane skeletons [21] (Figure 1). The isolation of diterpenes **1**–**10** and the structural determination of new amphilectadienes **5**–**9** exhibiting the uncommon 8,13-*cis* and the unprecedented 12,13-*cis* ring junctions are presented here.

## 2. Results

**Figure 2:**
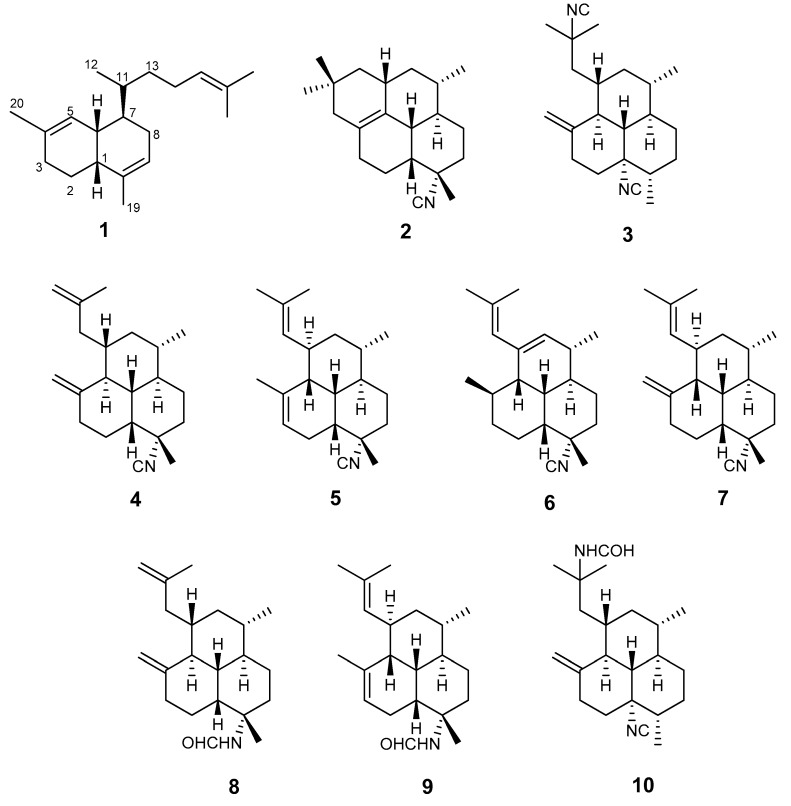
Structures **1**–**10**.

Two populations of *Phyllidia coelestis* of 10 and 9 individuals were collected at Hainan Island (South China Sea) during January 2002 and 2013, respectively. The mollusks were dissected into mantle and internal organs that were separately extracted (see Materials and Methods). The ether extracts of dissected parts, mantle and internal organs, of both *P. coelestis* collections were analyzed by thin layer chromatography (TLC). Even though the two mollusk populations were sampled in very distant periods they showed surprisingly a comparable secondary metabolite patterns. Lipophilic components of the diethyl ether extracts were observed to occur in a similar manner in mantle and internal organs in both collections but with a different relative distribution. In particular, a series of metabolites at *R*_f_ 0.22–0.90 (petroleum ether/diethyl ether, 9:1) and at *R*_f_ 0.20 (petroleum ether/diethyl ether, 2:8) were found to be significantly concentrated in both mantle extracts. All the extracts were separately cromatographed (see Materials and Methods) and the fractions enriched with compounds of interest were preliminarily analyzed by ^1^H nuclear magnetic resonance (NMR) spectroscopy revealing the presence of several cyclic diterpenes with isocyano and formamido functionalities. After subsequent purification steps, a total of 10 diterpenes **1**–**10** (Figure 2) were obtained including five unreported amphilectadiene compounds **5**–**9**. In particular, (1R*,6S*,7R*,11R*)-biflora-4,9,15-triene (**1**) [22,23,24], (1S*,3S*,4R*,7S*,8R*,13R*)-7-isocyano-11-cycloamphilectene (**2**) [25], (1*S*,3*S*,4*R*,7*S*,8*S*,12*S*,13*S*)-8,15-diisocyano-11(20)-amphilectene (**3**) [21,23,24], (1S*,3S*,4R*,7S*,8R*,12S*,13S*)-7-isocyanoamphilecta-11(20),15-diene (**4**) [23,24], and new (1R*,3S*,4R*,7S*,8R*,12R*, 13S*)-7-isocyanoamphilecta-10,14-diene (**5**), (3R*,4R*,7S*,8R*,11R*,12S*,13S*)-7-isocyanoamphilecta-1,14-diene (**6**) and (1R*,3S*, 4R*,7S*,8R*,12R*,13S*)-7-isocyanoamphilecta-11(20),14-diene (**7**) were purified from the less polar fractions. Additional three formamido derivatives, new (1S*,3S*,4R*,7S*,8R*,12S*,13S*)-7-formamidoamphilecta-11(20),15-diene (**8**) and (1R*,3S*,4R*,7S*,8R*,12R*,13S*)-7-formamido amphilecta-10,14-diene (**9**), and known (1*S*,3*S*,4*R*,7*S*,8*S*,12*S*,13*S*)-8-isocyano-15-formamido-11(20)-amphilectene (**10**) [21], were obtained from more polar fractions. The main compound was amphilectene **3** (18.0 mg) whereas all the others were isolated as minor metabolites (1.0~4.0 mg for each compound). Known metabolites were identified by comparison of their spectroscopic data (NMR, mass spectrometry (MS), and optical rotation) with the literature [21,22,23,24,25] whereas the structures of new compounds were determined by extensive and detailed spectroscopic studies.

Compound **5** had the molecular formula C_21_H_31_N as it was deduced by the sodiated molecular peak at **m*/*z** 320.2532 [M + Na]^+^ in the high resolution electron spray MS (HRESIMS) spectrum. The ^1^H and ^13^C NMR data (Table 1 and Table 2) revealed the presence of two trisubstituted double bonds and five methyl groups including three vinyl methyls [δ_H_ 1.65 (br s, H_3_-17) and 1.72 (6H, br s, H_3_-16 and H_3_-20); δ_C_ 17.7 (q, C-17), 21.0 (q, C-20) and 25.8 (q, C-16)], a secondary methyl linked to a sp^3^ carbon atom [δ_H_ 0.82 (d, *J* = 6 Hz, H_3_-18); δ_C_ 19.6 (q, C-18)], and a methyl linked to a non-protonated carbon bearing the isonitrile group [δ_H_ 1.57 (s, H_3_-19); δ_C_ 26.4 (q, C-19)]. This pattern was consistent with the presence of the amphilectadiene framework, the same as of co-occurring known compound **4**. The inspection of 2D NMR spectra led us to confirm the tricyclic carbon skeleton and locate one of two double bond in the isobutenyl moiety (Δ^14,15^) and the second one at C-10 (Δ^10,11^) in A ring, as depicted in structure **5**. Once assessed the gross structure, a series of NOESY and nuclear Overhauser effect (NOE) difference experiments were recorded with the aim at defining the relative configuration of seven stereogenic centers of **5**. Unfortunately, the analysis of the spectra was very difficult and the observed effects were not diagnostic due to the presence of several overlapping signals that hampered unambiguous assignment. It should be considered that the stereochemical determination of amphilectane-based compounds is really challenging [26] and only X-ray diffraction analysis should allow the correct configuration to be determined [21,24,25,27,28]. However, failing a suitable crystal of **5** for the X-ray study, further insight into stereochemical assessment was provided by comparative analysis of ^13^C NMR values of **5** (Table 2) with data reported for the two amphilectene series that have been described to date [9], i.e., those exhibiting the.

**Figure 3:**
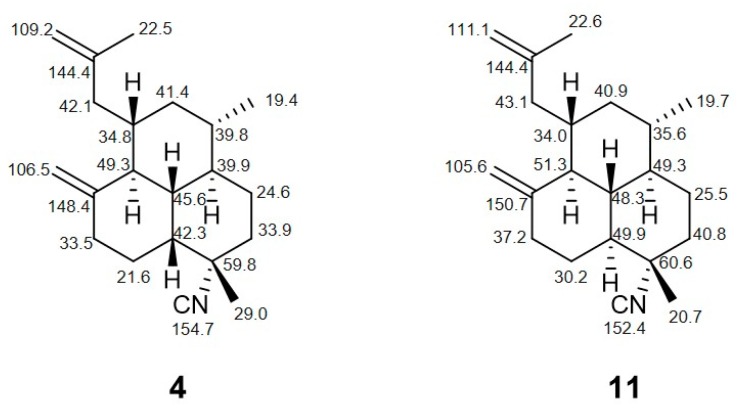
^13^C NMR assignment of 7-isocyanoamphilecta-11(20),15-diene epimers. 8,13-*cis***4** [23,24] and 8,13-*trans* **11 [27,28]**.

8,13 *cis*-ring junction and those with all *trans*-ring junctions. In particular, the carbon assignment for two selected model compounds **4** [23,24] and **11 [27,28]**, the structures of which have been secured by X-ray crystallographic studies, was considered (Figure 3). Expected distinct patterns for the carbon values of A and B rings of the amphilectane skeleton were observed for **4** and **11**. In *trans*-epimer **11**, the chemical shifts of C-4, C-6, C-8, and C-9 were deshielded (about 7~10 ppm) whereas C-19 methyl resonated at lower ppm (about 8 ppm) with respect to *cis*-epimer **4** (Figure 3), consistent with different β-effects in the two junction arrangements. This chemical shift pattern was observed for almost all *trans*- and *cis*-amphilectenes described in the literature [9], and C-6 and C-19 chemical shift values appeared to be almost diagnostic.

Interestingly, the NMR carbon pattern of compound **5** (Table 2) revealed it to be almost different from both known series suggesting a different stereochemical arrangement involving junction stereogenic centers. The ^13^C NMR resonances of C-6 and C-19 observed for compound **5** (Table 2) were comparable with those of amphilectenes of 8,13-*cis* series [23,24,26] suggesting the A/B *cis*-stereochemistry. Unfortunately, junction protons H-8 (δ_H_ 1.82) and H-13 (δ_H_ 1.70) (Table 1) resonating in a crowded region of the spectrum were overlapped with other signals and thus *J*_H-H_ coupling constant analysis could not be carried out. However, having tentatively assessed the A/B junction, the analysis of the ^1^H NMR multiplicity of the H-12 (δ_H_ 2.01) signal was made by ^1^H-^1^H decoupling experiments with the aim at establishing the stereochemistry of 12,13-ring junction. The irradiation of the ^1^H signal at δ_H_ 2.93 (H-1) simplified the H-12 multiplet to a broad singlet, implying that H-12 had to be equatorial and, consequently, indicating a *cis*-relationship between H-12 and H-13. This relative configuration was consistent with the observed chemical shifts of C-2 (δ_C_ 35.5) and C-4 (δ_C_ 37.5) that were shielded with respect to literature amphilectene models with the 12,13-*trans* junction, due to the axial substituent in β-position. The relative configuration of C-1 was defined by examining the multiplicity of H-1 signal (m, *w*_1/2_ = 17 Hz). The ^1^H-^1^H decoupling of H-14 vicinal proton at δ_H_ 5.46 modified H-1 signal as a sharp multiplet indicating the H-1 residual couplings with H_2_-2 and H-12 to be small according to an equatorial orientation. This stereochemical assessment was further supported by δ_C_ values of C-3 and C-13 resonating at lower ppm due to the β-effects of the axial isobutenyl chain at C-1. The stereogenic centers C-3, C-4 and C-7 were supposed to have the established amphilectene configuration. Thus, the structure of compound **5** exhibiting previously unreported 8,13- and 12,13-*cis* junctions was tentatively proposed as (1R*,3S*,4R*,7S*,8R*,12R*, 13S*)-7-isocyanoamphilecta-10,14-diene.

**Figure 4:**
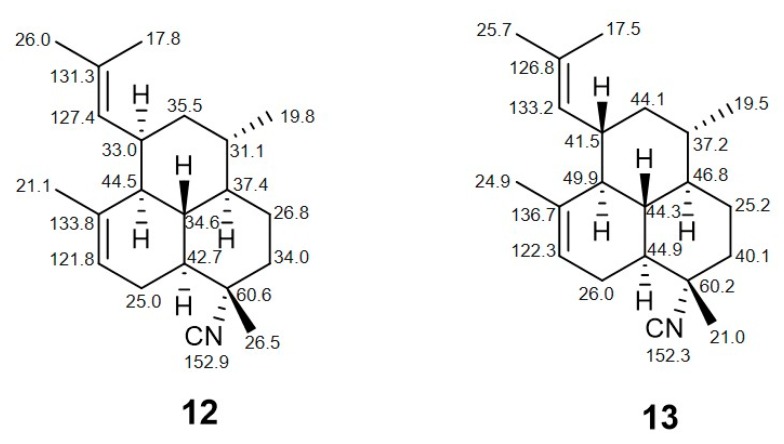
^13^C NMR assignment of 7-isocyanoamphilecta-10,14-diene C-1 epimers **12** [29] and **13** [27].

It should be noted that ^1^H and ^13^C NMR data of **5** were identical with those of previously reported amphilectadiene **12**, a compound isolated from an Okinawan specimen of the sponge *Stylissa* sp. [29] and determined to be the C-1 epimer of (1S*,3S*,4R*,7S*,8S*,12S*,13S*)-7-isocyanoamphilecta-10,14-diene (**13**) [27]. The structure **12** proposed by Mitome et al. [29] is in conflict with our structural hypothesis (structure **5**) and, failing X-ray diffraction studies, the true structure cannot be unequivocally proven. However, the stereochemical arrangement proposed in structure **12** appears to be not consistent with the NMR data (Figure 4). In particular, the differences observed in the carbon values of C-2, C-4, C-6, and C-19 with respect to compound **13** [27] indicate different junction stereochemistry, as has been discussed above. According to this, structure **12** [29] should be incorrect.

On the other side, further evidence corroborating the proposed structure **5** was obtained by extensive analysis of spectroscopic data of co-occurring structurally related compound **9** that revealed to be the corresponding formamido derivative of **5**, as following described.

Compound **9** showed in the ESIMS spectrum the sodiated molecular peak at **m*/*z** 338 [M + Na]^+^ consistent with the molecular formula C_21_H_33_NO. The ^1^H and ^13^C NMR spectra (Table 1 and Table 2) revealed a close similarity with those of **5** strongly suggesting the same amphilectadiene skeleton including the presence of the isobutenyl chain at C-1 and the Δ^10^ double bond. It is interesting to note that the spectra of **9** contained more signals than expected from the molecular formula due to the presence of two isomers (ratio 1:1) with a conformational equilibrium slower than NMR scale time. The ^1^H NMR spectrum evidenced the presence of two different set of signals including, in particular, two spin-systems at δ 5.46, br s, (-N*H*CHO) and δ 8.24, d, *J* = 12 Hz, (-NHC*H*O); and at δ 5.02, br s, (-N*H*CHO) and δ 8.03, d, *J* = 2 Hz, (-NHC*H*O) that were attributed by the coupling constant value of the formyl proton to *E* and *Z* forms, respectively, of a formamido moiety. Two set of signals were also observed for the nearby protons and carbons (Table 1 and Table 2). Detailed analysis of COSY, HSQC, and HMBC spectra allowed the complete definition of the structure of **9** indicating that it was the formamido derivative of amphilectene **5**. All NMR values for both isomers were assigned (Table 1 and Table 2). The NMR spectra of **9** were also recorded in C_6_D_6_ (Materials and Methods). In this solvent, the signals of two isomers were almost indistinguishable except for the -NHCHO moiety. With the aim of further studying the junction stereochemistry and thus the relative configuration of C-8, C-13, C-12 and C-4, a detailed analysis of the coupling constants of the junction protons was undertaken by ^1^H NMR homo-decoupling experiments being the proton spectra of formamido derivative **9**, in both CDCl_3_ and C_6_D_6_, more resolved with respect to those of the corresponding isonitrile **5**. The examination in C_6_D_6_ solution of the multiplicity of H-13 was particularly diagnostic. Decoupling of H-8 (δ 2.66) resulted in the simplification of H-13 (δ 1.84) to a double doublet (*J* = 11 and 4 Hz) indicating the presence of a small *J*_H8-H13_ that was consistent with the 8,13-*cis* junction. Similarly, H-13 signal was again simplified to a double doublet (*J* = 11 and 2 Hz) by irradiation of the multiplet a δ 2.14 (H-12) implying a small *J*_H12-H13_ in agreement the 12,13-*cis* junction. On the other side, H-13 signal was modified to a sharp multiplet (*w*_1/2_ = 12 Hz) by irradiation of the signal at δ 0.96 (H-4) clearly indicating that the large coupling constant (*J* = 11 Hz) observed in the H-13 signal was due to H_4_-H_13_ coupling according to the 4,13-*trans* junction. To further support the proposed relative configuration a NOESY experiment was recorded on compound **9** in C_6_D_6_. Diagnostic and unambiguous steric correlations were observed between H-12 and both H-8 and H-13, as well as between the formyl proton (–NHC*H*O, δ 8.17) and H-4. By these data, the structure of compound **9** was established as (1R*,3S*,4R*,7S*,8R*,12R*,13S*)-7-formamidoamphilecta-10,14-diene and, consequently, the corresponding isonitrile **5** was confirmed to have the same stereochemistry. In addition, the chemical relationship between **5** and **9** was secured by acid hydrolysis of compound **5** that gave a formamido compound identical to natural **9**.

7-Isocyanoamphilecta-1,14-diene (**6**) was isomeric with **5** having the same molecular formula C_21_H_31_N as it was deduced by MS spectra [**m*/*z** 320 (M + Na)^+^]. Analysis of ^1^H and ^13^C NMR spectra of **6** indicated structural features similar with **5** (Table 1 and Table 2), including the isobutenyl moiety and an endocyclic double bond. In particular, two vinyl methyls [δ_H_ 1.76 (br s, H_3_-16) and 1.81 (br s, H_3_-17); δ _C_ 26.7 (q, C-16) and 19.5 (q, C-17)], two secondary methyls linked to sp^3^ carbon atoms [δ_H_ 1.05 (d, *J* = 6 Hz, H_3_-18) and 0.82 (d, *J* = 7 Hz, H_3_-20); δ_C_ 19.9 (q, C-18) and 17.2 (q, C-20)], and one methyl linked to a non-protonated carbon bearing the isonitrile group [δ_H_ 1.54 (s, H_3_-19); δ_C_ 26.0 (q, C-19)] could be recognized. Analysis of 2D NMR experiments indicated that the double bonds were conjugated and allowed to locate one of them in the isobutenyl moiety (Δ^14,15^) and the second one necessarily in A ring at C-1 (Δ^1,2^). The carbon resonances of the ring C as well as angular C-12 (δ 44.2) indicated a δ_C_ pattern comparable with **5** (Table 2) strongly suggesting the same 8,13-*cis* and 12,13-*cis* ring junctions. The α-orientation of H_3_-18, the same as all amphilectenes in the literature, was indicated by both the typical C-18 chemical shift (δ 19~20) and the H-3 multiplicity (*w*_1/2_ = 20 Hz) measured in the ^1^H NMR spectrum (C_6_D_6_) and indicating the axial orientation. The relative configuration of C-11 was suggested by carbon chemical shifts of the methyl C-20 (δ_C_ 17.2) and the methylene C-9 (δ_C_ 18.1) (Table 2) consistent with the axial orientation of H_3_-20. Interestingly, the carbon values of **6** showed a close resemblance with ^13^C NMR data, if unassigned, of 7-isocyano-l-cycloamphilectene [25] containing the tricyclic partial structure the same as **6** further supporting the structural hypothesis. Thus, compound **6** was proposed as (3R*,4R*,7S*,8R*,11R*,12S*,13S*)-7-isocyanoamphilecta-1,14-diene. A formamido derivative with the same terpenoid gross structure of **6** was previously reported in the literature [30] but no spectroscopic data and chemical characterization was described in that paper.

7-Isocyanoamphilecta-11(20),14-diene (**7**) had the molecular formula C_21_H_31_N, the same as co-occurring amphilectenes **4**–**6**. Spectroscopic data of **7** (Table 1 and Table 2) closely resembled those of **5**, in particular indicating that the two compounds only differed in the presence of an exocyclic double bond (Δ^11(20)^) rather than Δ^10^ unsaturation, respectively. Consistent with this, the ^1^H NMR spectrum contained two olefinic signals [δ_H_ 4.95 (br s, H_-_20a) and 4.79 (br s, H-20b)] long-range correlated with both H_2_-10 [δ_H_ 2.05 (m, H-10a) and 2.43 (m, H-10b)] and H-12 angular methine at δ_H_ 1.99 (m). All other structural features including the relative configuration of stereogenic centers was established to be the same as **5** by comparison of carbon NMR values.

Finally, 7-formamidoamphilecta-11(20),15-diene (**8**) was characterized as the formamido derivative of co-occurring amphilectadiene **4**. The ESIMS spectrum contained a sodiated molecular peak at **m*/*z** 338 (M+Na)^+^ consistent with the molecular formula C_21_H_33_NO. The ^1^H and ^13^C NMR spectra (Table 1 and Table 2) of **8** immediately revealed a close structural relationship with compound **4** with the carbon skeleton exhibiting two exocyclic double bonds (Δ^11(20)^ and Δ^15^). In addition, the presence in the molecule of a -NHCHO functional group, the same as compound **9**, was evident by typical double sets of signals in the NMR spectra (Table 1 and Table 2) that were attributed to the *E* and *Z* formamido isomers. Analysis of 2D NMR experiments (COSY, HSQC, and HMBC) allowed the definition of the gross structure **8** whereas the comparison of the carbon chemical shift pattern with those of model amphilectenes evidenced the strict similarity with **4**. Analogous with the pair isonitrile **5**/ formamido **9**, the chemical relationship between **4** and **8** was confirmed by conversion of isonitrile amphilectadiene **4** into the corresponding formamido derivative by acid hydrolysis. The product obtained was identical with natural **8**.

**Figure 5:**
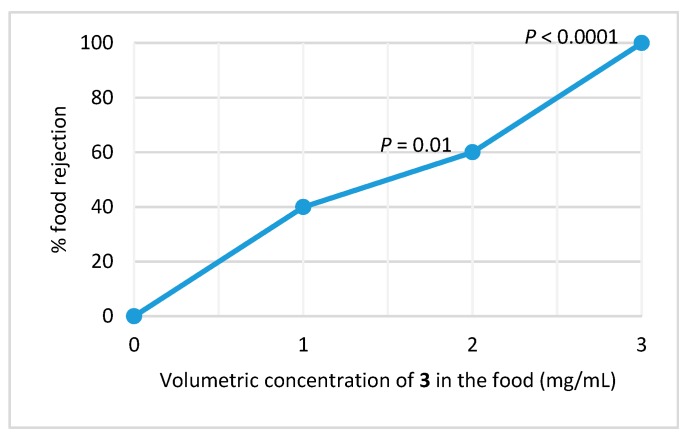
Feeding deterrence dose–response curve against *P. elegans*. The significant differences (control vs. treatment) were evaluated using the two-tailed Fisher’s exact test (n = 10 for each tested concentration, α = 0.05).

The amphilectene mixture turned out to be mainly localized in the nudibranch’s mantle suggesting the possible involvement of these molecules in the nudibranch’s defensive strategies. This hypothesis was confirmed by testing the feeding deterrent properties of main component **3** against the generalist shrimp *Palaemon elegans* [31]. A minimum dose effect was observed at a volumetric concentration of 2.0 mg/mL of **3** in the food, which significantly deterred the shrimp from feeding (*p* = 0.01), while a very highly significant rejection response was obtained when testing **3** at a concentration of 3.0 mg/mL (*p* < 0.0001) (Figure 5).

## 3. Discussion

**Scheme 1:**
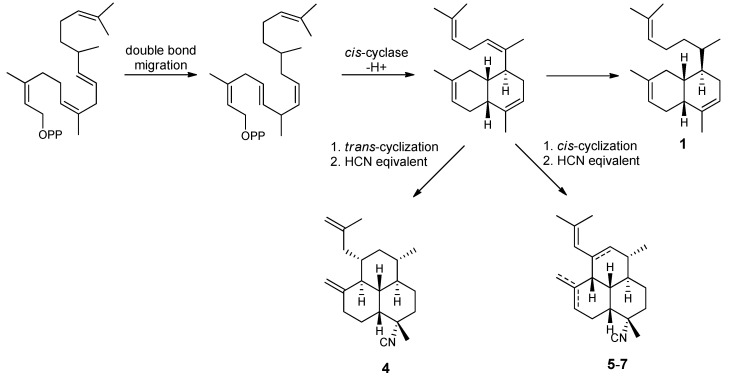
Proposed biogenetical pathway leading to amphilectadienes **4**–**7**.

Terpene isonitriles often co-occurring with oxidized derivatives with formamido, isocyanate, and isothiocyanate functionalities are secondary metabolites of a restricted group of marine sponges [8,9,14]. The biosynthesis of these compounds has been investigated and the incorporation of inorganic cyanide into the terpene metabolites has been demonstrated for some sponge species. By contrast, the occurrence of these compounds in nudibranchs, such as Phyllidiids, has been proven to arise by uptake from dietary sponges [8,32]. However, among diterpene isonitriles, those having the amphilectene skeleton have been isolated from a number of sponges belonging to the order Halichondrida [8,9] whereas the finding of compound **3** in *Phyllidiella pustulosa* is the only report of amphilectenes from nudibranchs to date [33]. Therefore, this work resulting in the isolation of a complex mixture of amphilectenes from two different collections of *P. coelestis* is, in our opinion, notable. *Phyllidia* amphilectenes derive likely from one or more dietary sponges. Unfortunately, both nudibranch collections were made on the rocks and no sponge from the same habitat was collected together with the mollusks. The structures of new compounds exhibit unusual stereochemical features. In particular, the structural arrangement of compounds **5**–**7** and **9** with both 8,13-*cis* and 12,13-*cis* ring junctions is unprecedented in the amphilectane skeleton whereas it has been already found and secured by X-ray analysis for the cycloamphilectane framework [25]. In addition, amphilectadienes **4** and **8** and cycloamphilectene **2** also display the 8,13-*cis* junction. This is in agreement with the stereochemistry of co-occurring *cis*-fused bifloradiene **1** which could be formed after the first cyclization step from a *cis*-bifloratetraene precursor that should further originate in all other amphilectene metabolites (Scheme 1). The introduction of cyanide group at C-7 or both C-8 and C-15 decorating all isolated amphilectenes could occur subsequently on a suitable tricyclic intermediate formed by *trans*- or *cis*-cyclization biosynthetic step (Scheme 1).

The co-occurrence of formamido derivatives **8**–**10** is in agreement with the previous reports [14]. The formation of formamido function from isonitrile group has been suggested to originate through either enzymatic or non-enzymatic way even though the latter seems to be more probable [8,32].

A series of various and interesting biological properties, notably antibiotic, cytotoxic and anti-malarian, have been described for marine terpenes, including amphilectenes, containing the –NC group or related functionalities [9,14]. In particular, known amphilectenes **3**, **4**, and **10** displayed interesting antiproliferative activity against apoptosis-sensitive and apoptosis-resistant cancer cell lines [26] and anti-neuroinflammatory activity being potent inhibitors of rat brain microglia thromboxane B_2_ generation [34]. Compound **3** was also shown to have immunosuppressive effects on mononuclear blood cells [23]. New amphilectadienes **5**–**9** isolated in this work were obtained in a small amount and this prevented any biological activity evaluation.

The ecological role played by terpene isonitriles and derivatives in nudibranchs has been also investigated by testing either feeding deterrence or ichthyotoxicity against fishes or crustaceans [8,32]. In this study we have found that compound **3** and all other co-occurring amphilectenes were significantly more abundant in the mantle with respect to the internal organs of *P. coelestis*, and this suggested their involvement in chemical defense. The ability of nudibranchs to selectively store defensive chemicals in the mantle or in specific anatomical parts on the mantle has been observed, in fact, for a number of nudibranch species and rigorously proven in chromodorids [15]. Accordingly, what emerges from this study is that the main metabolite **3**, which is by far the most abundant metabolite in the mantle of *P. coelestis*, is able to induce rejection of food in a generalist predator, the shrimp *P. elegans* [31], in a dose-dependent manner.

## 4. Materials and Methods

### 4.1. General Procedures

Optical rotations were measured on a Jasco DIP 370 digital polarimeter. NMR experiments were recorded at the ICB-NMR Service Centre on a Bruker DRX 600 MHz spectrometer equipped with a TXI CryoProbe, a Bruker WM 500, a Bruker Avance-400 spectrometer using an inverse probe fitted with a gradient along the z-axis, on a Bruker Avance III HD spectrometer quipped with a CryoProbe Prodigy and on a Bruker WM 300. The NMR spectra were acquired in CDCl_3_ and C_6_D_6_, reporting the chemical shifts in parts per million referred to the solvent (CHCl_3_ δ*_H_* 7.26 and δ*_C_* 77.0; C_6_H_6_ δ*_H_* 7.15 and δ*_C_* 128.0). ESIMS was performed on a Micromass Q-TOF MicroTM coupled with a HPLC Waters Alliance 2695. The instrument was calibrated by using a PEG mixture from 200 to 1000 MW (resolution specification 5000 FWHM, deviation <5 ppm RMS in the presence of a known lock mass). HRESIMS spectra were acquired on a Q-Exactive hybrid quadrupole-orbitrap mass spectrometer (Thermo Scientific). TLC were performed on precoated silica gel plates (Merck Kieselgel 60 F254, 0.25 mm), with detection provided by ultraviolet (UV) light (254 nm) and by spraying with CeSO_4_ reagent followed by heating (120 °C). Silica gel column chromatography was performed using Merck Kieselgel 60 powder (0.063−0.200mm). HPLC separations were carried out using a Jasco LC PU-4180 liquid chromatograph equipped with an ultraviolet/visible (UV/Vis) detector (Jasco UV-4075) and a Waters liquid chromatograph equipped with a differential refractometer as detector.

### 4.2. Biological Material

Two populations of *P. coelestis* (10 and 9 individuals) were collected by scuba diving at a depth of 20 m along the coast of Hainan Island, in South China Sea, during January 2002 and 2013, respectively. Biological material was immediately frozen and then transferred to ICB in Naples, where was kept at –80 °C until extraction. The nudibranch was identified by one of us (E. Mollo). Pictures of individuals from both collections are included in Supplementary Material (Figure S54). Voucher samples for 2002 and 2013 collections were stored with codes HN-51 and XS-204 at the ICB and SIMM institutes, respectively.

### 4.3. Extraction and Isolation Procedures

The two collections of *P. coelestis* were analyzed separately by using the same procedure as reported below. Each individual of *P. coelestis* was carefully dissected into mantle and internal organs that were separately extracted at room temperature by acetone using ultrasound vibrations for 5 min. Filtration of the two homogenates gave respectively an aqueous Me_2_CO filtrate that was concentrated in vacuo until the evaporation of the organic solvent. The residual water was then diluted and extracted with diethyl ether (100 mL × 3). The organic phase from each extraction were opportunely combined obtaining after evaporation of the solvent the corresponding crude diethyl ether extracts of mantle [103 mg (from 2002 collection) and 97 mg (from 2013 collection)] and internal organs [97 mg (from 2001 collection) and 33 mg (from 2002 collection)]. All extracts were preliminary analyzed by TLC chromatography. The mantle ether extract (103 mg) from the first *P. coelestis* collection was subjected to a SiO_2_ chromatographic column eluted by an increase polarity gradient of light petroleum ether/diethyl ether yielding ten fractions (A–J) four of which (A–C, and I) resulted in containing amphilectane metabolites by ^1^H NMR analysis. Fractions A and C, eluted with light petroleum ether/diethyl ether 98:2 and 95:5, contained pure compounds **1** (2 mg) and **3** (18 mg), respectively. Fractions B and I were submitted to further purification steps by HPLC. In particular, fraction Bwas fractionated on *n*-phase column Spherisorb S5W (300 × 3.9 mm, 5 μm) by using an isocratic mode elution (*n*-hexane/ethyl acetate, 99.5:0.5; flow rate 0.6 mL/min; RI detector) affording pure compounds **2** (1.0 mg) and **4** (1.4 mg; Rt 7 min) along with a peak at Rt = 9.2 min which was further purified giving pure compounds **5** (4.0 mg), **6** (3.0 mg), and **7** (2.2 mg) (Spherisorb S5W column; *n*-hexane/ethyl acetate, 99.8:0.2; flow rate 0.7 mL/min; RI detector). Finally, fraction I was purified on a Supelco C18 Ascentis column (250 × 10 mm, 5 μm) eluted in isocratic mode (MeOH/H_2_O, 9:1; flow rate 2 mL/min, UV detector at λ = 210 and 254 nm) obtaining pure compounds **8** (2.0 mg), **9** (3.0 mg), and **10** (4.0 mg). This procedure was also used to fractionate the diethyl ether extract of internal parts of the first collection, as well as both external and internal part extracts from second *P. coelestis* population. In particular, the inner parts extract of the first collection gave: **1** (1.3 mg), **2** (1.5 mg), **3** (9.0 mg), **4** (2.0 mg), **5** (0.7 mg), **6** (0.4 mg), **7**–**10** (trace); the external part extract of the second collection gave **1** (1.5 mg), **2** (0.5 mg), **3** (6.0 mg), **4** (1.0 mg), **5** (2.0 mg), **6** (trace) and **7** (trace), **8** (1.5 mg), **9** (2.0 mg) **10** (2.0 mg); the internal part extract of the second collection gave **1** (0.3 mg), **2** (1.0 mg), **3** (4.0 mg), **4** (1.0 mg), **5** (0.5 mg), **6** (0.4 mg), **7**-**10** (trace).

**Compound 1.** [α]_D_ = + 22.3 (*c* 0.2, CHCl_3_); ^1^H and ^13^C NMR data in agreement with literature [22]. NMR spectra in Figures S1–S4. ESIMS *m*/*z* 295 [M + Na]+.

**Compound 2.** [α]_D_ = +25.2 (*c* 0.05, CHCl_3_); ^1^H NMR (CDCl_3_, 400 MHz) δ 2.01 (1H, m, H-1), 1.97 (1H, m, H-9a), 1.95 (2H, m, H_2_-10), 1.81 (4H, m, H-2a, H-5a and H_2_-6), 1.77 (1H, m, H-20a), 1.71 (1H, m, H-13) 1.67 (1H, m, H-8), 1.55 (3H, s, H_3_-19), 1.53 (1H, m, H-20b), 1.47 (1H, m, H-14a), 1.45 (1H, m, H-9b), 1.27 (1H, m, H-3), 0.99 (1H, m, H-5b), 0.97 (1H, m, H-4), 0.92 (3H, s, H_3_-17), 0.86 (3H, d, *J* = 7 Hz, H_3_-18), 0.89 (1H, m, H-14b), 0.81 (3H, s, H_3_-16), 0.75 (1H, m, H-2b); ^13^C NMR (CDCl_3_, 400 MHz) δ 132.1 (C, C-12), 126.2 (C, C-11), 61.3 (C, C-7), 45.6 (CH_2_, C-2), 44.3 (CH, C-8, and CH_2_, C-14), 44.0 (CH_2_, C-20), 43.5 (CH, C-4), 41.1 (CH, C-13), 38.3 (CH, C-3), 35.7 (CH, C-1), 34.1 (CH_2_, C-6)_,_ 31.6 (CH_2_, C-10), 31.3 (CH_3_, C-17), 30.3 (C, C-15), 26.9 (CH_3_, C-19), 25.4 (CH_2_, C-5), 24.4 (CH_3_, C-16), 21.3 (CH_2_, C-9), 19.0 (CH_3_, C-18); NMR spectra in Figures S5–S8. ESIMS *m*/*z* 320 [M + Na]+.

**Compound 3.** [α]_D_ = –53.6 (*c* 0.5, CHCl_3_); ^1^H and ^13^C NMR data in agreement with literature [23,24]. NMR spectra in Figures S9 and S10. ESIMS *m*/*z* 347 [M + Na]^+^.

**Compound 4.** [α]_D =_ + 35.5 (*c* 0.3, CHCl_3_); ^1^H and ^13^C NMR data are reported in Table 1 and Table 2 and are in agreement with literature [23,24]. NMR spectra in Figures S11-S14. ESIMS *m*/*z* 320 [M + Na]^+^.

**Compound 5.** [α]_D_ = –18.9 (*c* 0.1, CHCl_3_); ^1^H NMR (C_6_D_6_, 400 MHz) δ 5.55 (1H, bs, H-14), 5.33 (1H, bs, H-10), 2.95 (1H, m, H-1), 2.35 (1H, m, H-9a eq), 2.04 (1H, m, H-9b ax), 2.00 (1H, m, H-12), 1.78 (1H, m, H-6a), 1.67 (3H, bs, H_3_-16), 1.65 (3H, bs, H_3_-20), 1.59 (3H, bs, H_3_-17), 1.570 (1H, m, H-8), 1.565 (1H, m, H-13), 1.46, (1H, m, H-5a), 1.43 (1H, m, H-6b), 1.31 (1H, ddd, *J* = 12.0, 3.0, 2.5 Hz, H-2a), 1.17 (1H, m, H-3), 1.13 (3H, s, H_3_-19) 1.06 (1H, ddd, *J* = 12.0, 12.0, 4 Hz, H-2b), 0.76 (1H, m, H-4), 0.70 (3H, d, *J* = 6.0 Hz, H_3_-18), 0.51 (1H, m, H-5b); ^13^C NMR (C_6_D_6_, 400 MHz) δ 133.6 (C, C-11), 131.9 (C, C-15), 127.9 (CH, C-14), 122.4 (CH, C-10), 60.5 (C, C-7), 44.7 (CH, C-12), 42.7 (CH, C-8), 37.3 (CH, C-4), 35.7 (CH_2_, C-2), 34.9 (CH, C-13), 34.2 (CH_2_, C-6), 33.1 (CH, C-1), 31.7 (CH, C-3), 27.0 (CH_2_, C-5), 26.2 (CH_3_, C-19), 26.0 (CH_3_, C-16), 25.0 (CH_2_, C-9), 20.9 (CH_3_, C-20), 19.7 (CH_3_, C-18), 17.7 (CH_3_, C-17); NMR spectra in Figures S15–S22. ESIMS *m*/*z* 320 [M + Na]^+^; HRESIMS *m*/*z* 320.2352 (calcd for C_21_H_31_NNa 320.2349).

**Compound 6.** [α]_D_ value not provided due to degradation of compound before measurement. ^1^H NMR (C_6_D_6_, 500 MHz) δ 5.50 (1H, br s, H-14), 5.36 (1H, br s, H-2), 1.90 (1H, m, H-9b), 1.87 (1H, m, H-11), 1.82 (1H, m, H-12), 1.80 (3H, s, H_3_-16), 1.70 (1H, m, H-6b), 1.72 (3H, s, H_3_-17), 1.58 (2H, m, H-3 and H-9b), 1.45 (1H, m, H-6a), 1.43 (2H, m, H_2_-5), 1.40 (1H, m, H-13), 1.37 (1H, m, H-9a), 1.36 (1H, m, H-10 a), 1.35 (1H, m, H-8), 1.12 (3H, s, H_3_-19), 1.02 (1H, m, H-4), 0.90 (3H, d, *J* = 6 Hz, H_3_-18), 0.78 (3H, d, *J* = 7Hz, H_3_-20); ^13^C NMR (C_6_D_6_, 300 MHz) δ 152.7 (NC), 137.0 (C, C-15), 131.4 (CH, C-2), 127.0 (CH, C-14), 126.6 (C, C-1), 62.0 (C, C-7), 46.4 (CH, C-8), 44.8 (CH, C-12) 38.5 (CH, C-3), 37.5 (CH, C-13), 36.2 (CH, C-4), 34.3 (CH_2_, C-6), 32.2 (CH_2_, C-10), 29.4 (CH, C-11), 27.6 (CH_2_, C-5), 26.6 (CH_3_, C-16), 26.4 (CH_3_, C-19), 19.9 (CH_3_, C-8), 19.6 (CH_3_, C-17), 18.4 (CH_2_, C-9), 17.4 (CH_3_, C-20). NMR spectra in Figures S23–S32. ESIMS *m*/*z* 320 [M + Na]^+^; HRESIMS *m*/*z* 320.2346 (calcd for C_21_H_31_NNa 320.2349).

**Compound 7.** [α]_D_ = – 16.2 (*c* 0.1, CHCl_3_); ^1^H and ^13^C NMR data are reported in Table 1 and Table 2. NMR spectra in Figures S33–S37. ESIMS m/**z** 320 [M + Na]^+^; HRESIMS *m*/*z* 320.2351 (calcd for C_21_H_31_NNa 320.2349).

**Compound 8.** [α]_D_ + 31.9 (*c* 0.12, CHCl_3_); ^1^H and ^13^C NMR data are reported in Table 1 and Table 2. NMR spectra in Figures S38–S42. ESIMS *m*/*z* 338 [M + Na]^+^; HRESIMS *m*/*z* 338.2465 (calcd for C_21_H_33_NONa 338.2460).

**Compound 9.** [α]_D_ = – 14.0 (*c* 0.4, CHCl_3_); ^1^H NMR (C_6_D_6_, 400 MHz) δ 8.17 (0.5H, d, *J* = 12 Hz, NHC*H*O *E* isomer), 7.65 (0.5H, d, *J* = 1 Hz, NHC*H*O *Z* isomer), 5.66 (1H, bs, H-14), 5.43 (1H, bs, H-10), 4.91 (0.5H, br s, N*H*CHO *E* isomer), 3.80 (0.5H, br s, N*H*CHO *Z* isomer), 3.01 (1H, m, H-1), 2.66 (1H, m, H-8), 2.14 (1H, m, H-12), 1.88 (2H, m, H_2_-9), 1.84 (1H, ddd, *J* = 11, 4 and 2 Hz, H-13), 1.72 (3H, bs, H_3_-20), 1.69 (3H, bs, H_3_-16), 1.57 (3H, bs, H_3_-17), 1.49 (3H, s, H_3_-19), 1.38 (1H, m, H-6a), 1.32 (1H, m, H-3), 1.18 (1H, m, H-5a), 1.17 (1H, m, H-2a), 1.14 (1H, m, H-6b), 1.04 (1H, m, H-2b), 0.96 (1H, m, H-4), 0.82 (3H, d, *J* = 6.0 Hz, H_3_-18), 0.66 (1H, m, H-5b); ^13^C NMR (C_6_D_6_, 400 MHz) δ 164.5 (NHCHO, *E* isomer), 160.1 (NHCHO, *Z* isomer), 134.7 (C, C-11), 131.6 (C, C-15), 127.9 (CH, C-14), 122.8 (CH, C-10), 44.8 (CH, C-12), 40.6 (CH, C-8), 38.1 (CH, C-4), 36.2 (CH_2_, C-6), 35.0 (CH, C-13), 33.4 (CH, C-1), 33.2 (CH_2_, C-2), 32.2 (CH, C-3), 25.6 (CH_2_, C-5), 25.7 (CH_3_, C-16), 24.3 (CH_2_, C-9), 23.0 (CH_3_, C-19), 21.1 (CH_3_, C-20), 19.9 (CH_3_, C-18), 17.6 (CH_3_, C-17); NMR spectra in Figures S43–S50. ESIMS *m*/*z* 338 [M + Na]^+^; HRESIMS *m*/*z* 338.2457 (calcd for C_21_H_33_NONa 338.2460).

**Compound 10**. [α]_D_ = –37.5 (*c* 0.15, CHCl_3_); selected ^1^H NMR signals (CDCl_3_, 400 MHz) δ 8.25 (0.5 H, d, *J* = 12 Hz, NHC*H*O *E* isomer), 8.05 (0.5 H, d, *J* = 2 Hz, NHC*H*O *Z* isomer), 5.51 (0.5 H, br d, *J* = 12 Hz, N*H*CHO *E* isomer), 5.16 (0.5 H, br s, N*H*CHO *Z* isomer), 4.87 (1H, s, H-20a *E* isomer and H-20a *Z* isomer), 4.64 (0.5 H, s, H-20b *Z* or *E* isomer), 4.56 (0.5 H, s, H-20b *Z* or *E* isomer), 1.43 (3H, s, H_3_-16), 1.37 (3H, s, H_3_-17), 0.99 (3H, d, *J* = 6 Hz, H_3_-19), 0.90 (3H, d, *J* = 6 Hz, H_3_-18); ^13^C NMR (CDCl_3_, 300 MHz) δ 162.7 (NHCHO, *E* isomer), 160.4 (NHCHO, *Z* isomer), 156.4 (NC) 150.5 (C, C-11), 105.6 (CH_2_, C-20), 67.2 (C, C-8), 55.7 (CH, C-13), 54.0 (C, C-15 *Z* or *E* isomer), 53.0 (C, C-15 *E* or *Z* isomer), 48.2 (CH_2_, C-14 *E* isomer), 46.2 (CH, C-12), 45.2 (CH_2_, C-14 *Z* isomer), 42.6 (CH, C-4), 41.9 (CH_2_, C-2), 40.8 (CH, C-7), 39.7 (CH_2_, C-9), 35.6 (CH, C-3), 33.8 (CH_2_, C-10), 31.8 (CH, C-1), 30.7 (CH_3_, C-17 *Z* isomer), 29.7 (CH_2_, C-5), 29.4 (CH_2_, C-6), 29.2 (CH_3_, C-16 *E* or *Z* isomer), 29.0 (CH_3_, C-17 *E* isomer), 28.7 (CH_3_, C-16 *Z* or *E* isomer), 19.5 (CH_3_, C-18), 15.3 (CH_3_, C-19); NMR spectra in Figures S51–S53. ESIMS *m*/*z* 365 [M + Na]^+^.

### 4.4. Acid Hydrolysis of Compounds 4 and 5

Aliquots of isonitriles **4** (1 mg) and **5** (1mg) were dissolved in methanol (2 mL) and three drops of AcOH were added to each solution. The reaction mixtures were stirred at room temperature for 24 h according to the literature [35]. After concentration, the reaction products were purified on a Supelco C18 Ascentis column (250 × 10 mm, 5 μm) eluted in isocratic mode (MeOH/H_2_O, 9:1; flow rate 2 mL/min, UV detector at λ = 210 and 254 nm) obtaining pure compounds **8** (0.5 mg) and **9** (0.4 mg), respectively.

### 4.5. Feeding Deterrence Assay

Compound **3** was tested for its feeding deterrence activity against the shrimp *P. elegans* according to the procedures described in the literature [15,31]. The zero concentration was defined as control and significant differences in the consumption of treated vs. control pellets have been evaluated by two-tailed Fisher’s exact test (α  =  0.05, *n*  =  10 for each concentration tested).

## Figures and Tables

**Table 1:** ^1^H nuclear magnetic resonance (NMR) data^a-,d^ of compunds **5**–**9**.

H	5	6	7	8	9
	δ_H_ (*J* in Hz)	δ_H_ (*J* in Hz)	δ_H_ (*J* in Hz)	δ_H_ (*J* in Hz)	δ_H_ (*J* in Hz)
1	2.93, m	-	2.98, app. br s	1.77, m	2.91, app. t (9)
2	1.07 eq, ddd, (13,4,1) 1.29 ax, m	5.32, br s	1.29, m 1.57, m	0.68, m 1.92, m	1.07 eq, ddd, (13, 4, 2) 1.29, m
3	1.28, m	1.85, m	1.31, m	1.19, m	1.28, m
4	0.95, m	1.28, m	1.04, m	0.98, m	0.95, m
5	0.93, m 1.89, m	0.93, m 1.30, m	1.06, m 1.88, m	1.10, m 1.87, m	1.11, m 1.90, m
6	1.80, m 2.05, m	1.76, m 1.95, m	1.74, m 1.90, m	1.55, m	1.50, m (*1.60, m*)
8	1.82, m	1.63, m	1.68, m	2.02, m (*1.99*, *m*)	1.75, m (2.44, *m*)
9	2.14, m 2.27, m	1.56, m	1.60, m 1.97, m	1.52, m	1.95, m
10	5.44, m	1.73, m	2.05, m 2.43, m	2.19, m 2.36, m	5.40, br d (7)
11	-	1.94, m	-		-
12	2.01, m	1.99, m	1.99, m	1.53, m	2.03, m
13	1.70, m	1.70, m	1.87, m	1.24, m	1.80, m (*1.92*, *m*)
14	5.46, m	5.44, s	5.48, br d (8)	2.65, m 1.44, m	5.48, br d (9)
16	1.72, br s	1.76, br s	1.72, br s	4.63, br s 4.73, br s	1.72,br s
17	1.65, br s	1.81, br s	1.61, br s	1.71, br s	1.64, br s
18	0.82, d (6)	1.05, d (6)	0.82, d (7)	0.89, d (6)^e^ [*0.88*, *d (6)*]^e^	0.82, d (7)
19	1.57, s	1.54, s	1.58, s	1.34, s (*1.45*, *s*)	1.50, s (*1.59, s*)
20	1.72, br s	0.82, d (7)	4.79, br s 4.95, br s	4.62, br s 4.87, br s	1.69, s
NHC*H*O	-	-	-	8.25 d (12) [*8.09*, *d*, *(2)*]	8.24, d (12) [*8.03*, *d (2)*]
N*H*CHO	-	-	-	5.08 (*5.66*)	5.46, br s [*5.02*, *s*]

^a^ The spectra were recorded in CDCl_3_. ^b^Assignments aided by ^-^COSY, HSQC, and HMBC (*J* = 7 Hz) experiments. ^c^ Coupling constants determined by homodecoupling experiments. ^d^ Values in brackets refer to the *E* isomer. ^e^ Interchangeable values.

**Table 2:** ^13^C NMR data^a^ of compunds **5**–**9**.

C	5		6		7		8		9	
	δ_C_	Type ^b^	δ_C_	Type ^b^	δ_C_	Type ^b^	δ_C_	Type ^b^	δ_C_	Type ^b^
1	32.8	*CH*	125.7	*C*	33.1	*CH*	34.0	*CH*	33.5	*CH*
2	35.5	*CH_2_*	131.1	*CH*	35.6	*CH_2_*	41.5	*CH_2_*	35.7	*CH_2_*
3	31.3	*CH*	38.2	*CH*	33.9	*CH*	38.2	*CH*	31.3	*CH*
4	37.5	*CH*	36.2	*CH*	36.5	*CH*	42.9	*CH*	37.6	*CH*
5	26.6	*CH_2_*	27.6	*CH_2_*	26.6	*CH_2_*	23.0	*CH_2_*	26.7	*CH_2_*
6	33.8	*CH_2_*	34.1	*CH_2_*	33.9	*CH_2_*	32.4	*CH_2_*	33.1 (*34.8*)	*CH_2_*
7	61.8	*C*	64.0	*C*	60.9	*C*	54.0 (*56.1*)	*C*	54.0 (*56.9*)	*C*
8	43.8	*CH*	46.3	*CH*	46.2	*CH*	42.7 (*40.4*)	*CH*	44.6 (*40.8*)	*CH*
9	24.8	*CH_2_*	18.1	*CH_2_*	24.6	*CH_2_*	19.0	*CH_2_*	24.4	*CH_2_*
10	121.9	*CH*	32.1	*CH_2_*	36.3	*CH_2_*	33.3	*CH_2_*	122.1	*CH*
11	134.2	*C*	29.0	*CH*	nd	*-*	147.3	*C*	134.4	*C*
12	44.5	*CH*	44.2	*CH*	46.6	*CH*	49.8	*CH*	44.6	*CH*
13	34.5	*CH*	37.4	*CH*	37.5	*CH*	44.7	*CH*	35.3 (*37.9*)	*CH*
14	127.3	*CH*	126.0	*CH*	127.9	*CH*	41.3	*CH_2_*	127.8	*CH*
15	132.3	*C*	134.0	*C*	130.5	*C*	144.0	*C*	130.9	*C*
16	25.8	*CH_3_*	26.7	*CH_3_*	26.0	*CH_3_*	20.9	*CH_3_*	26.1	*CH_3_*
17	17.7	*CH_3_*	19.5	*CH_3_*	17.8	*CH_3_*	109.1	*CH_2_*	17.8	*CH_3_*
18	19.6	*CH_3_*	19.9	*CH_3_*	19.7	*CH_3_*	18.4	*CH_3_*	20.0	*CH_3_*
19	26.4	*CH_3_*	26.0	*CH_3_*	26.4	*CH_3_*	27.1 (*23.9*)	*CH_3_*	26.8 (*23.6*)	*CH_3_*
20	21.0	*CH_3_*	17.2	*CH_3_*	108.1	*CH_2_*	104.6	*CH_2_*	21.1	*CH_3_*
NC	nd	*-*	153.4	*C*	153.1	*C*	-	*-*	-	*-*
NHCHO	-		-		-		162.7 (*159.9*)	*CH*	162.8 (*160.0*)	*CH*

^a^ The spectra were recorded in CDCl_3_. ^b^ Assignments aided by COSY, HSQC, and HMBC (*J* = 7 Hz) experiments.

## Supplementary Materials

The following are available online at https://www.mdpi.com/1660-3397/17/11/603/s1: Figures S1–S53: 1D-and 2D-NMR spectra of compounds **1**–**10**.

## References

[1] Bouchet P., Rocroi J.-P., Hausdorf B., Kaim A., Kano Y., Nützel A., Parkhaev P., Schrödl M., Strong E.E. (2017). Revised classification, nomenclator and typification of gastropod and monoplacophoran families. Malacologia.

[2] Cimino G., Ghiselin M.T. (2009). Chemical defense and the evolution of opisthobranch gastropods. Proc. Calif. Acad. Sci..

[3] Bornancin L., Bonnard I., Mills S.C., Banaigs B. (2017). Chemical mediation as a structuring element in marine gastropod predator-prey interactions. Nat. Prod. Rep..

[4] Cimino G., Fontana A., Gavagnin M. (1999). Marine opisthobranch molluscs: Chemistry and ecology in sacoglossans and dorids. Curr. Org. Chem..

[5] Cimino G., Ciavatta M.L., Fontana A., Gavagnin M., Tringali C. (2001). Metabolites of marine opisthobranchs: chemistry and biological activity. Bioactive Compounds from Natural Sources.

[6] Dean L.J., Prinsep M.R. (2017). The chemistry and chemical ecology of nudibranchs. Nat. Prod. Rep..

[7] Burreson B.J., Scheuer P.J., Finer J., Clardy J. (1975). Isocyanopupukeanane, a marine invertebrate allomone with a new sesquiterpene skeleton. J. Am. Chem. Soc..

[8] Garson M.J., Simpson J.S. (2004). Marine isocyanides and related natural products—structure, biosynthesis and ecology. Nat. Prod. Rep..

[9] Emsermann J., Kauhl U., Opatz T. (2016). Marine isonitriles and their related compounds. Mar. Drugs.

[10] Wright A.D., Wang H., Gurrath M., König G.M., Kocak G., Neumann G., Loria P., Foley M.L., Tilley J. (2001). Inhibition of heme detoxification processes underlies the antimalarial activity of terpene isonitrile compounds from marine sponges. J. Med. Chem..

[11] Young R.M., Adendorff M.R., Wright A.D., Davies-Coleman M.T. (2015). Antiplasmodial activity: The first proof of inhibition of heme crystallization by marine isonitriles. Eur. J. Med. Chem..

[12] Fusetani N., Hirota H., Okino T., Tomono Y., Yoshimura E. (1996). Antifouling activity of isocyanoterpenoids and related compounds from a marine sponge and nudibranchs. J. Nat. Toxins.

[13] Wright A.D., McCluskey A., Robertson M.J., MacGregor K.A., Gordon C.P., Guenther J. (2011). Anti-malarial, anti-algal, anti-tubercular, anti-bacterial, anti-photosynthetic, and anti-fouling activity of diterpene and diterpene isonitriles from the tropical marine sponge *Cymbastela Hooperi*. Org. Biomol. Chem..

[14] Schnermann M.J., Shenvi R.A. (2015). Syntheses and biological studies of marine terpenoids derived from inorganic cyanide. Nat. Prod. Rep..

[15] Carbone M., Gavagnin M., Haber M., Guo Y.-W., Fontana A., Manzo E., Genta-Jouve G., Tsoukatou M., Rudman W.B., Cimino G. (2013). Packaging and delivery of chemical weapons: A defensive Trojan horse stratagem in chromodorid nudibranchs. PLoS ONE.

[16] Carbone M., Ciavatta M.L., Wang J.-R., Cirillo I., Mathieu V., Kiss R., Mollo E., Guo Y.-W., Gavagnin M. (2013). Extending the record of bis-γ-pyrone polypropionates from marine pulmonate mollusks. J. Nat. Prod..

[17] He W.-F., Li Y., Feng M.-T., Gavagnin M., Mollo E., Mao S.-C., Guo Y.-W. (2014). New isoquinolinequinone alkaloids from the South China Sea nudibranch Jorunna funebris and its possible sponge-prey Xestospongia sp.. Fitoterapia.

[18] Carbone M., Ciavatta M.L., Mathieu V., Ingels A., Kiss R., Pascale P., Mollo E., Ungur N., Guo Y.-W., Gavagnin M. (2017). Marine terpenoid diacylguanidines: structure, synthesis and biological evaluation of naturally occurring actinofide and synthetic analogs. J. Nat. Prod..

[19] Zhou Z.-F., Li X.-L., Yao L.-G., Li J., Gavagnin M., Guo Y.-W. (2018). Marine bis-γ-pyrone polypropionates of onchidione family and their effects on the XBP1 gene expression. Bioorg. Med. Chem. Lett..

[20] Wu Q., Chen W.-T., Li S.-W., Ye J.-Y., Huan X.-J., Gavagnin M., Yao L.-G., Wang H., Miao Z.-H., Li X.-W. (2019). Cytotoxic nitrogenous terpenoids from two South China Sea nudibranchs Phyllidiella pustulosa, Phyllidia coelestis, and their sponge-prey Acanthella cavernosa. Mar. Drugs.

[21] Wratten S.J., Faulkner D.J., Hirotsu K., Clardy J. (1978). Diterpenoid isocyanides from the marine sponge Hymeniacidon amphilecta. Tetrahedron Lett..

[22] Hirota H., Tomono Y., Fusetani N. (1996). Terpenoids with antifouling activity against barnacle larvae from the marine sponge Acanthella cavernosa. Tetrahedron.

[23] Ciavatta M.L., Fontana A., Puliti R., Scognamiglio G., Cimino G. (1999). Structures and absolute stereochemistry of isocyanide and isothiocyanate amphilectenes from the Caribbean sponge Cribochalina sp.. Tetrahedron.

[24] Ciavatta M.L., Gavagnin M., Manzo E., Puliti R., Mattia C.A., Mazzarella L., Cimino G., Simpson J.S., Garson M.J. (2005). Structural and stereochemical revision of isocyanide and isothiocyanate amphilectenes from the Caribbean marine sponge Cribochalina sp.. Tetrahedron.

[25] Molinski T.F., Faulkner D.J., Van Duyne G.D., Clardy J. (1987). Three new diterpene isonitriles from a Palauan sponge of the genus Halichondria. J. Org. Chem..

[26] Lamoral-Theys D., Fattorusso E., Mangoni A., Perinu C., Kiss R., Costantino V. (2011). Evaluation of the antiproliferative activity of diterpene isonitriles from the sponge Pseudoaxinella flava in apoptosis-sensitive and apoptosis-resistant cancer cell lines. J. Nat. Prod..

[27] König G.M., Wright A.D., Angerhofer C.K. (1996). Novel potent antimalarial diterpene isocyanates, isothiocyanates, and isonitriles from the tropical marine sponge Cymbastela hooperi. J. Org. Chem..

[28] Linden A., König G.M., Wright A.D. (1996). Four diterpene isonitriles from the sponge Cymbastela hooperi. Acta Cryst. Sect. C.

[29] Mitome H., Shirato N., Miyaoka H., Yamada Y., van Soest R.W.M. (2004). Terpene isocyanides, isocyanates, and isothiocyanates from the Okinawan marine sponge Stylissa sp.. J. Nat. Prod..

[30] Lucas R., Casapullo A., Ciasullo L., Gomez-Paloma L., Payá M. (2003). Cycloamphilectenes, a new type of potent marine diterpenes: Inhibition of nitric oxide production in murine macrophages. Life Sci..

[31] Mollo E., Gavagnin M., Carbone M., Castelluccio F., Pozone F., Roussis V., Templado J., Ghiselin M.T., Cimino G. (2008). Factors promoting marine invasions: A chemoecological approach. Proc. Natl. Acad. Sci. USA.

[32] Garson M.J., Simpson J.S., Flowers A.E., Dumdei E.J., Rahman A. (2000). Cyanides and thiocyanate-derived functionality in marine organisms—structures, biosynthesis and ecology. Studies in Natural Products Chemistry.

[33] Manzo E., Ciavatta M.L., Gavagnin M., Mollo E., Guo Y.-W., Cimino G. (2004). Isocyanide terpene metabolites of Phyllidiella pustulosa, a nudibranch from the South China Sea. J. Nat. Prod..

[34] Mayer A.M.S., Avilés E., Rodríguez A.D. (2012). Marine sponge Hymeniacidon sp. amphilectane metabolites potently inhibit rat brain microglia thromboxane B2 generation. Bioorg. Med. Chem..

[35] Avilés E., Rodríguez A.D., Vicente J. (2013). Two rare-class tricyclic diterpenes with antitubercular activity from the Caribbean sponge Svenzea flava. Application of vibrational circular dichroism spectroscopy for determining absolute configuration. J. Org. Chem..

