# Supplementary material for: Amphilectene Diterpene Isonitriles and Formamido Derivatives from the Hainan Nudibranch Phyllidia Coelestis
Source: Mar Drugs. 2019 Oct 24;17(11):603. doi: 10.3390/md17110603 (PMC6891729; doi:10.3390/md17110603)
Supplement: Supplementary file 1 [file marinedrugs-17-00603-s001.pdf]

Supplementary Material

Amphilectene diterpene isonitriles and formamido derivatives from the Hainan nudibranch *Phyllidia coelestis*

Marianna Carbone [1,*], M. Letizia Ciavatta [1], Emiliano Manzo [1], Xiao-Lu Li [2], Ernesto Mollo [1], I. Wayan Mudianta [3], Yue-Wei Guo [2,*] and Margherita Gavagnin [1,*]

[1] *Consiglio Nazionale delle Ricerche (CNR), Istituto di Chimica Biomolecolare (ICB), Via Campi Flegrei, 34, 80078 Pozzuoli (Na), Italy; lciavatta@icb.cnr.it (M.L.C); emanzo@icb.cnr.it (E.Manzo); emollo@icb.cnr.it (E.Mollo)*

[2] *State Key Laboratory of Drug Research, Shanghai Institute of Materia Medica, Chinese Academy of Sciences, Zuchongzhi Road 555 Zhangjiang Hi-Tech Park, Shanghai 201203, China; lululi666666@126.com (X.-L.L.)*

[3] *Study Program of Chemical Analysis, Universitas Pendidikan Ganesha, Bali, 81116, Indonesia; mudianta@undiksha.ac.id (I.W.M.)*

Content

S1 - ^{1}H NMR spectrum of biflora-4,9,15-triene (**1**) ($CDCl_3$, Bruker 400 MHz).

S2 - ^{1}H-^{1}H COSY spectrum of biflora-4,9,15-triene (**1**) ($CDCl_3$, Bruker 400 MHz).

S3 - HSQC spectrum of biflora-4,9,15-triene (**1**) ($CDCl_3$, Bruker 400 MHz).

S4 - HMBC spectrum of biflora-4,9,15-triene (**1**) ($CDCl_3$, Bruker 400 MHz, *J*=7Hz).

S5 - ^{1}H NMR spectrum of 7-isocyano-11-cycloamphilectene (**2**) ($CDCl_3$, Bruker 400 MHz).

S6 - ^{1}H-^{1}H COSY spectrum of 7-isocyano-11-cycloamphilectene (**2**) ($CDCl_3$, Bruker 400 MHz).

S7 - HSQC spectrum of 7-isocyano-11-cycloamphilectene (**2**) ($CDCl_3$, Bruker 400 MHz).

S8 - HMBC spectrum of 7-isocyano-11-cycloamphilectene (**2**) ($CDCl_3$, Bruker 400 MHz, *J*=7Hz).

S9 - ^{1}H NMR spectrum of 8,15-diisocyano-11(20)-amphilectene (**3**) ($CDCl_3$, Bruker 300 MHz).

S10 - ^{13}C NMR spectrum of 8,15-diisocyano-11(20)-amphilectene (**3**) ($CDCl_3$, Bruker 300 MHz).

S11 - ^{1}H NMR spectrum of 7-isocyanoamphilecta-11(20),15-diene (**4**) ($CDCl_3$, Bruker 400 MHz).

S12 - ^{1}H-^{1}H COSY spectrum of 7-isocyanoamphilecta-11(20),15-diene (**4**) ($CDCl_3$, Bruker 400 MHz).

S13 - HSQC spectrum of 7-isocyanoamphilecta-11(20),15-diene (**4**) ($CDCl_3$, Bruker 400 MHz).

S14 - HMBC spectrum of 7-isocyanoamphilecta-11(20),15-diene (**4**) ($CDCl_3$, Bruker 400 MHz, *J*=7Hz).

S15 - ^{1}H NMR spectrum of 7-isocyanoamphilecta-10,14-diene (**5**) ($CDCl_3$, Bruker 400 MHz).

S16 - ^{13}C NMR spectrum of 7-isocyanoamphilecta-10,14-diene (**5**) ($CDCl_3$, Bruker 300 MHz).

S17 - ^{1}H-^{1}H COSY spectrum of 7-isocyanoamphilecta-10,14-diene (**5**) ($CDCl_3$, Bruker 400 MHz).

S18 - HSQC spectrum of 7-isocyanoamphilecta-10,14-diene (**5**) ($CDCl_3$, Bruker 400 MHz).

S19 - HMBC spectrum of 7-isocyanoamphilecta-10,14-diene (**5**) ($CDCl_3$, Bruker 500 MHz, *J*=7Hz).

S20 - ^{1}H NMR spectrum of 7-isocyanoamphilecta-10,14-diene (**5**) (C_6D_6, Bruker 400 MHz).

S21- ^{1}H-^{1}H COSY spectrum of 7-isocyanoamphilecta-10,14-diene (**5**) (C_6D_6, Bruker 400 MHz).

S22 - HSQC spectrum of 7-isocyanoamphilecta-10,14-diene (**5**) (C_6D_6, Bruker 400 MHz).

S23 - ^{1}H NMR spectrum of 7-isocyanoamphilecta-1,14-diene (**6**) ($CDCl_3$, Bruker 500 MHz).

S24 - ^{13}C NMR spectrum of 7-isocyanoamphilecta-1,14-diene (**6**) ($CDCl_3$, Bruker 300 MHz).

S25 - DEPT spectrum of 7-isocyanoamphilecta-1,14-diene (**6**) ($CDCl_3$, Bruker 300 MHz).

S26 - ^1H-^1H COSY spectrum of 7-isocyanoamphilecta-1,14-diene (**6**) ($CDCl_3$, Bruker 500 MHz).

S27 - HSQC spectrum of 7-isocyanoamphilecta-1,14-diene (**6**) ($CDCl_3$, Bruker 500 MHz).

S28 - HMBC spectrum of 7-isocyanoamphilecta-1,14-diene (**6**) ($CDCl_3$, Bruker 500 MHz, *J*=7Hz).

S29 - ^1H NMR spectrum of 7-isocyanoamphilecta-1,14-diene (**6**) (C_6D_6, Bruker 500 MHz).

S30 - ^1H-^1H COSY spectrum of 7-isocyanoamphilecta-1,14-diene (**6**) (C_6D_6, Bruker 500 MHz).

S31 - HSQC spectrum of 7-isocyanoamphilecta-1,14-diene (**6**) (C_6D_6, Bruker 500 MHz).

S32 - HMBC spectrum of 7-isocyanoamphilecta-1,14-diene (**6**) (C_6D_6, Bruker 500 MHz, *J*=7Hz).

S33 - ^1H NMR spectrum of 7-isocyanoamphilecta-11(20),14-diene (**7**) ($CDCl_3$, Bruker 300 MHz).

S34 - ^{13}C NMR spectrum of 7-isocyanoamphilecta-11(20),14-diene (**7**) ($CDCl_3$, Bruker 300 MHz).

S35 - ^1H-^1H COSY spectrum of 7-isocyanoamphilecta-11(20),14-diene (**7**) ($CDCl_3$, Bruker 500 MHz).

S36 - HSQC spectrum of 7-isocyanoamphilecta-11(20),14-diene (**7**) ($CDCl_3$, Bruker 500 MHz).

S37 - HMBC spectrum of 7-isocyanoamphilecta-11(20),14-diene (**7**) ($CDCl_3$, Bruker 500 MHz, *J*=7Hz).

S38 - ^1H NMR spectrum of 7-formamidoamphilecta-11(20),15-diene (**8**) ($CDCl_3$, Bruker 600 MHz).

S39 - ^{13}C NMR spectrum of 7-formamidoamphilecta-11(20),15-diene (**8**) ($CDCl_3$, Bruker 400 MHz).

S40 - ^1H-^1H COSY spectrum of 7-formamidoamphilecta-11(20),15-diene (**8**) ($CDCl_3$, Bruker 600 MHz).

S41 - HSQC spectrum of 7-formamidoamphilecta-11(20),15-diene (**8**) ($CDCl_3$, Bruker 600 MHz).

S42 - HMBC spectrum of 7-formamidoamphilecta-11(20),15-diene (**8**) ($CDCl_3$, Bruker 600 MHz, *J*=7Hz).

S43 - ^1H NMR spectrum of 7-formamido amphilecta-10,14-diene (**9**) ($CDCl_3$, Bruker 400 MHz).

S44 - ^1H-^1H COSY spectrum of 7-formamido amphilecta-10,14-diene (**9**) ($CDCl_3$, Bruker 400 MHz).

S45 - HSQC spectrum of 7-formamido amphilecta-10,14-diene (**9**) ($CDCl_3$, Bruker 400 MHz).

S46 - HMBC spectrum of 7-formamido amphilecta-10,14-diene (**9**) ($CDCl_3$, Bruker 400 MHz, *J*=7Hz).

S47 - ^1H NMR spectrum of 7-formamido amphilecta-10,14-diene (**9**) (C_6D_6, Bruker 400 MHz).

S48 - ^1H-^1H COSY spectrum of 7-formamido amphilecta-10,14-diene (**9**) (C_6D_6, Bruker 400 MHz).

S49 - HSQC spectrum of 7-formamido amphilecta-10,14-diene (**9**) (C_6D_6, Bruker 400 MHz).

S50 - HMBC spectrum of 7-formamido amphilecta-10,14-diene (**9**) (C_6D_6, Bruker 400 MHz, *J*=7Hz).

S51 - ^{1}H NMR spectrum of 8-isocyano-15-formamido-11(20)-amphilectene (**10**) ($CDCl_3$, Bruker 400 MHz).

S52 - ^{1}H-^{1}H COSY spectrum of 8-isocyano-15-formamido-11(20)-amphilectene (**10**) ($CDCl_3$, Bruker 400 MHz).

S53 - HSQC spectrum of 8-isocyano-15-formamido-11(20)-amphilectene (**10**) ($CDCl_3$, Bruker 400 MHz).

S54 - Figure 1. Individuals of *P. coelestis* collected in Hainan (China) in 2002 (a) and 2013 (b).

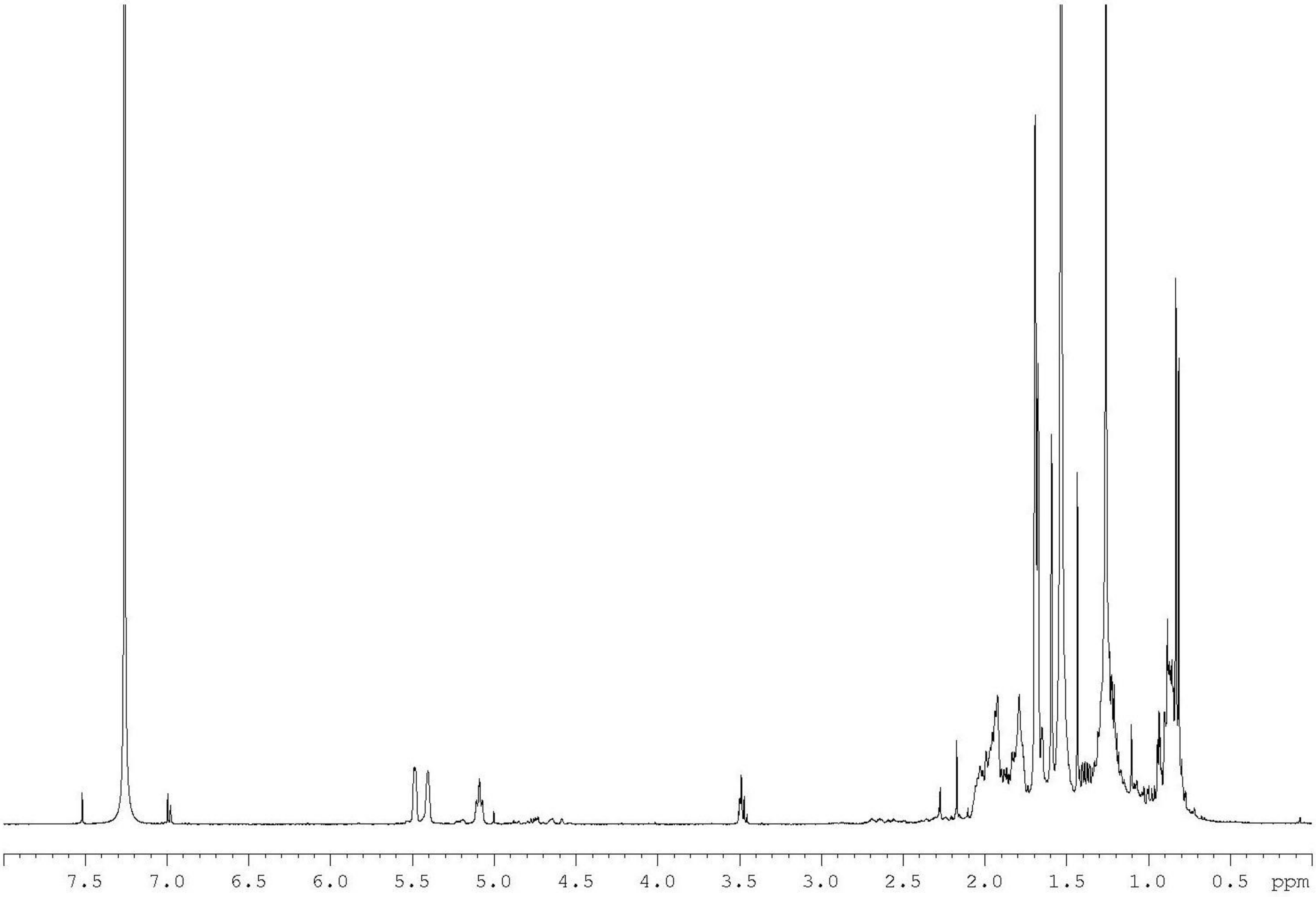

^{1}H NMR spectrum of biflora-4,9,15-triene (**1**) ($CDCl_3$, Bruker 400 MHz).

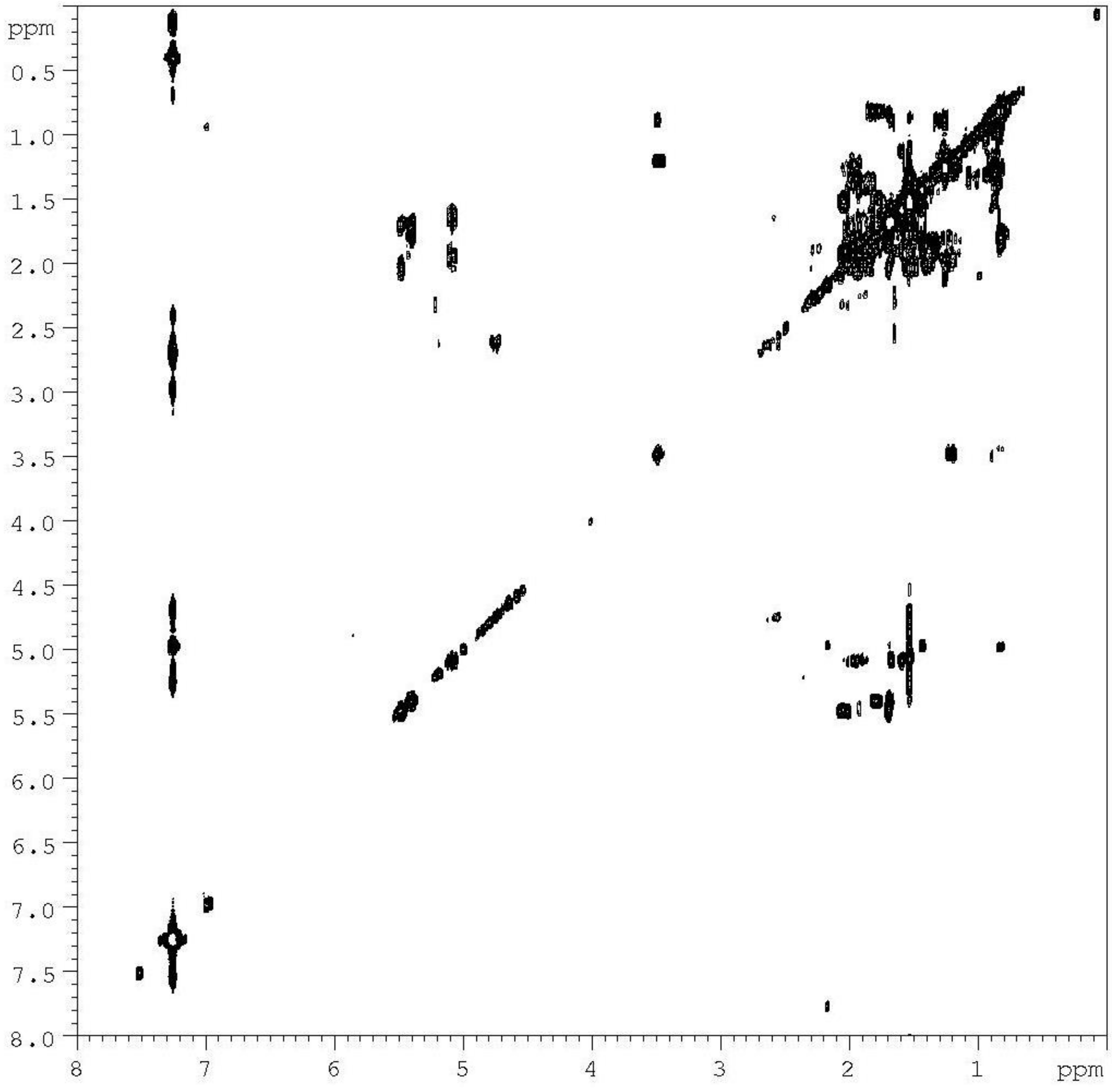

^{1}H-^{1}H COSY spectrum of biflora-4,9,15-triene (**1**) ($CDCl_3$, Bruker 400 MHz).

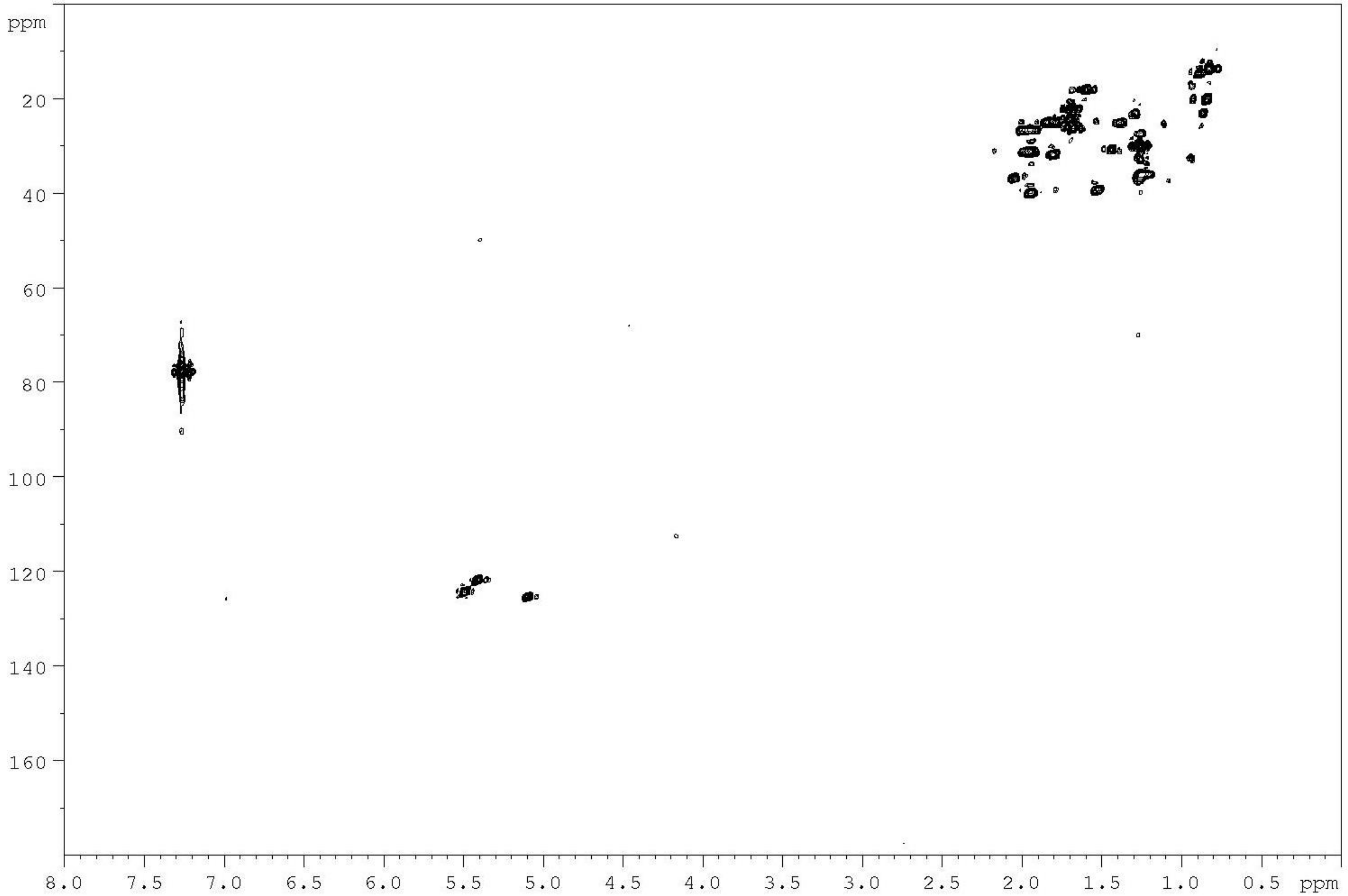

HSQC spectrum of biflora-4,9,15-triene (**1**) ($CDCl_3$, Bruker 400 MHz).

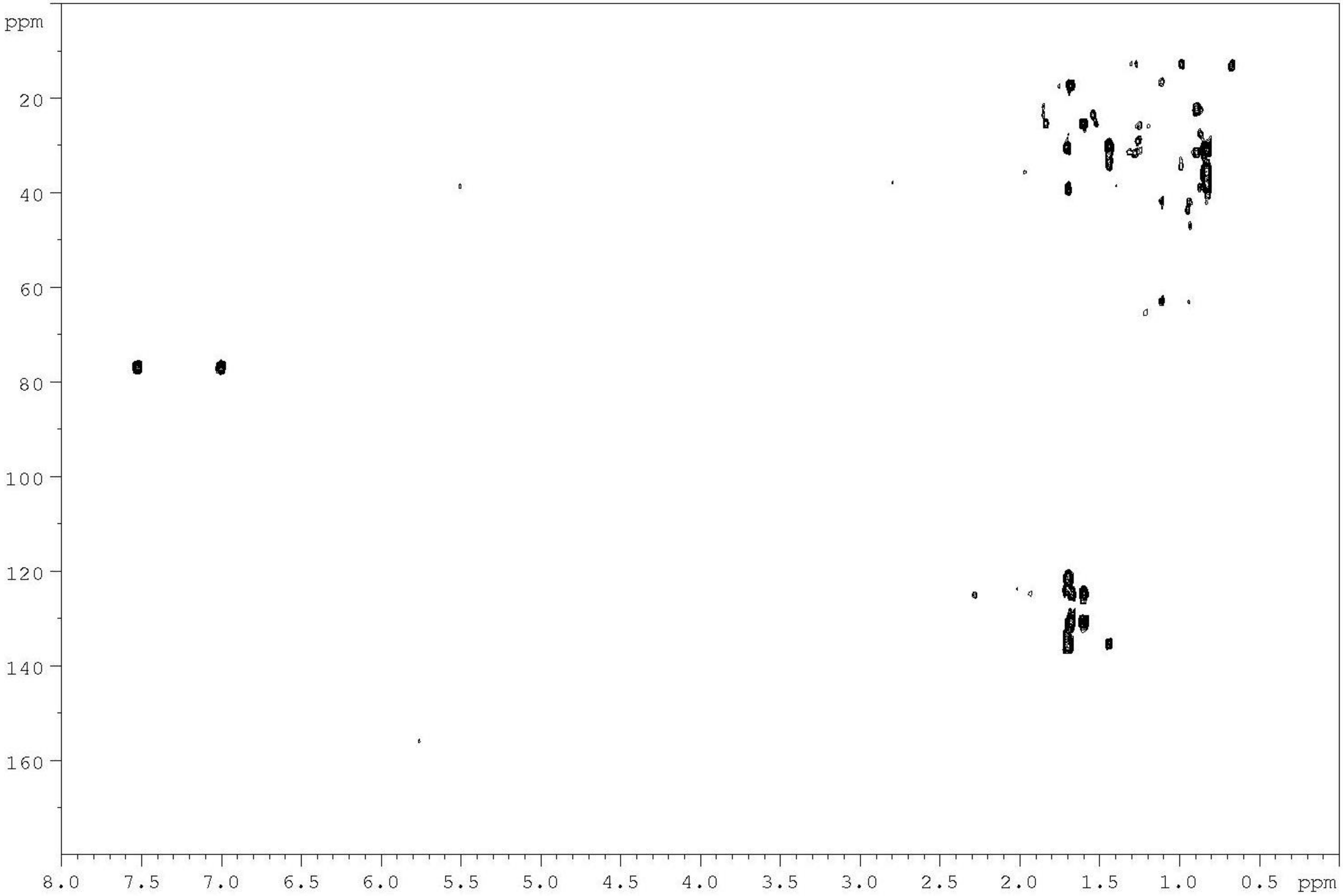

HMBC spectrum of biflora-4,9,15-triene (**1**) ($CDCl_3$, Bruker 400 MHz, *J*=7Hz).

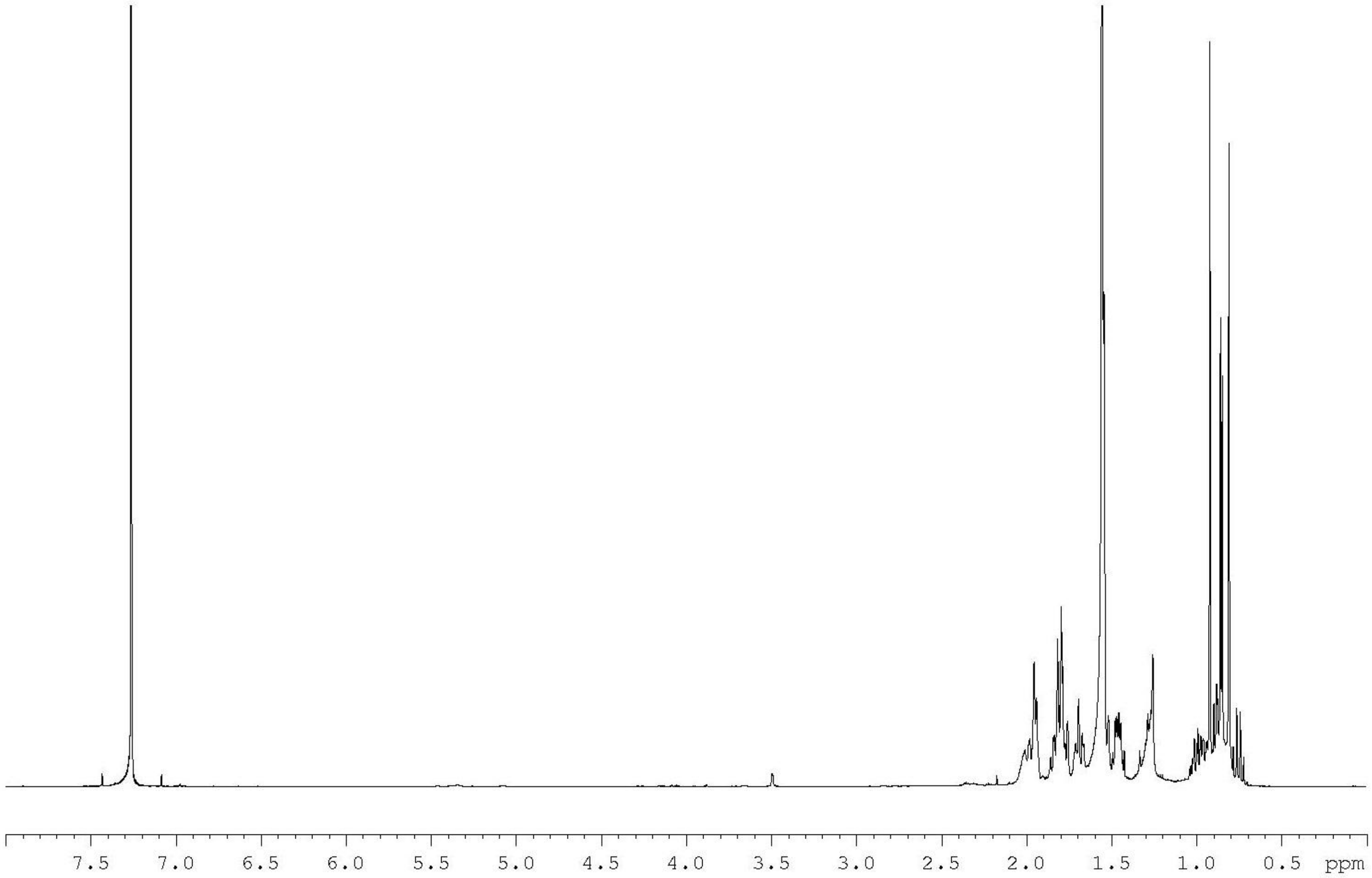

^{1}H NMR spectrum of 7-isocyano-11-cycloamphilectene (**2**) ($CDCl_3$, Bruker 400 MHz).

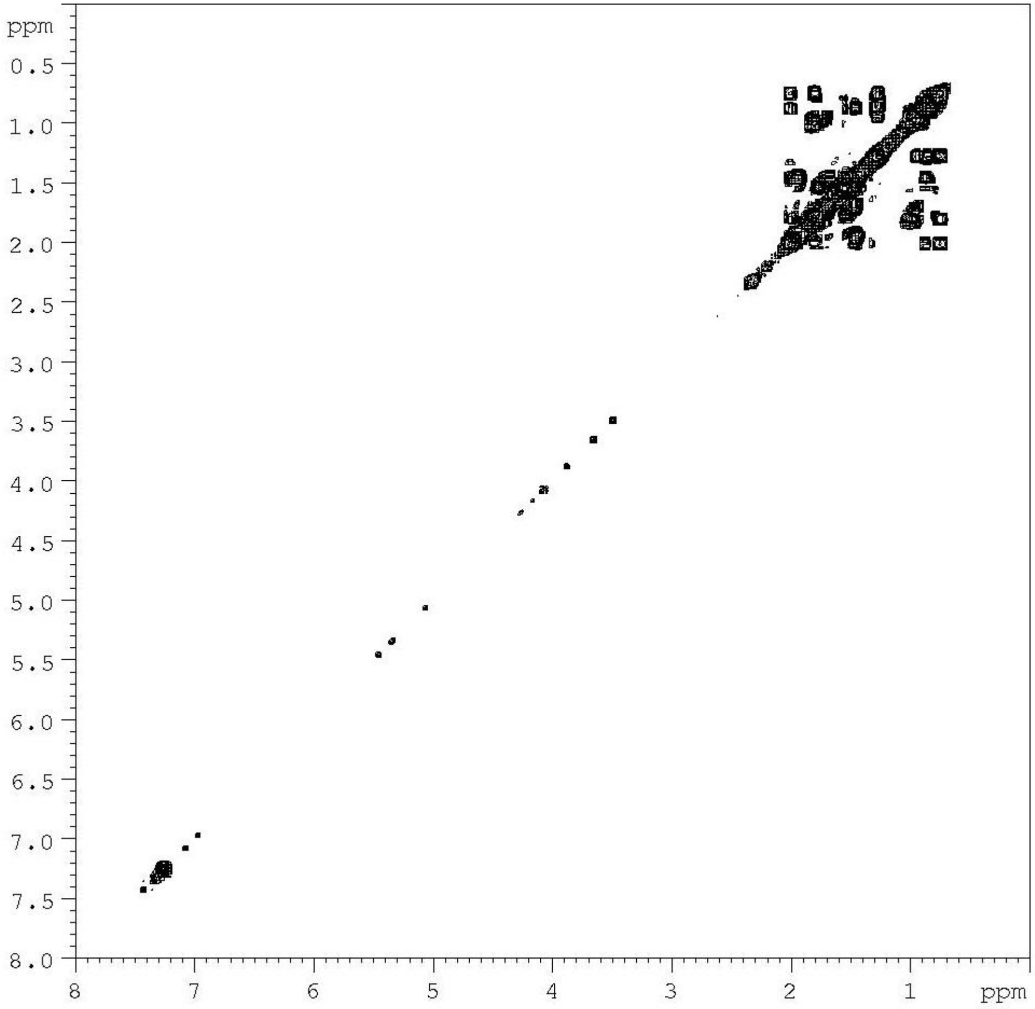

^{1}H-^{1}H COSY spectrum of 7-isocyano-11-cycloamphilectene (**2**) ($CDCl_3$, Bruker 400 MHz).

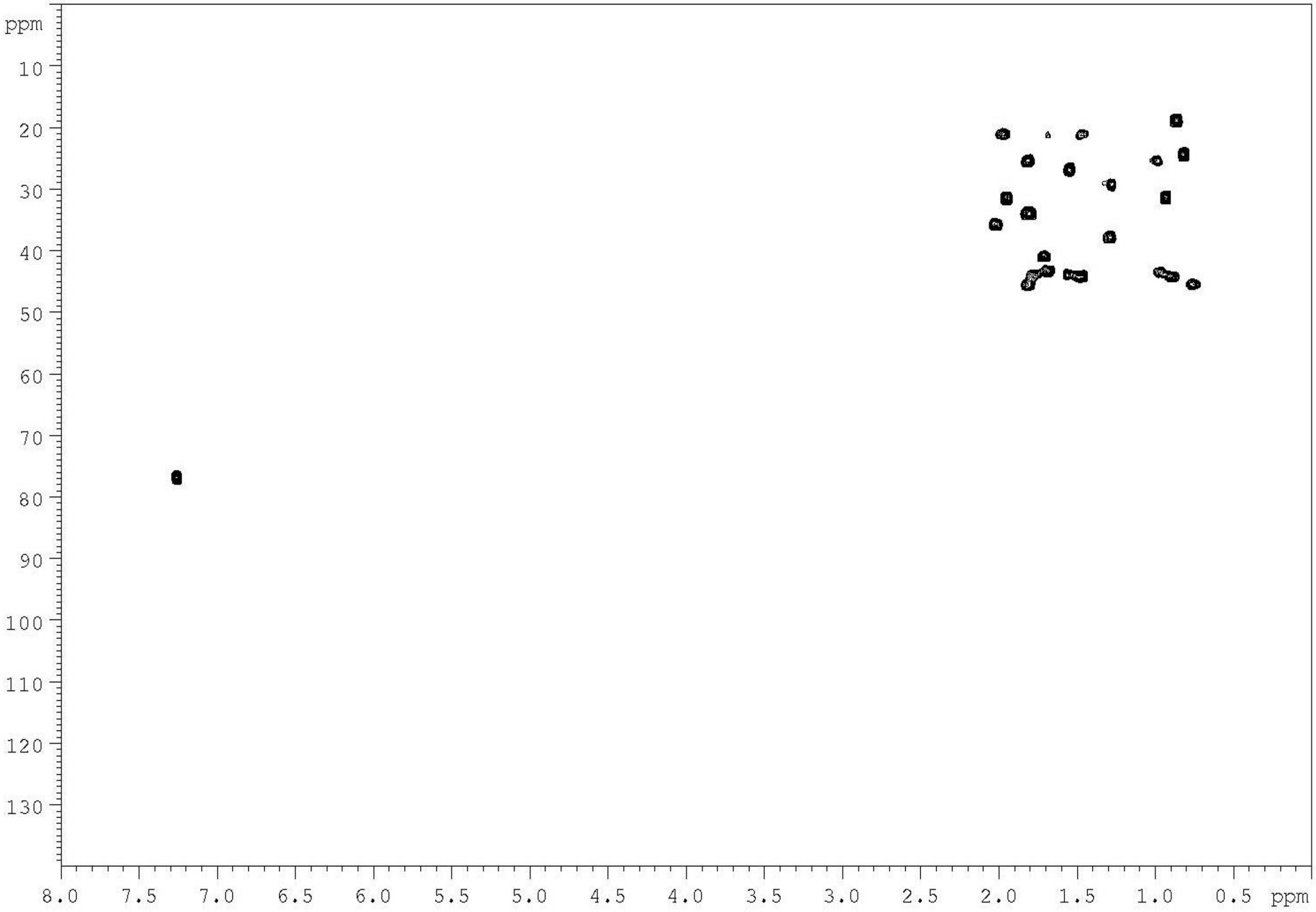

HSQC spectrum of 7-isocyano-11-cycloamphilectene (**2**) ($CDCl_3$, Bruker 400 MHz).

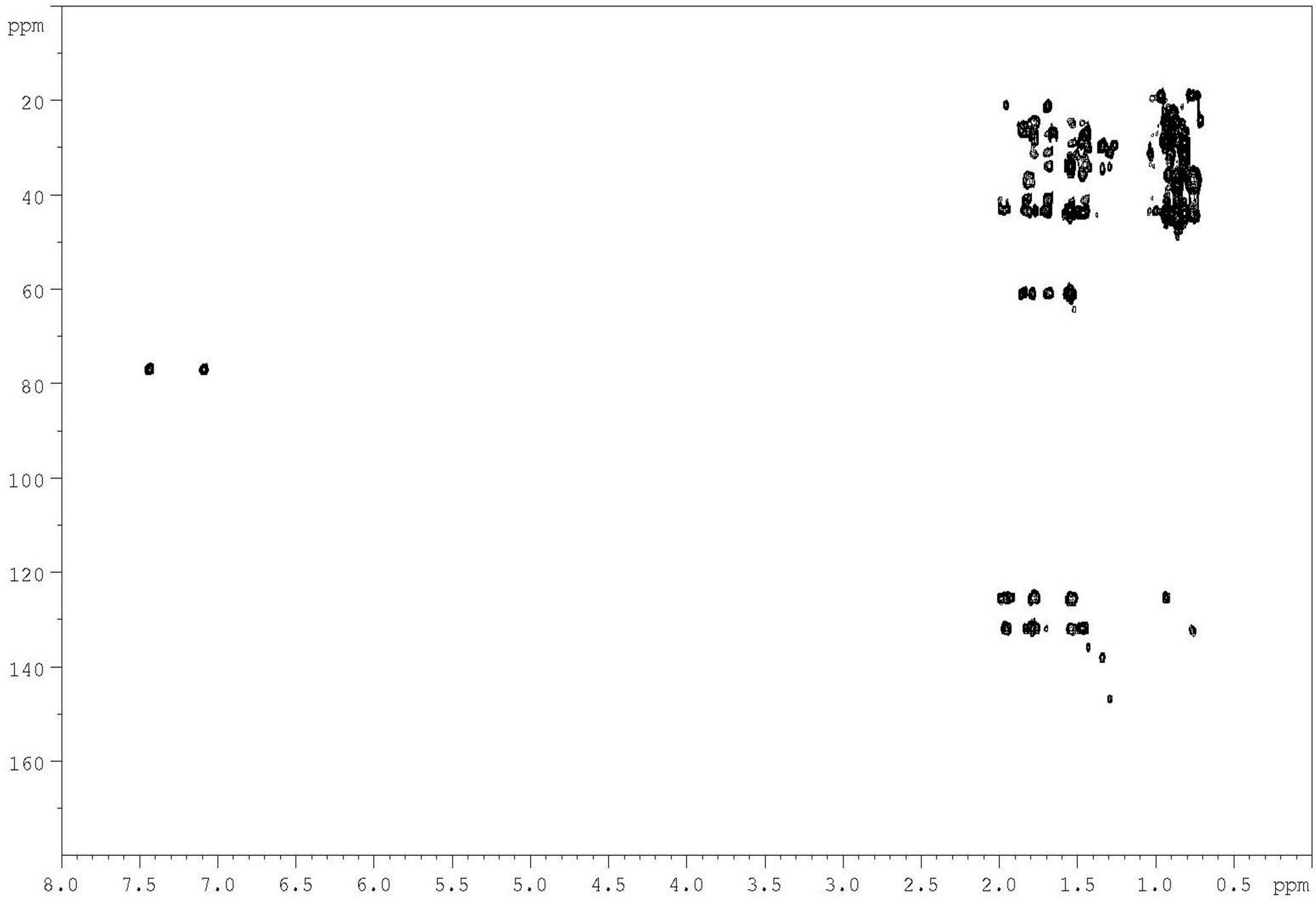

HMBC spectrum of 7-isocyano-11-cycloamphilectene (**2**) ($CDCl_3$, Bruker 400 MHz, *J*=7Hz)

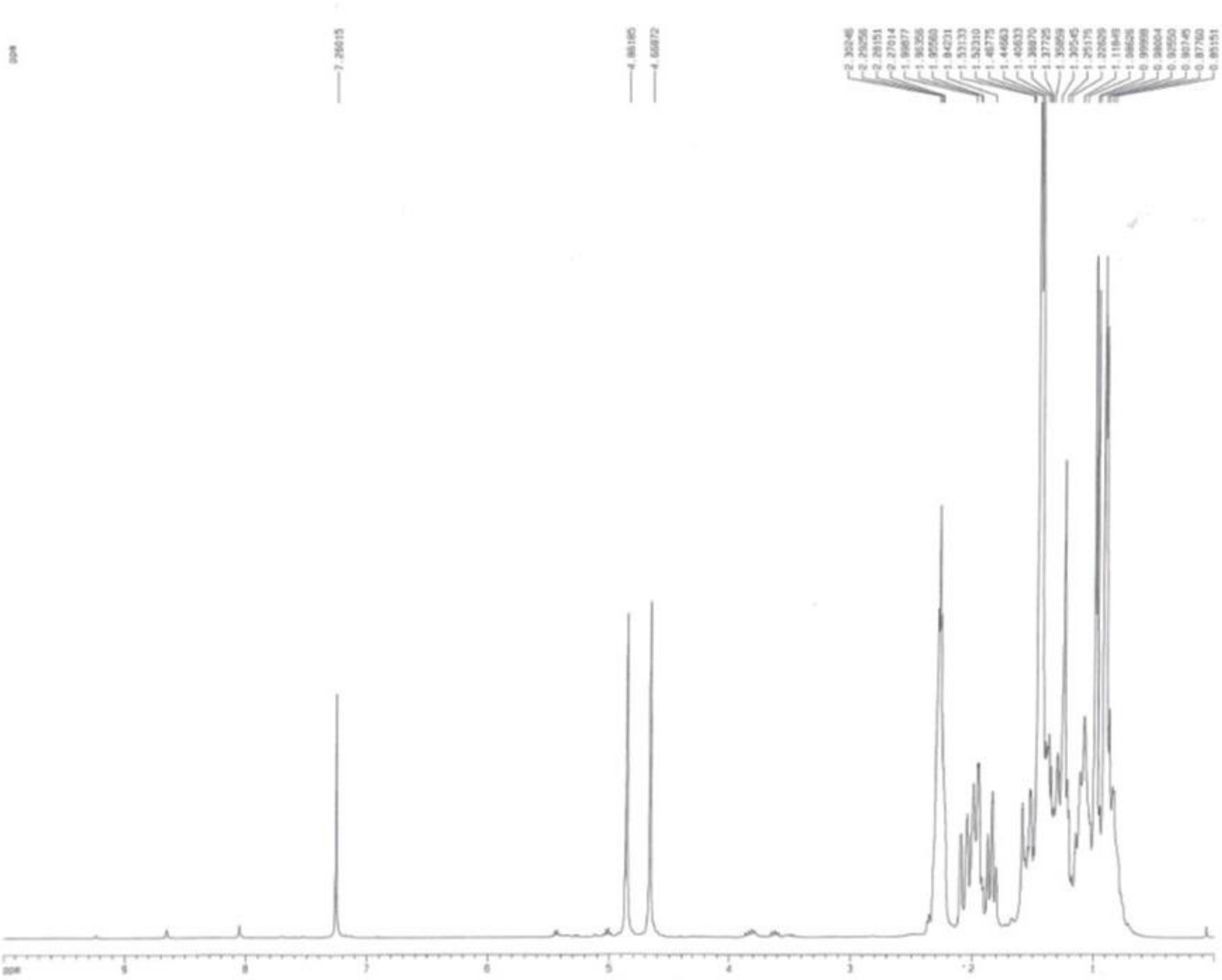

^{1}H NMR spectrum of 8,15-diisocyano-11(20)-amphilectene (**3**) ($CDCl_3$, Bruker 300 MHz).

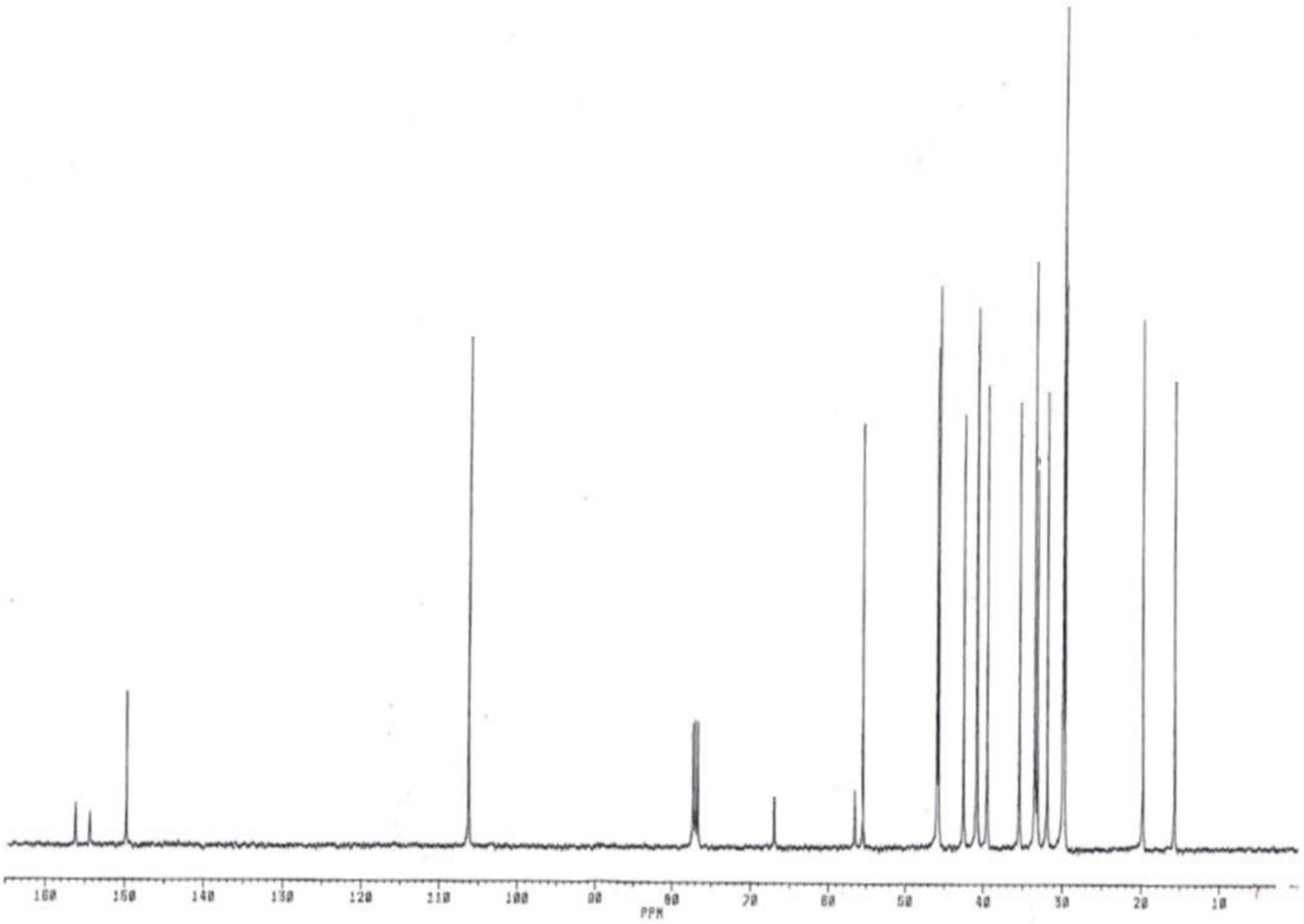

^{13}C NMR spectrum of 8,15-diisocyano-11(20)-amphilectene (**3**) ($CDCl_3$, Bruker 300 MHz).

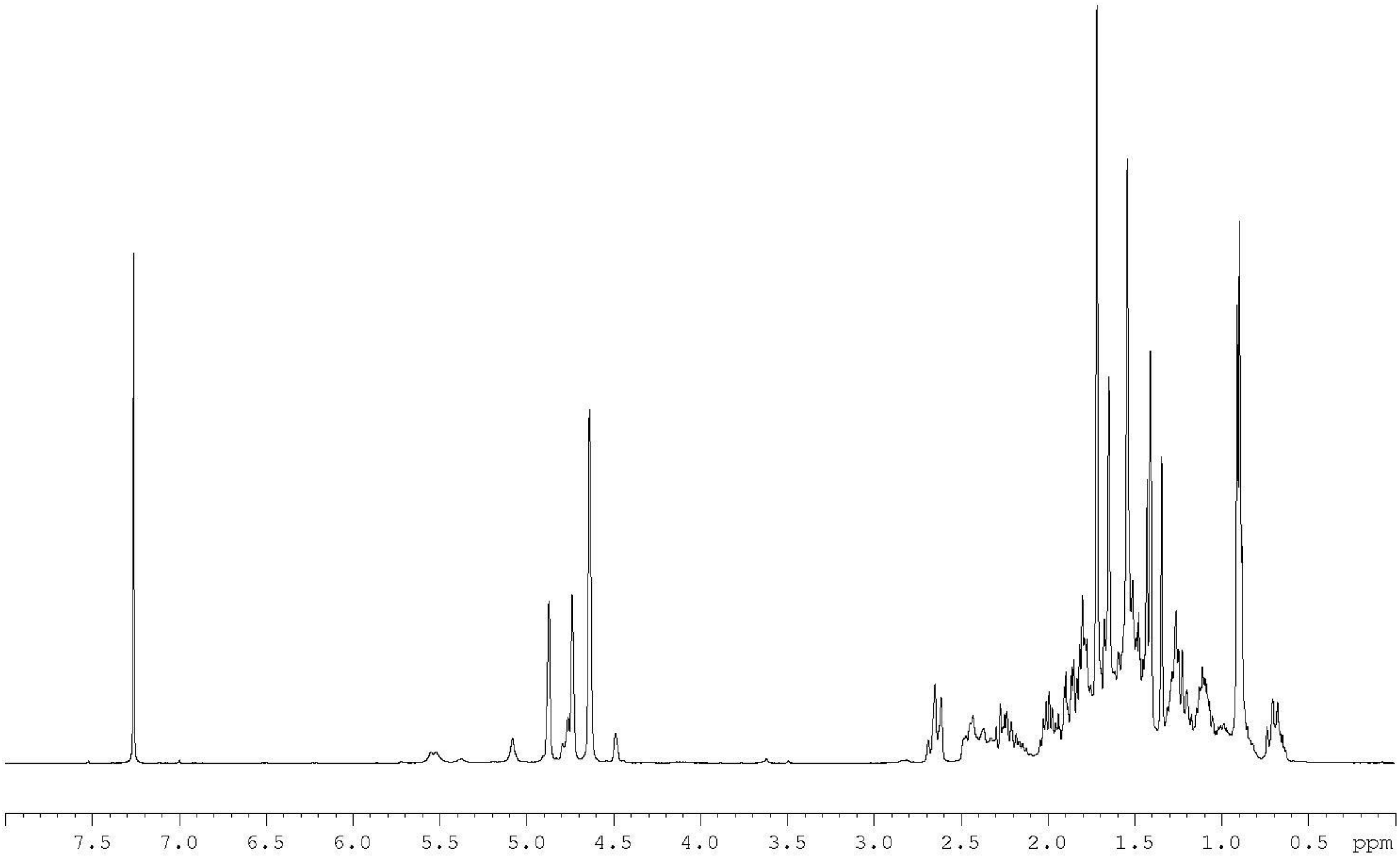

^{1}H NMR spectrum of 7-isocyanoamphilecta-11(20),15-diene (**4**) ($CDCl_3$, Bruker 400 MHz).

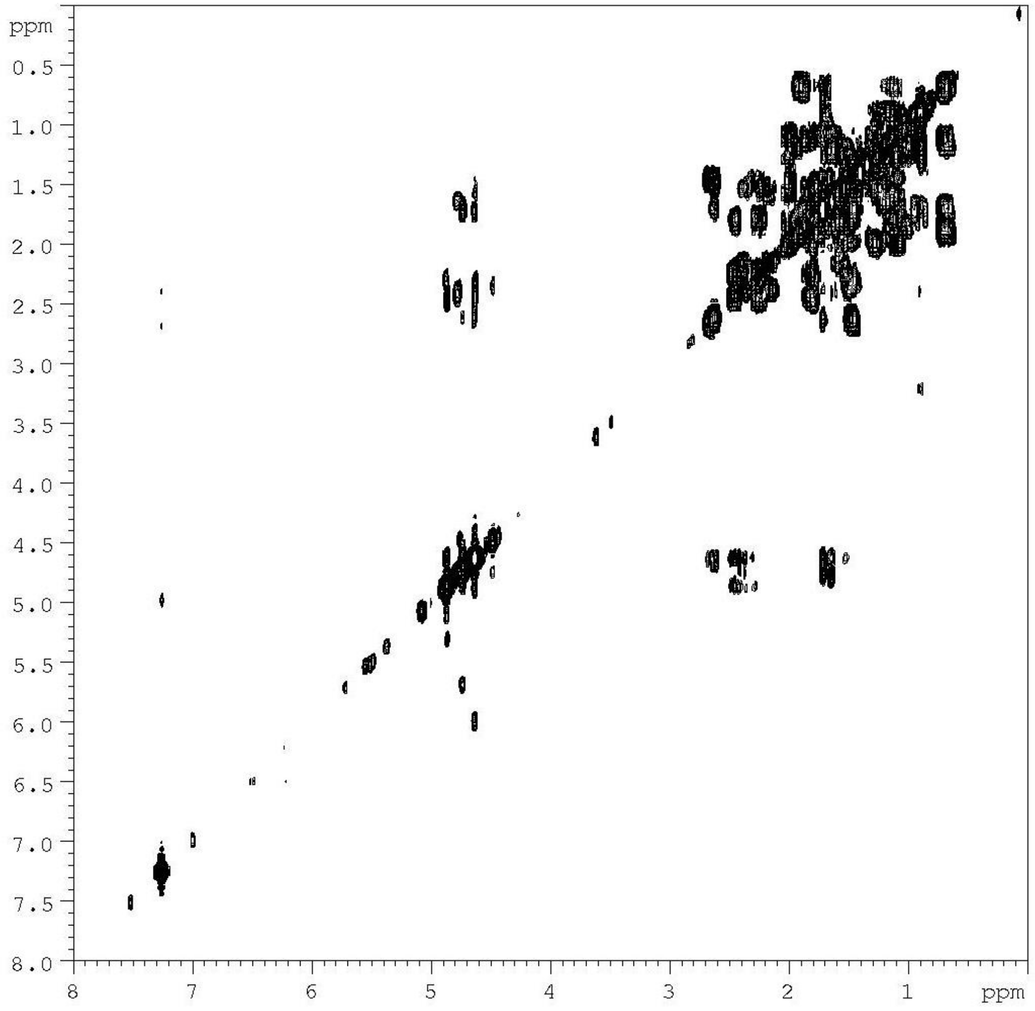

^{1}H-^{1}H COSY spectrum of 7-isocyanoamphilecta-11(20),15-diene (**4**) ($CDCl_3$, Bruker 400 MHz).

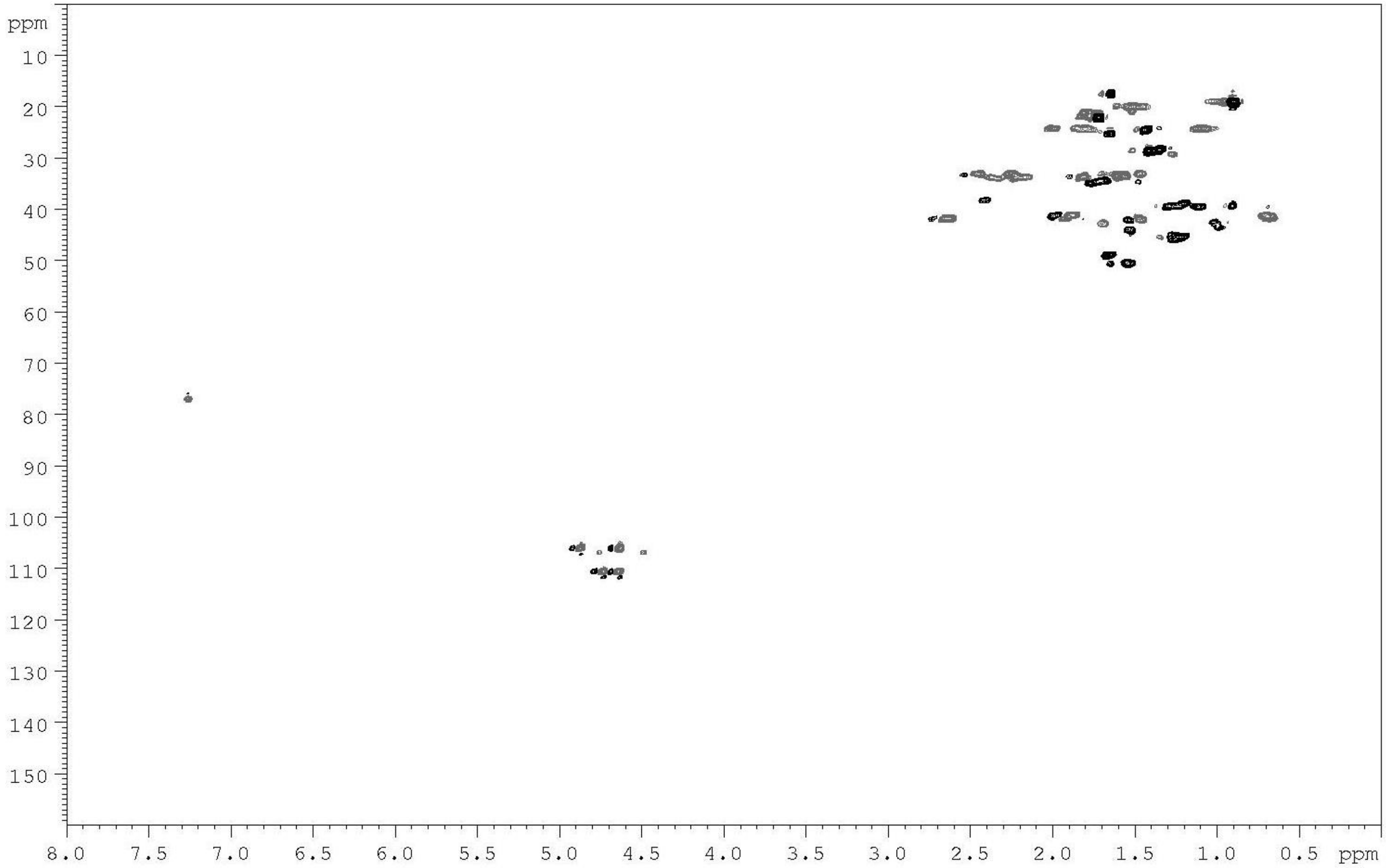

HSQC spectrum of 7-isocyanoamphilecta-11(20),15-diene (**4**) ($CDCl_3$, Bruker 400 MHz).

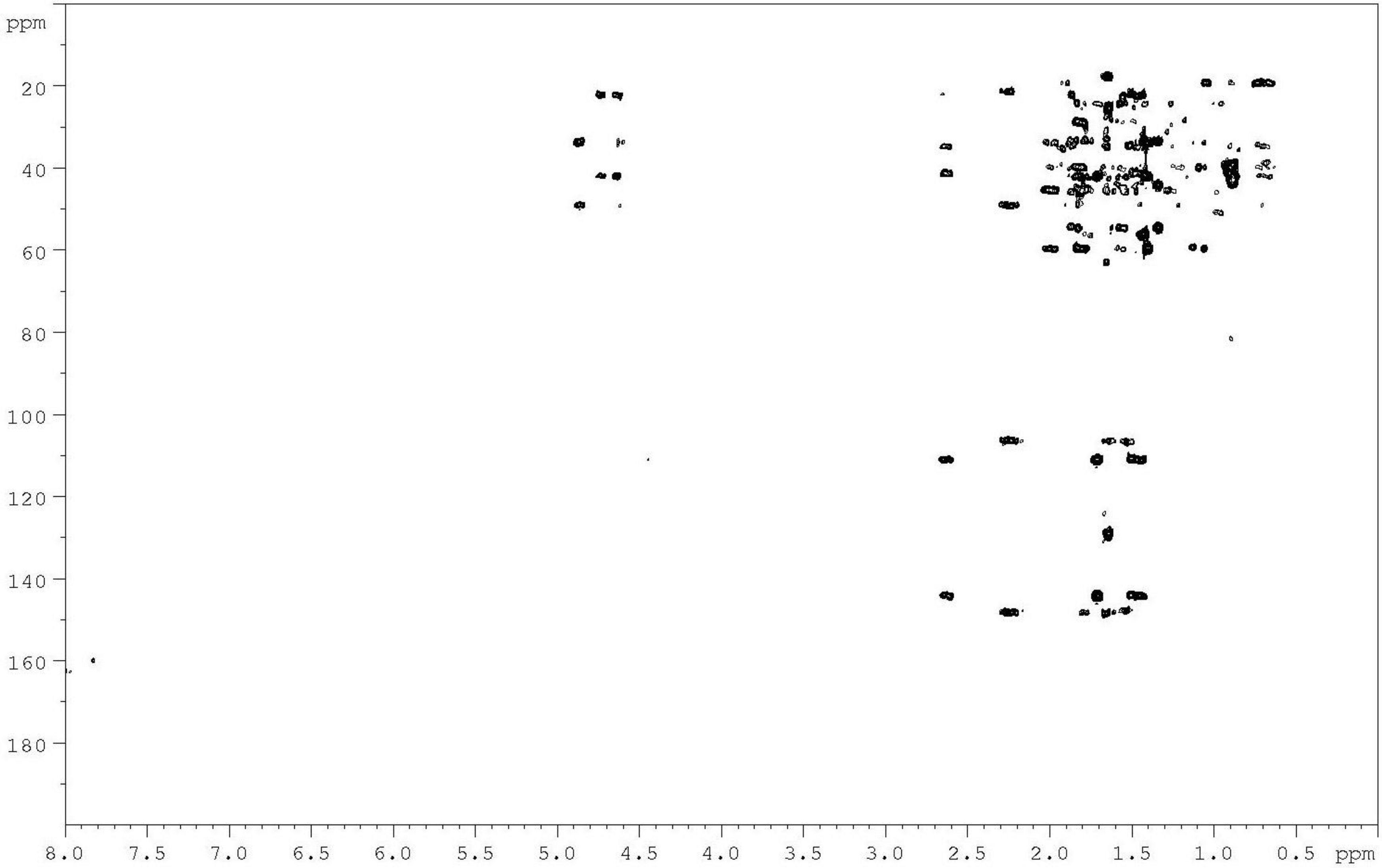

HMBC spectrum of 7-isocyanoamphilecta-11(20),15-diene (**4**) ($CDCl_3$, Bruker 400 MHz, *J*=7Hz).

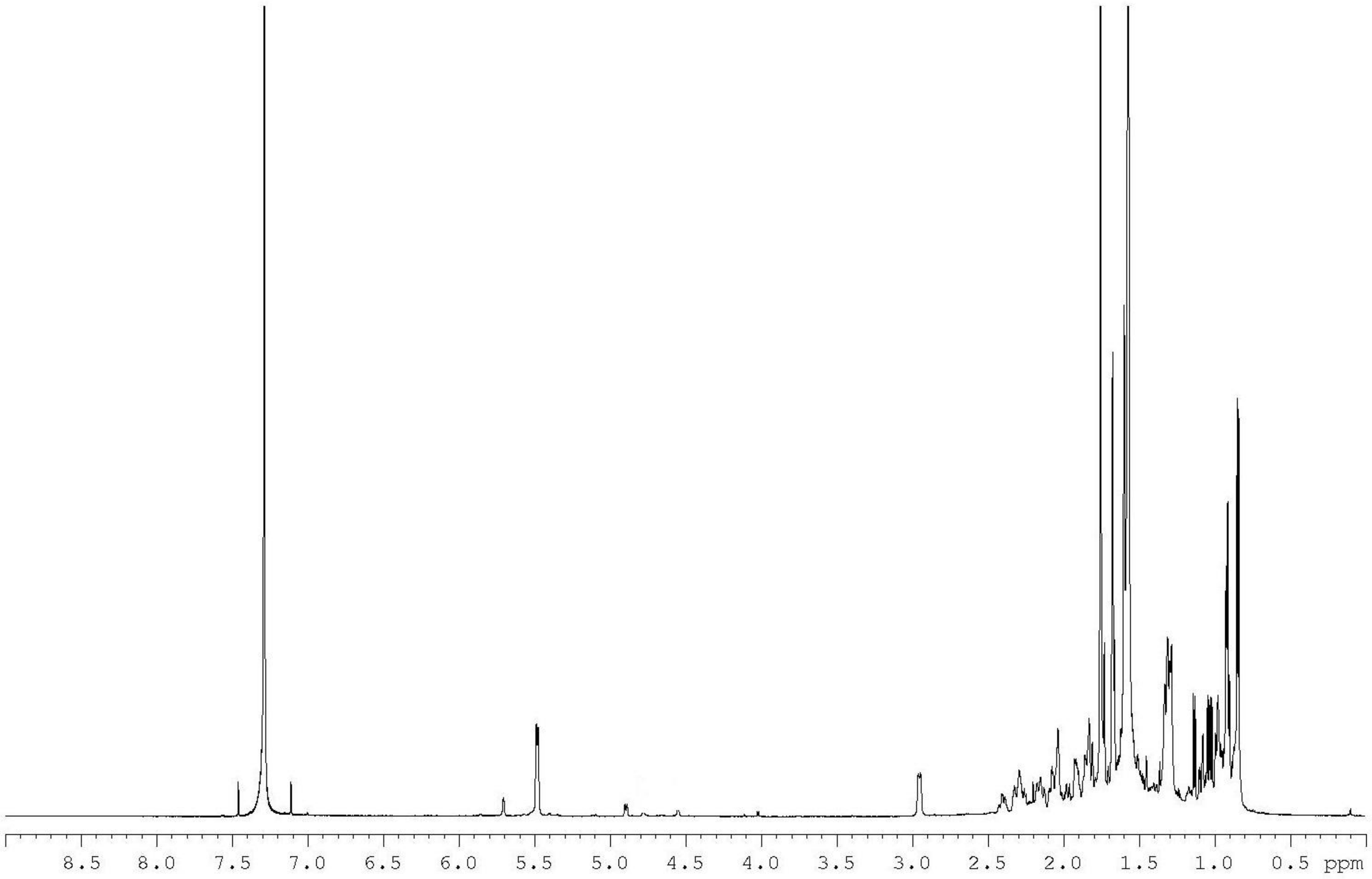

^{1}H NMR spectrum of 7-isocyanoamphilecta-10,14-diene (**5**) ($CDCl_3$, Bruker 400 MHz).

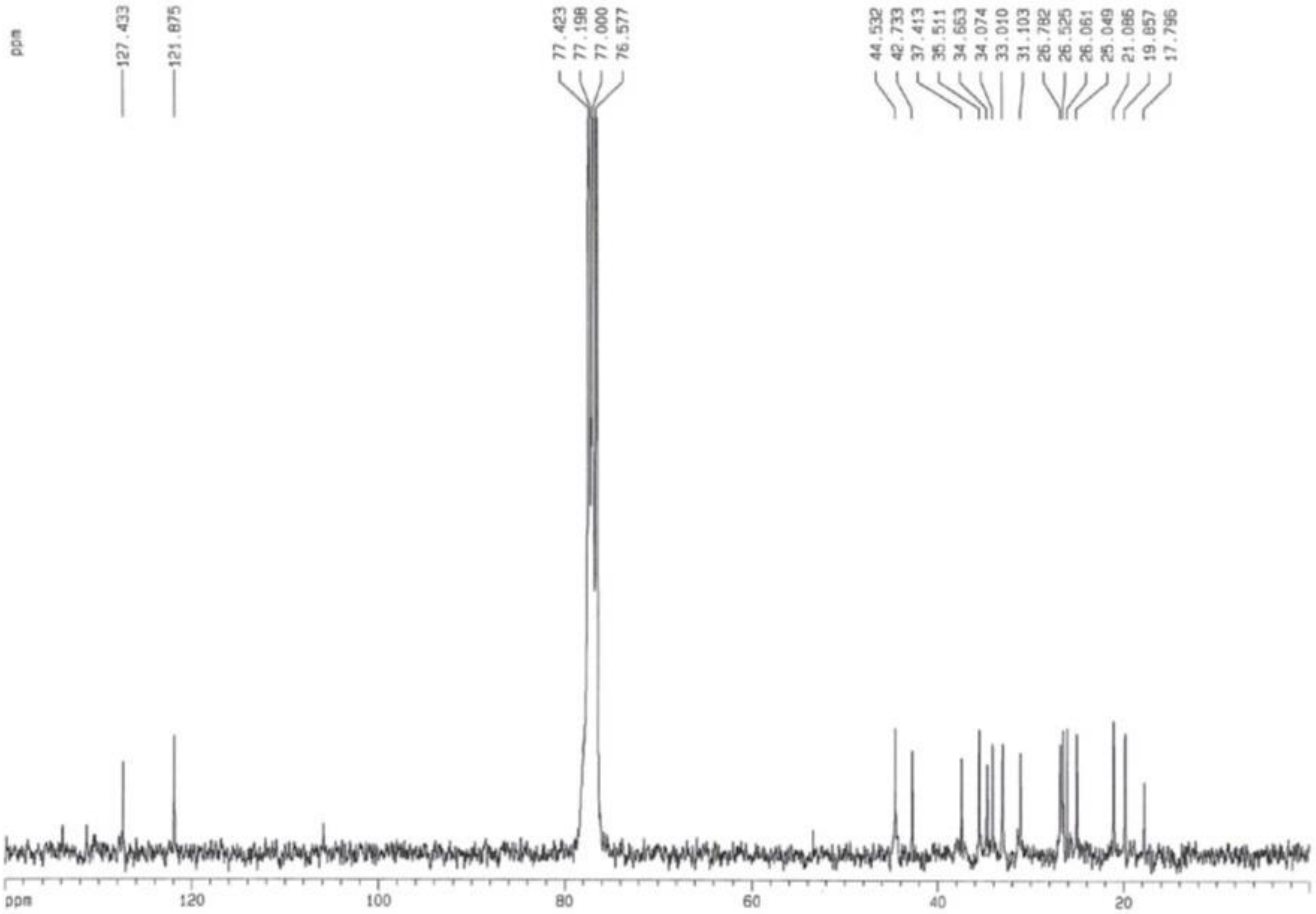

^{13}C NMR spectrum of 7-isocyanoamphilecta-10,14-diene (**5**) ($CDCl_3$, Bruker 300 MHz).

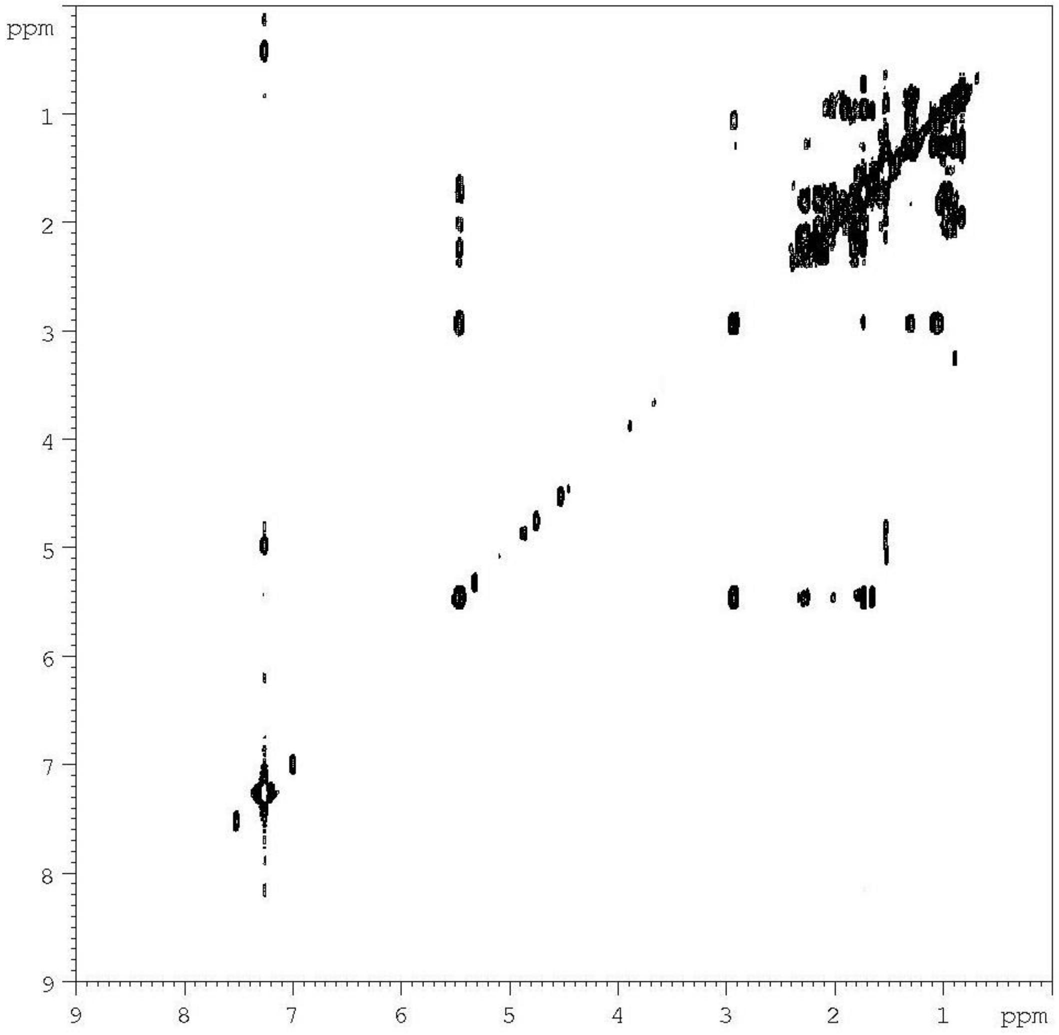

^{1}H-^{1}H COSY spectrum of 7-isocyanoamphilecta-10,14-diene (**5**) ($CDCl_3$, Bruker 400 MHz).

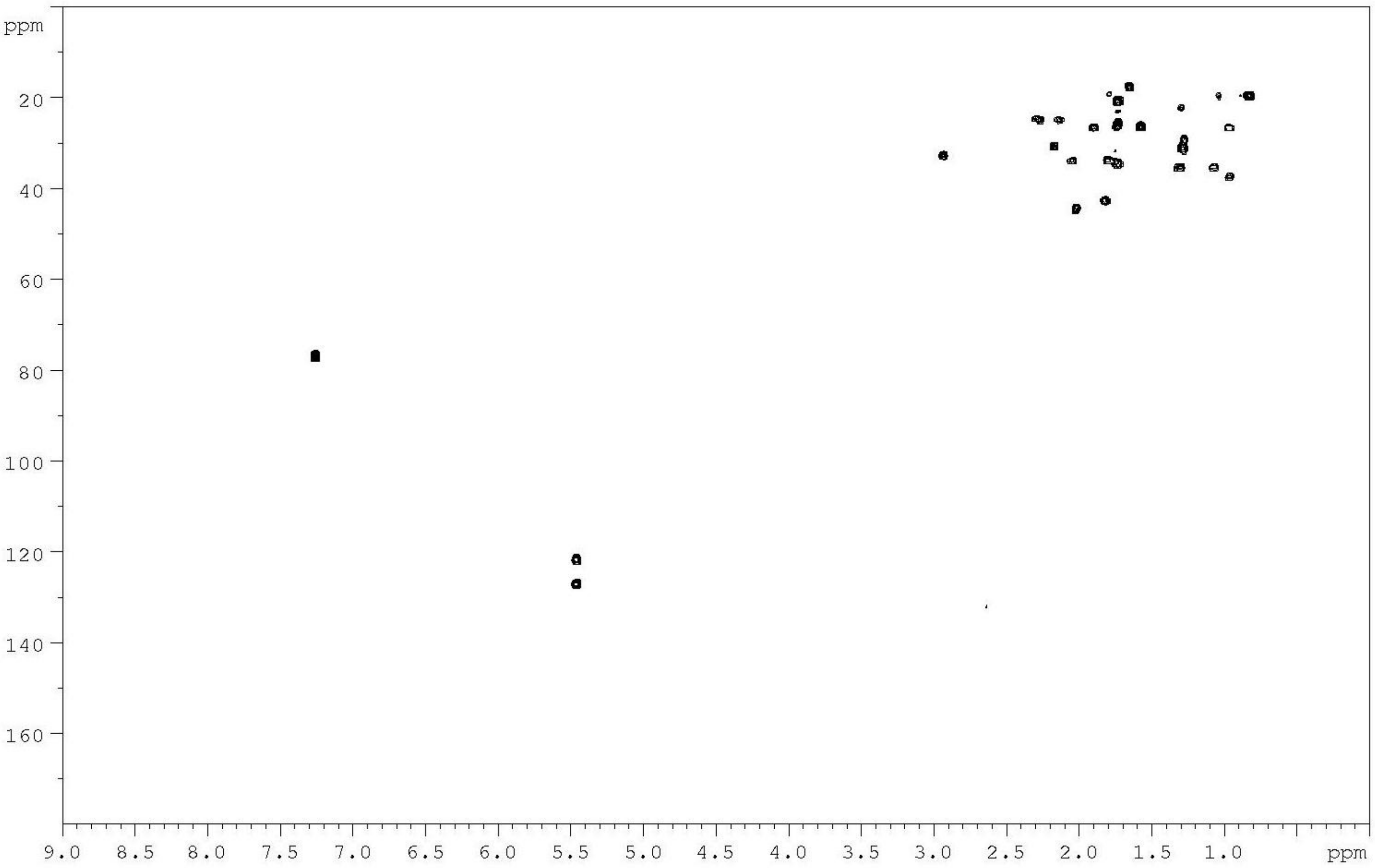

HSQC spectrum of 7-isocyanoamphilecta-10,14-diene (**5**) ($CDCl_3$, Bruker 400 MHz).

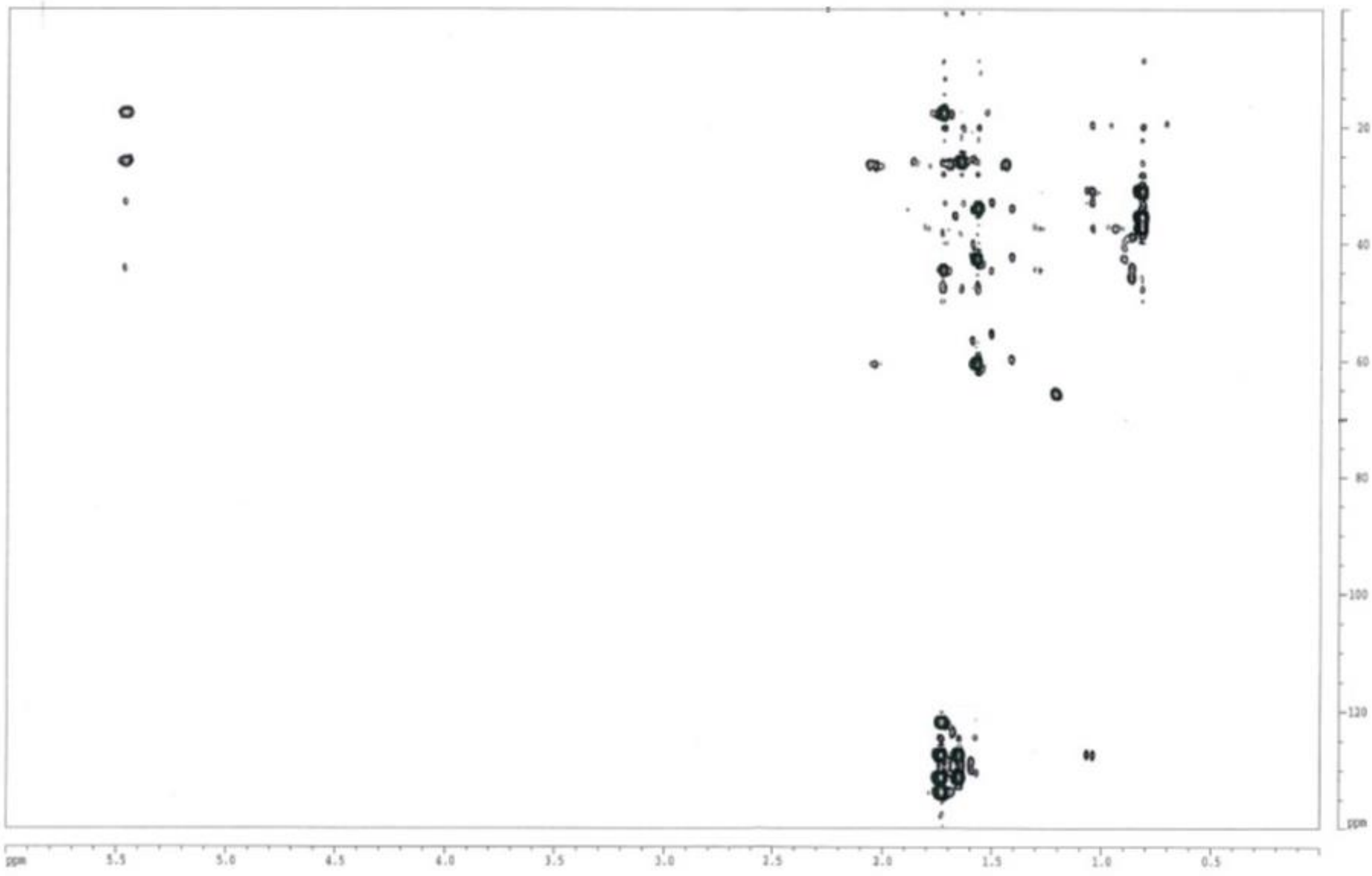

HMBC spectrum of 7-isocyanoamphilecta-10,14-diene (**5**) ($CDCl_3$, Bruker 500 MHz, *J*=7Hz).

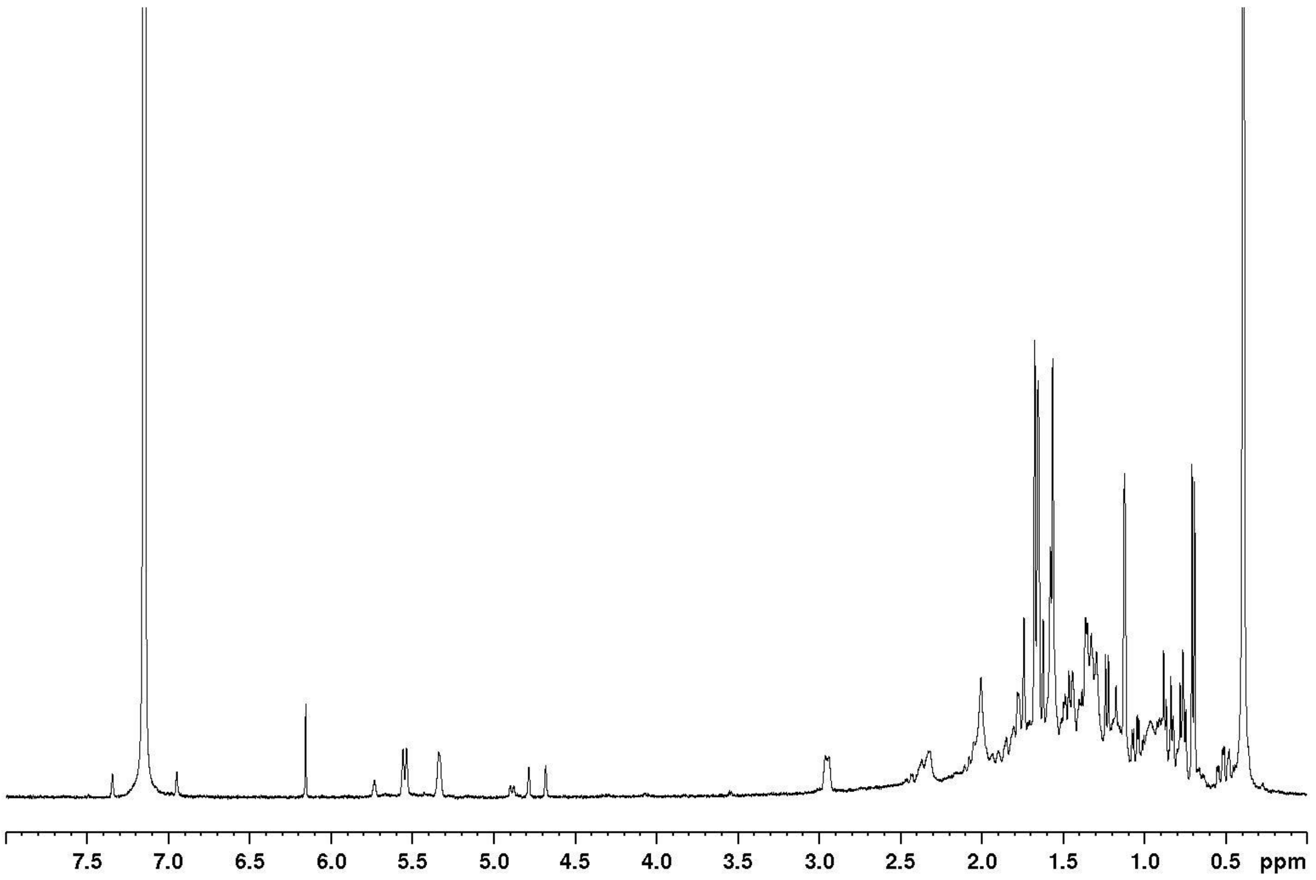

^{1}H NMR spectrum of 7-isocyanoamphilecta-10,14-diene (**5**) (C_6D_6, Bruker 400 MHz).

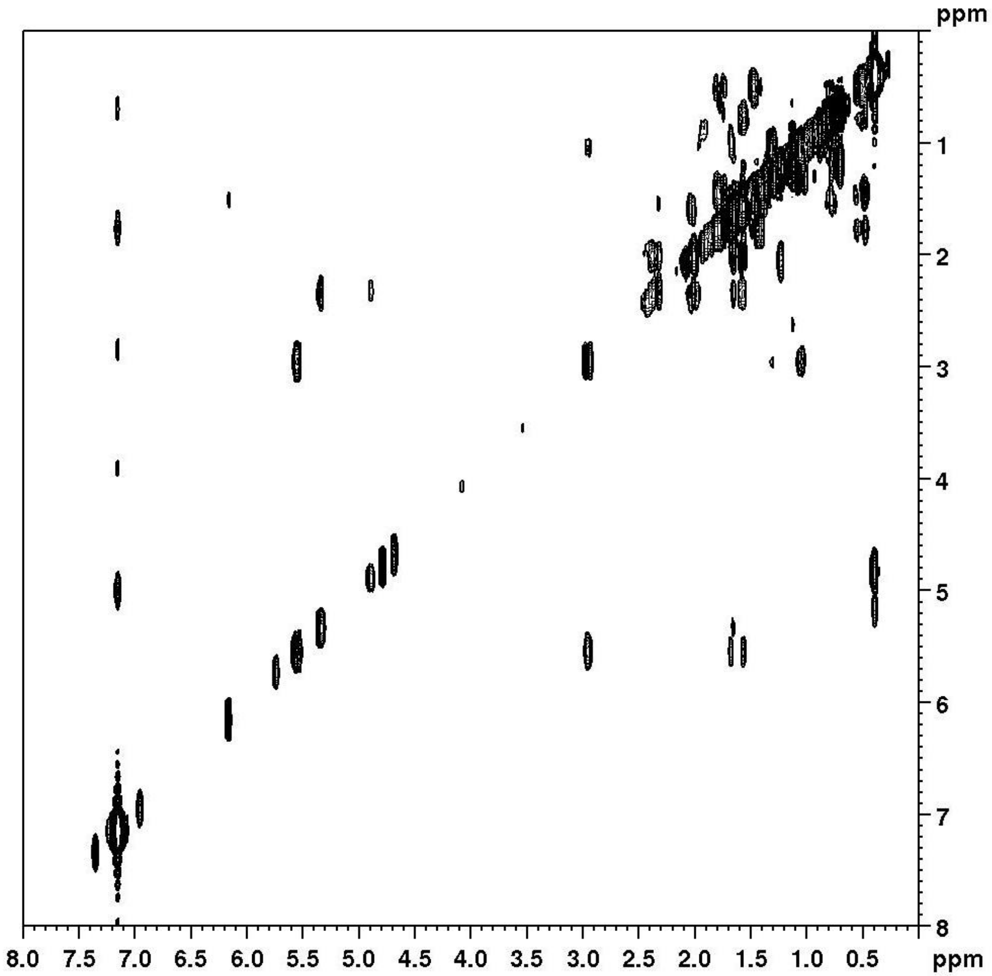

^{1}H-^{1}H COSY spectrum of 7-isocyanoamphilecta-10,14-diene (**5**) (C_6D_6, Bruker 400 MHz).

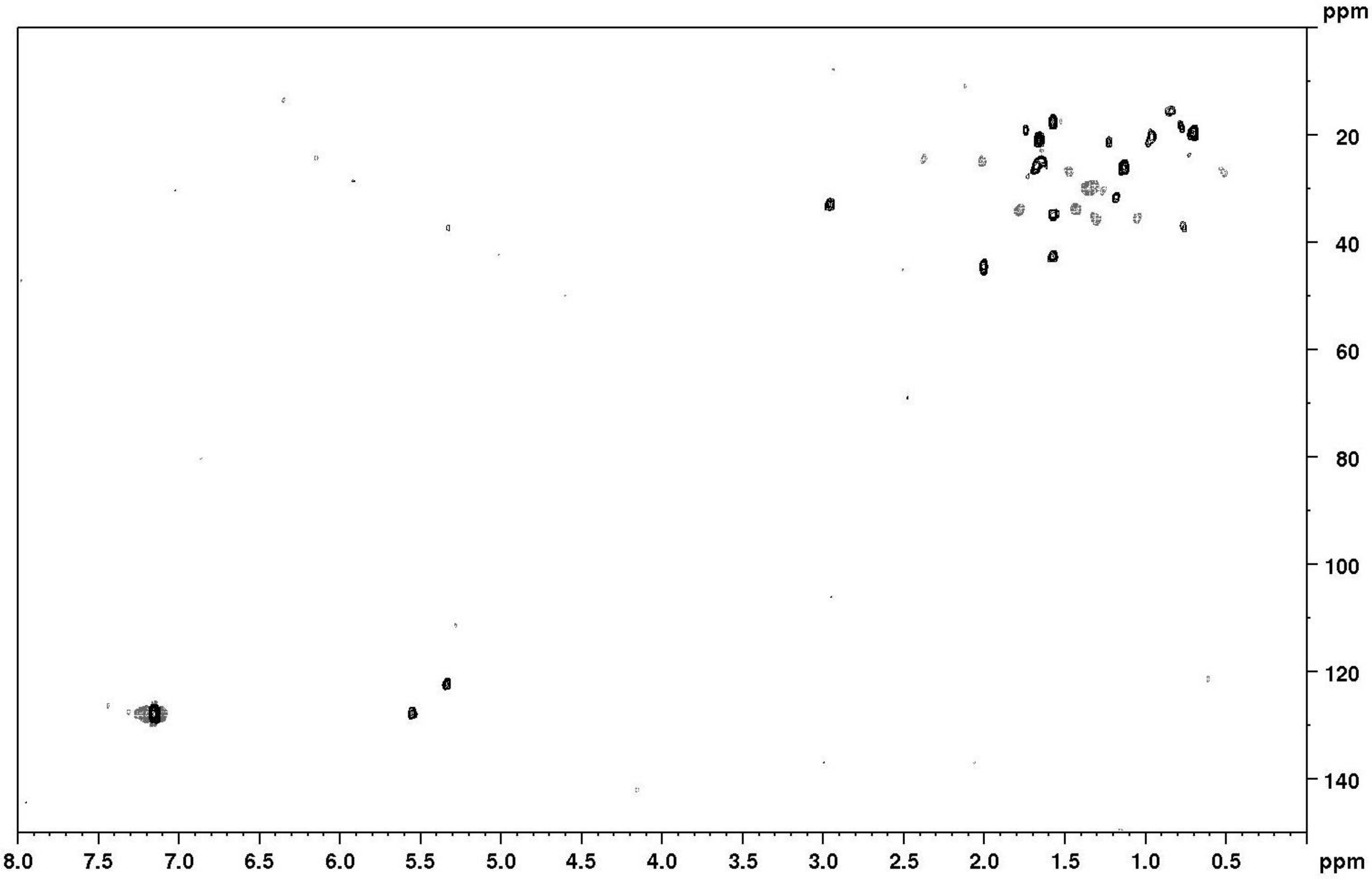

HSQC spectrum of 7-isocyanoamphilecta-10,14-diene (**5**) (C_6D_6, Bruker 400 MHz).

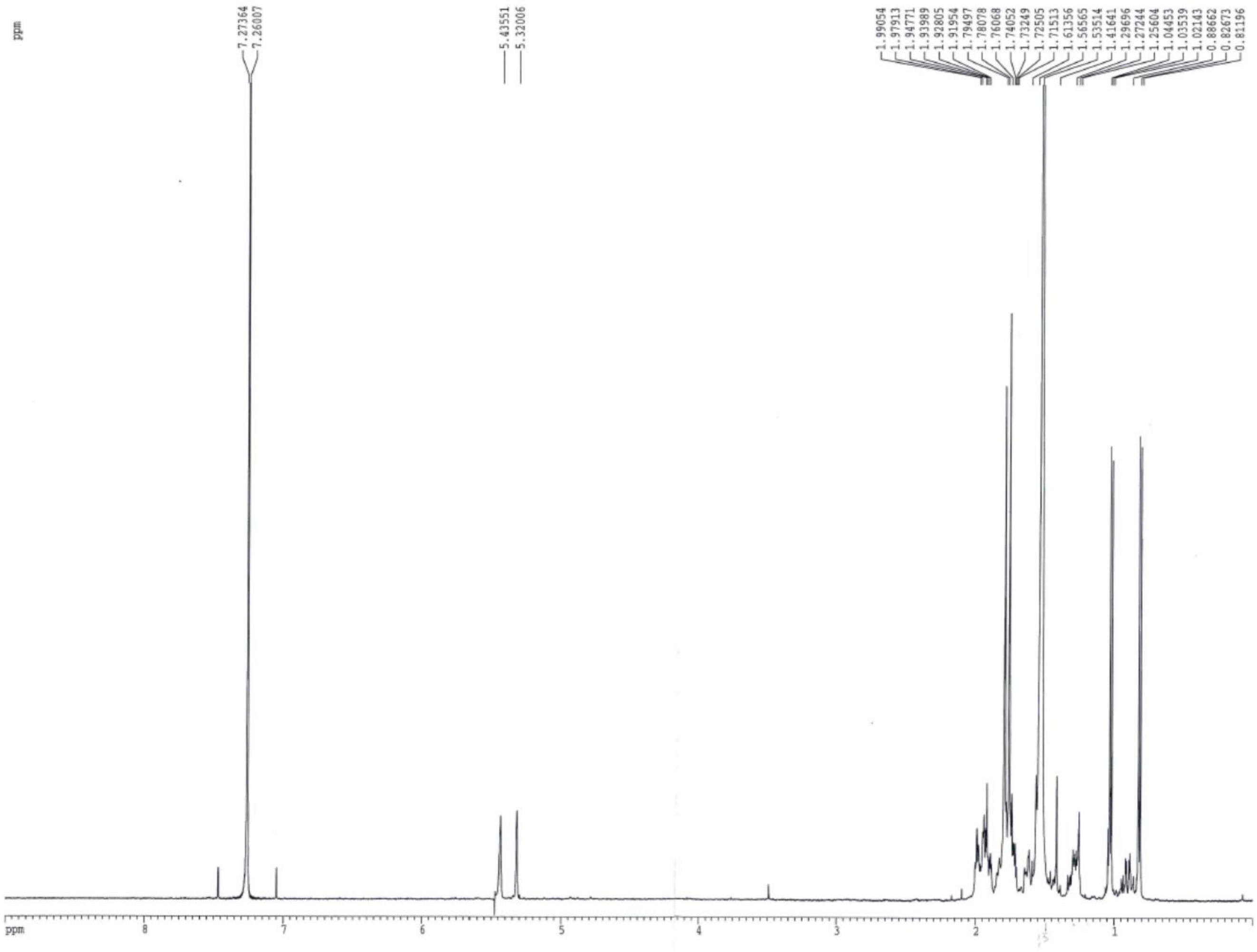

^1H NMR spectrum of 7-isocyanoamphilecta-1,14-diene (**6**) ($CDCl_3$, Bruker 500 MHz).

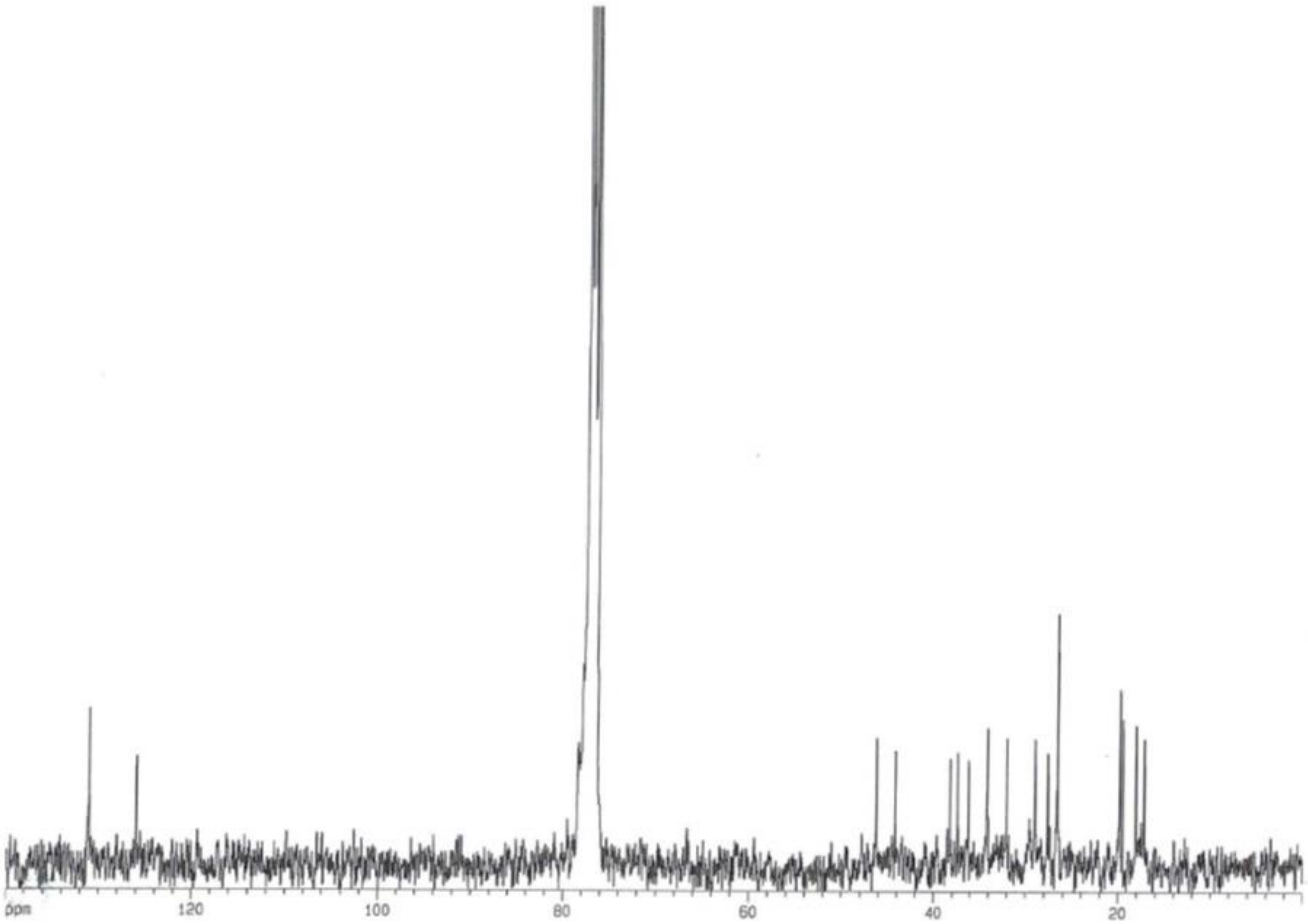

^{13}C NMR spectrum of 7-isocyanoamphilecta-1,14-diene (**6**) ($CDCl_3$, Bruker 300 MHz).

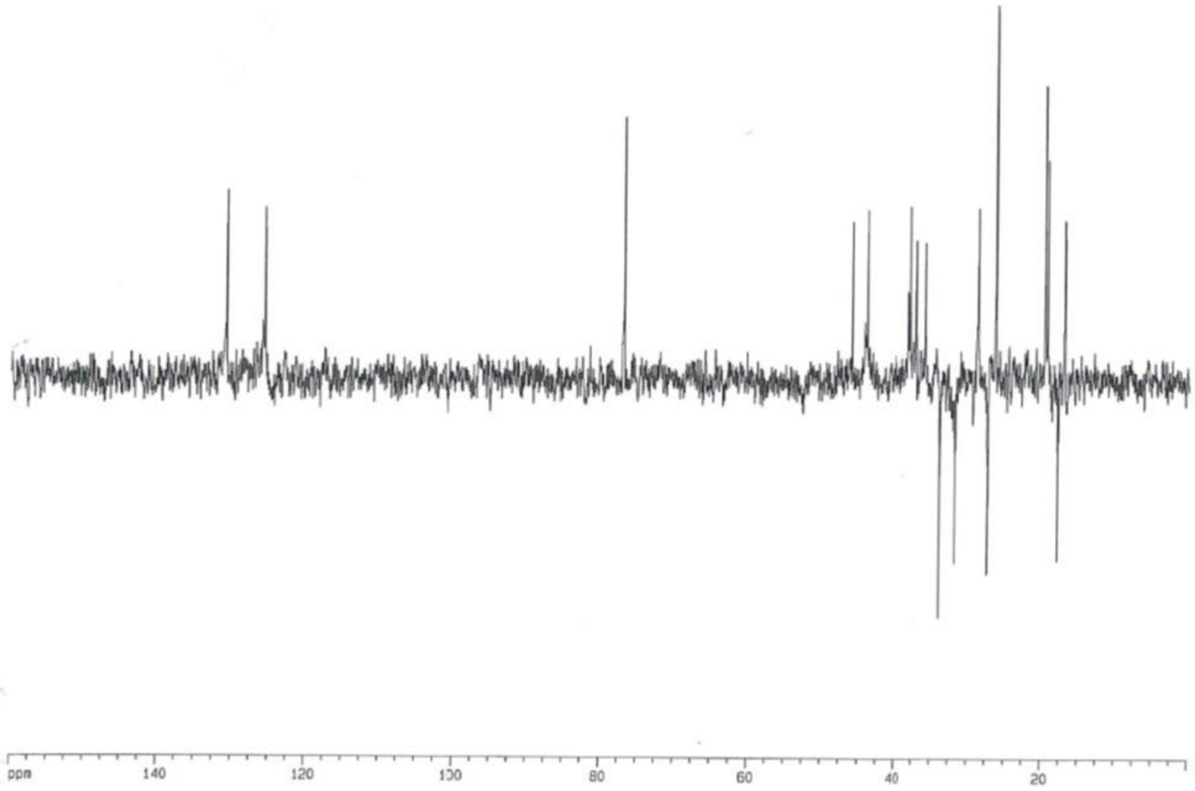

DEPT spectrum of 7-isocyanoamphilecta-1,14-diene (**6**) ($CDCl_3$, Bruker 300 MHz).

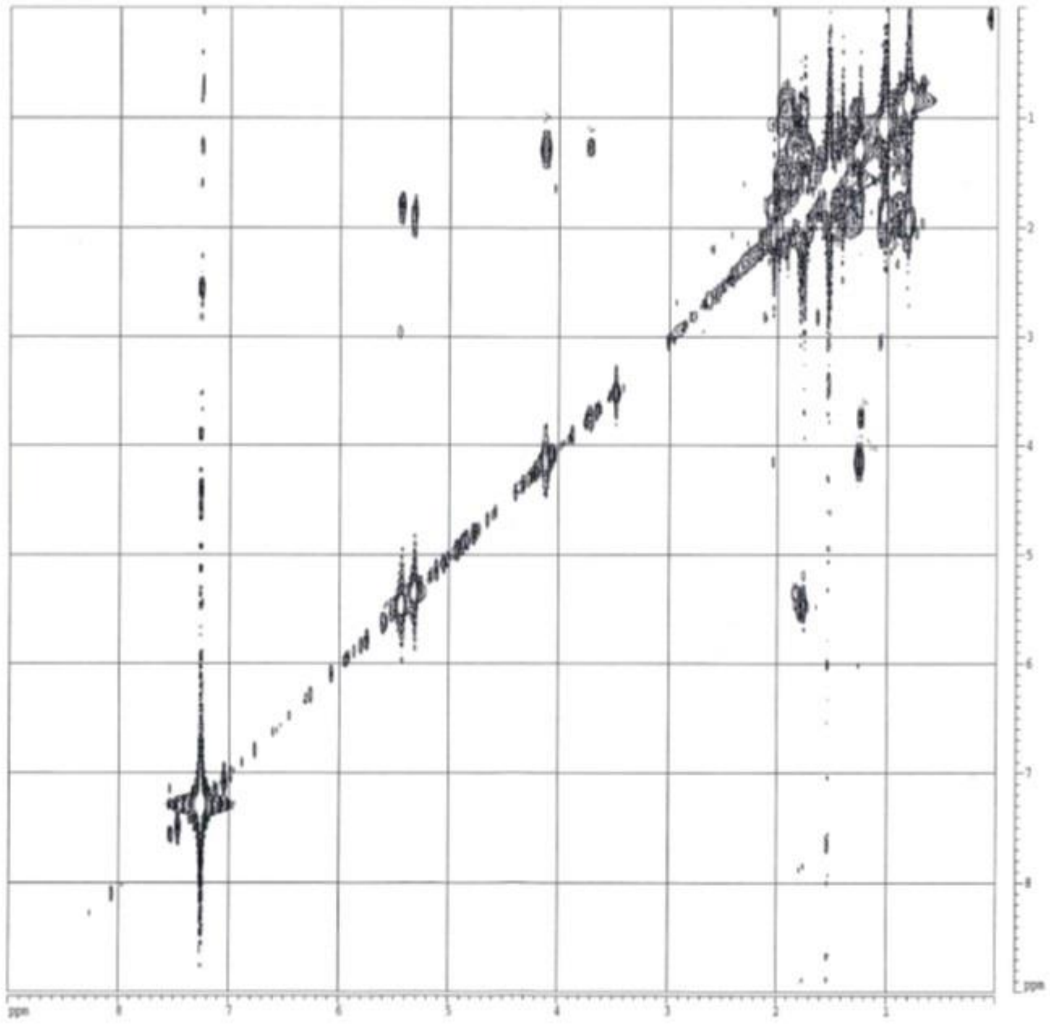

^{1}H-^{1}H COSY spectrum of 7-isocyanoamphilecta-1,14-diene (**6**) ($CDCl_3$, Bruker 500 MHz).

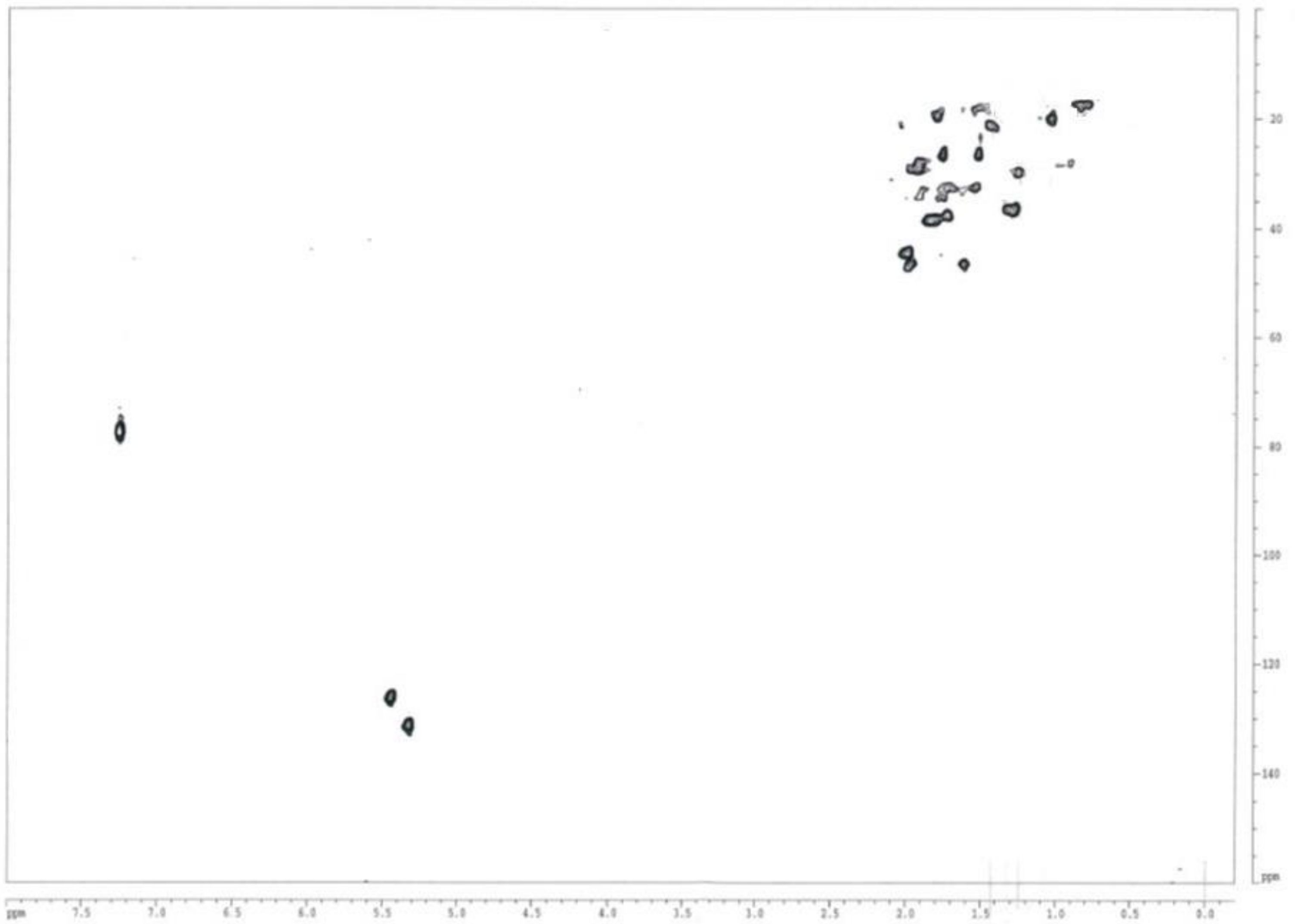

HSQC spectrum of 7-isocyanoamphilecta-1,14-diene (**6**) ($CDCl_3$, Bruker 500 MHz).

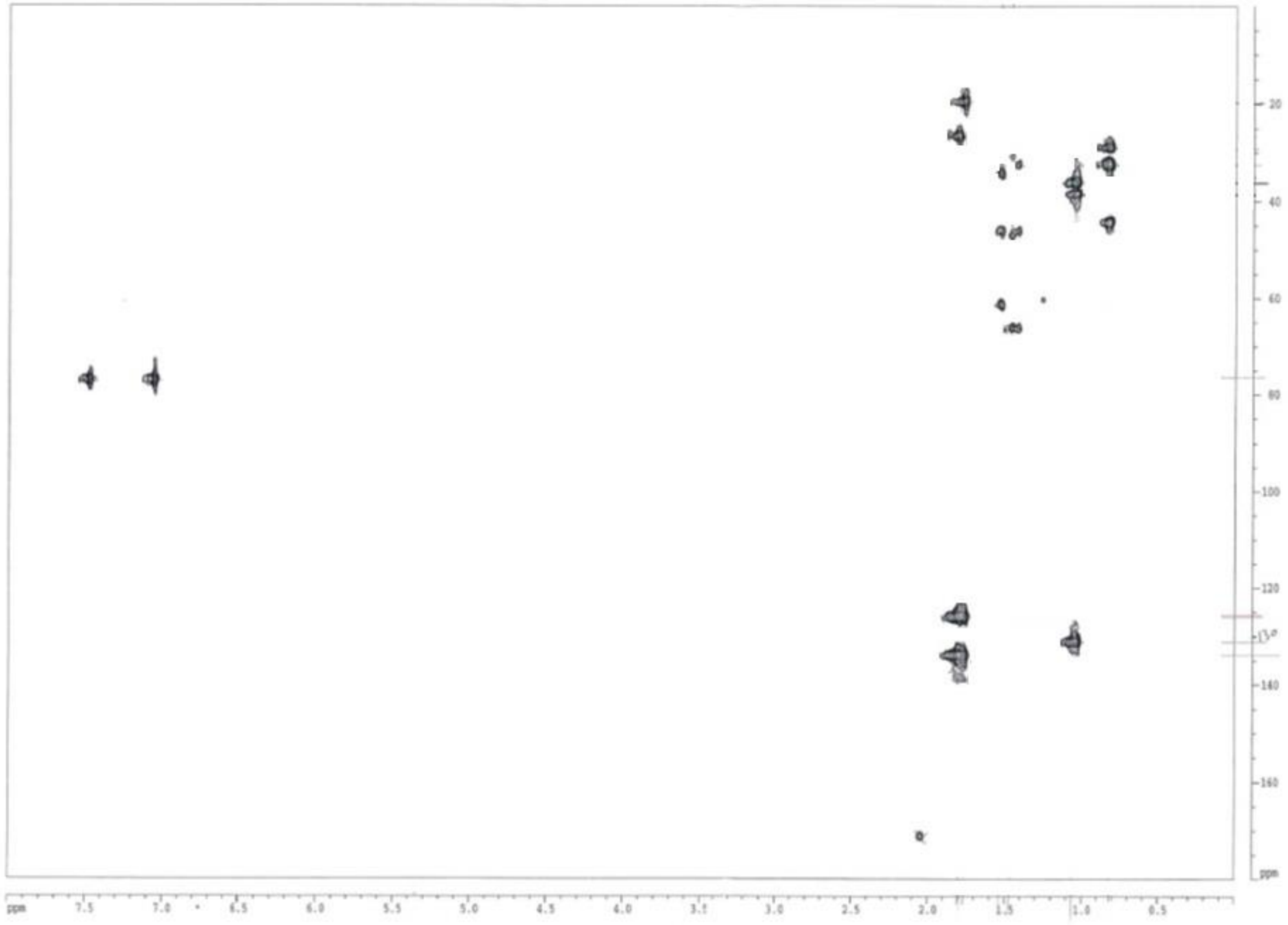

HMBC spectrum of 7-isocyanoamphilecta-1,14-diene (**6**) ($CDCl_3$, Bruker 500 MHz, *J*=7Hz).

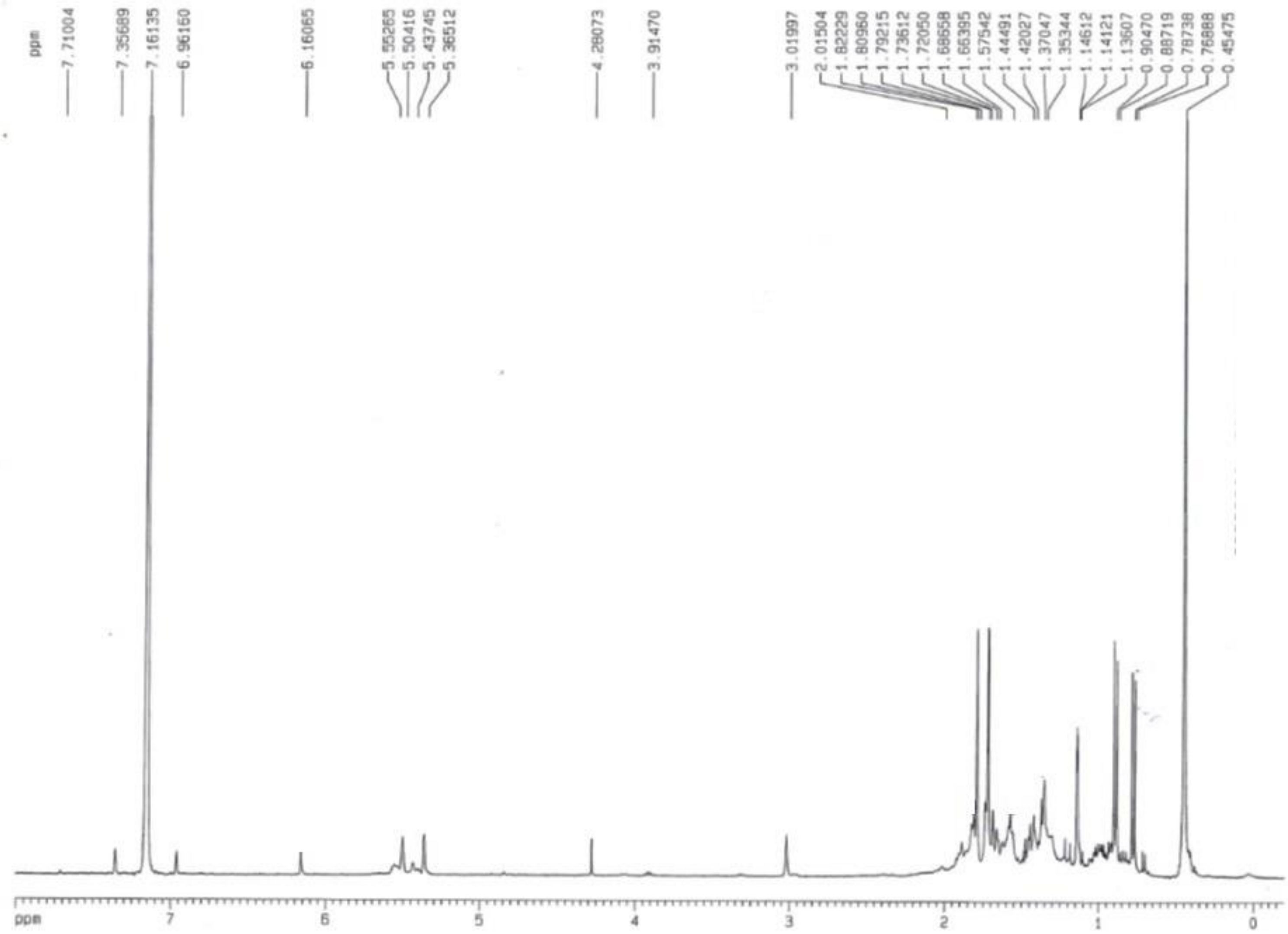

^{1}H NMR spectrum of 7-isocyanoamphilecta-1,14-diene (**6**) (C_6D_6, Bruker 500 MHz).

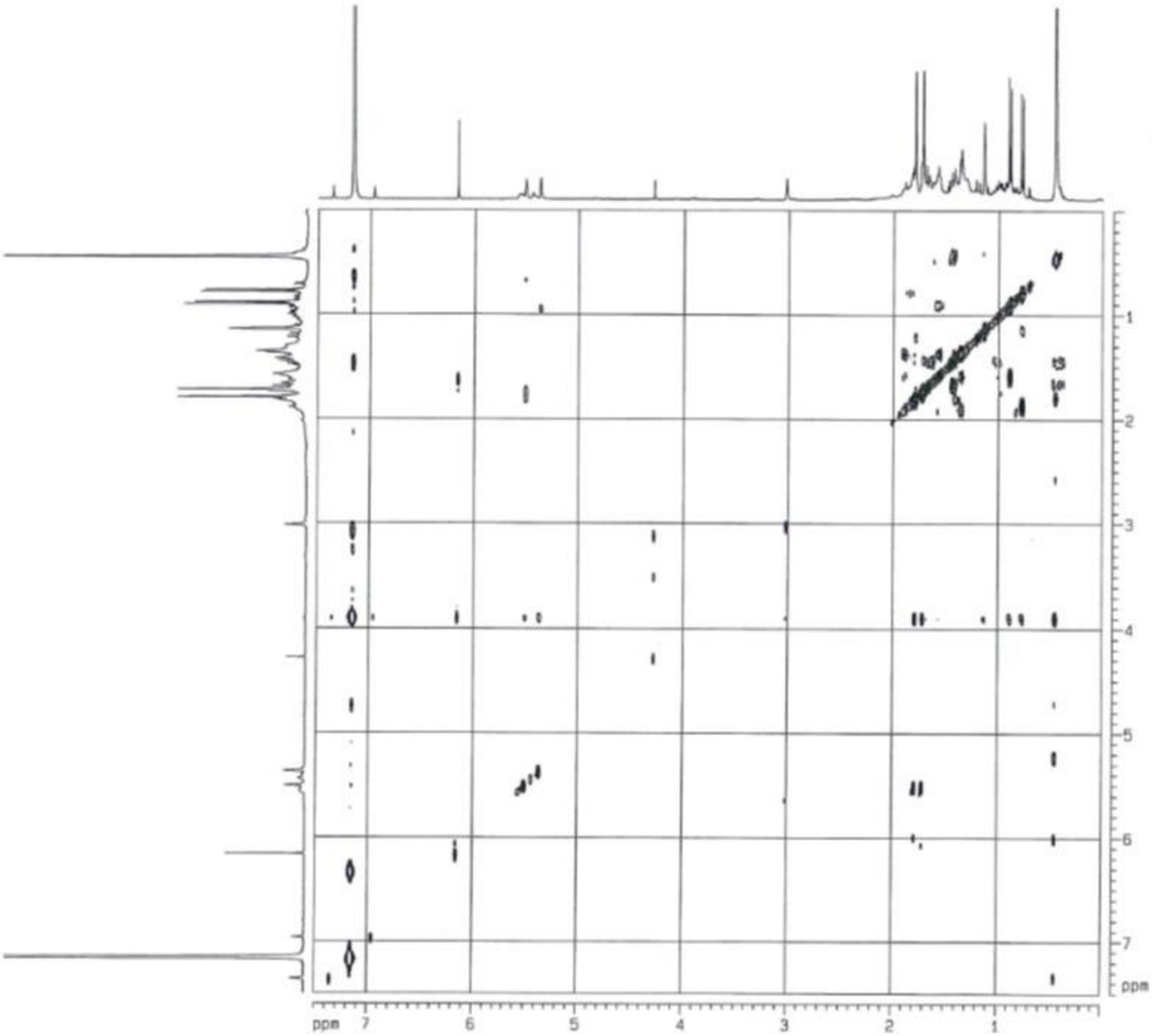

^{1}H-^{1}H COSY spectrum of 7-isocyanoamphilecta-1,14-diene (**6**) (C_6D_6, Bruker 500 MHz).

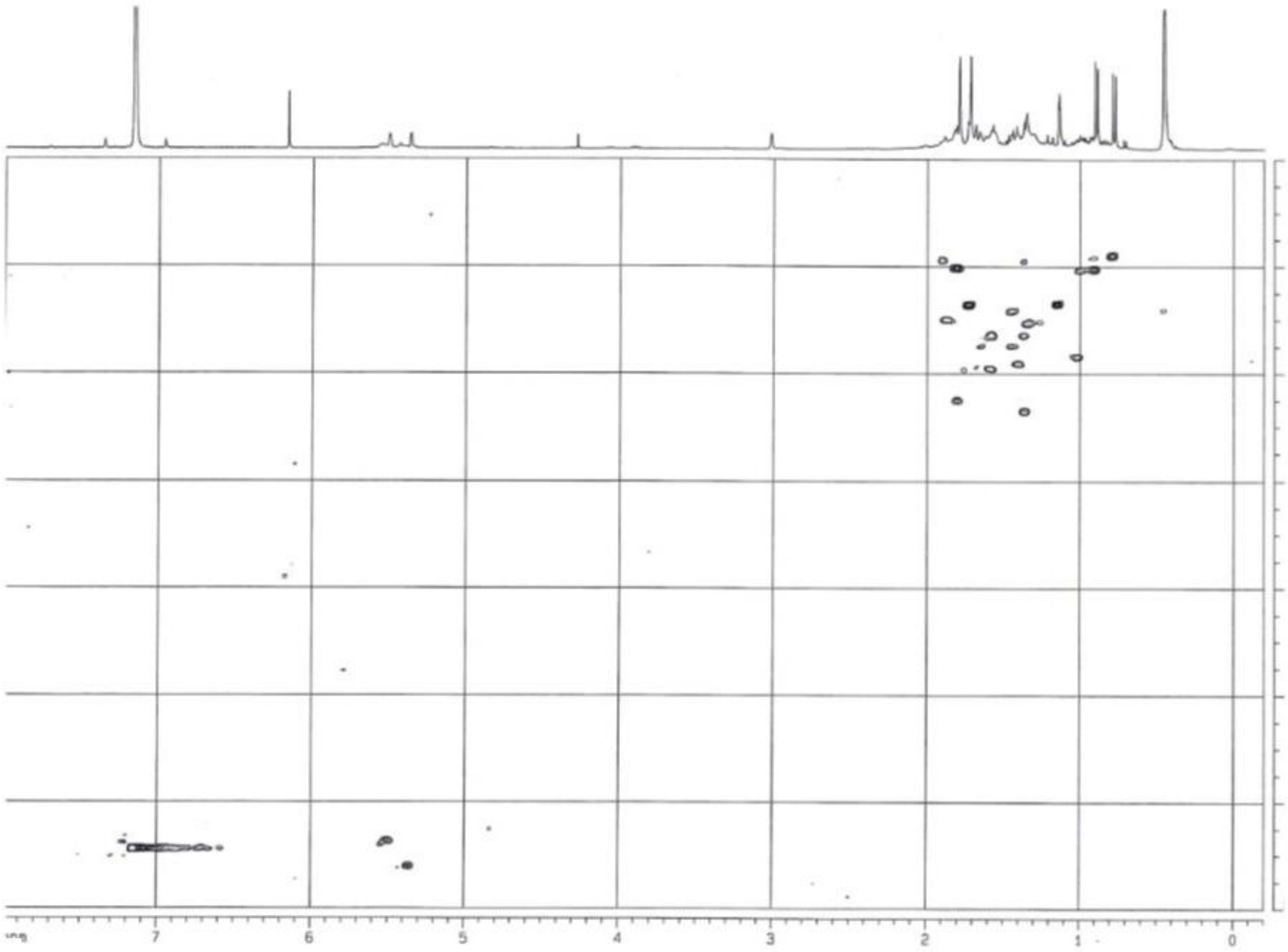

HSQC spectrum of 7-isocyanoamphilecta-1,14-diene (**6**) (C_6D_6, Bruker 500 MHz).

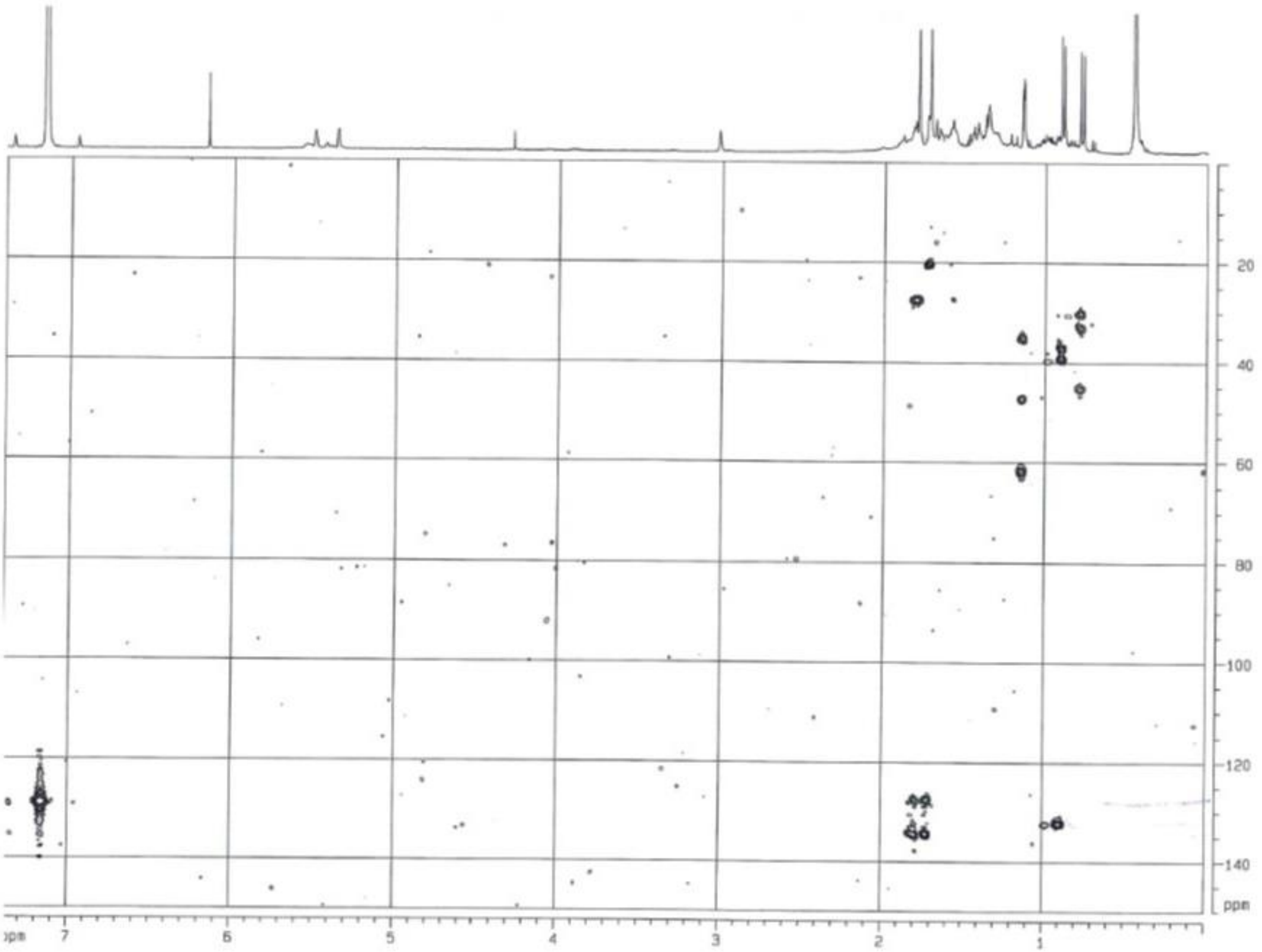

HMBC spectrum of 7-isocyanoamphilecta-1,14-diene (**6**) (C_6D_6, 500 MHz, *J*=7Hz).

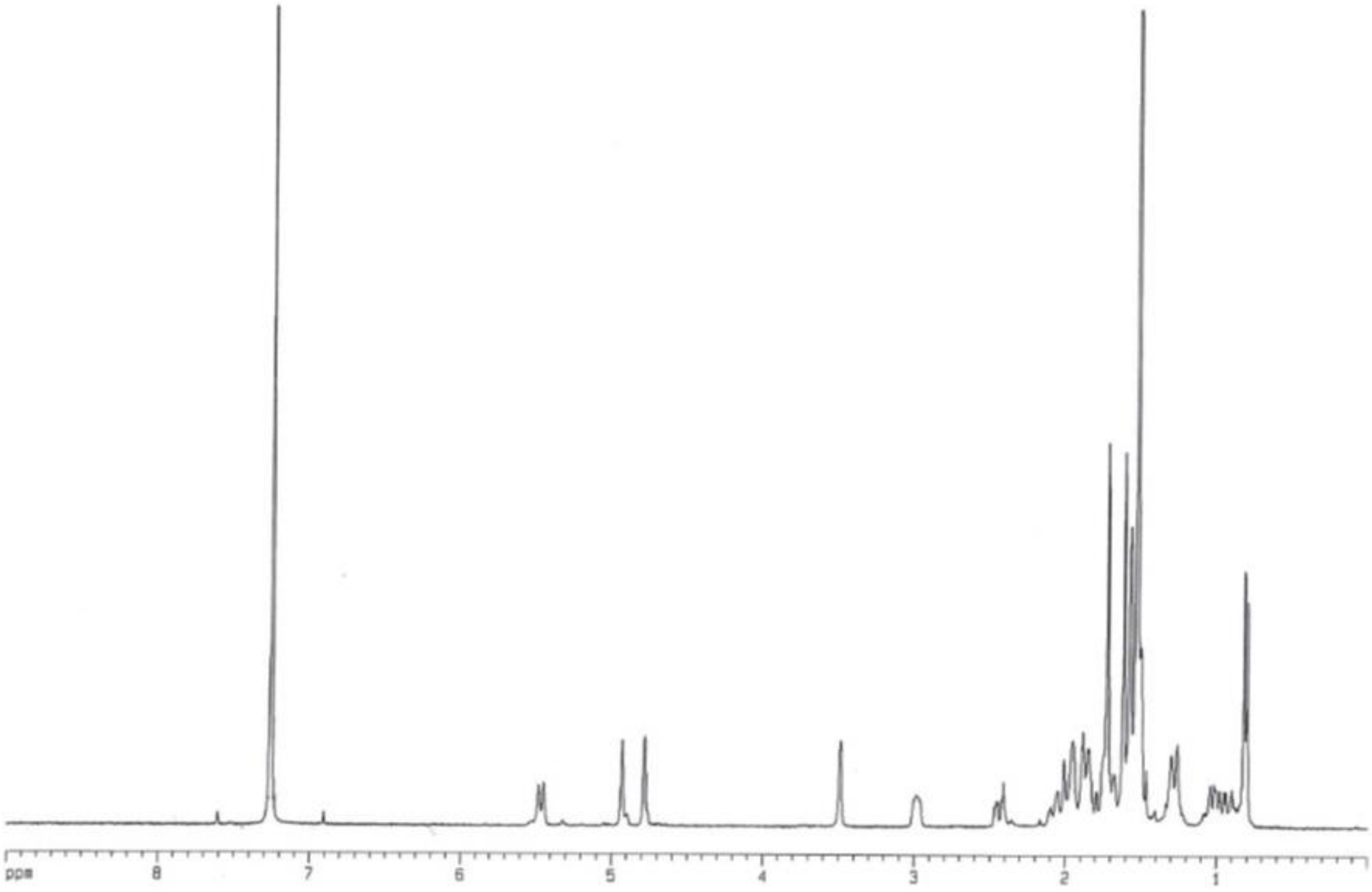

^{1}H NMR spectrum of 7-isocyanoamphilecta-11(20),14-diene (**7**) ($CDCl_3$, Bruker 300 MHz).

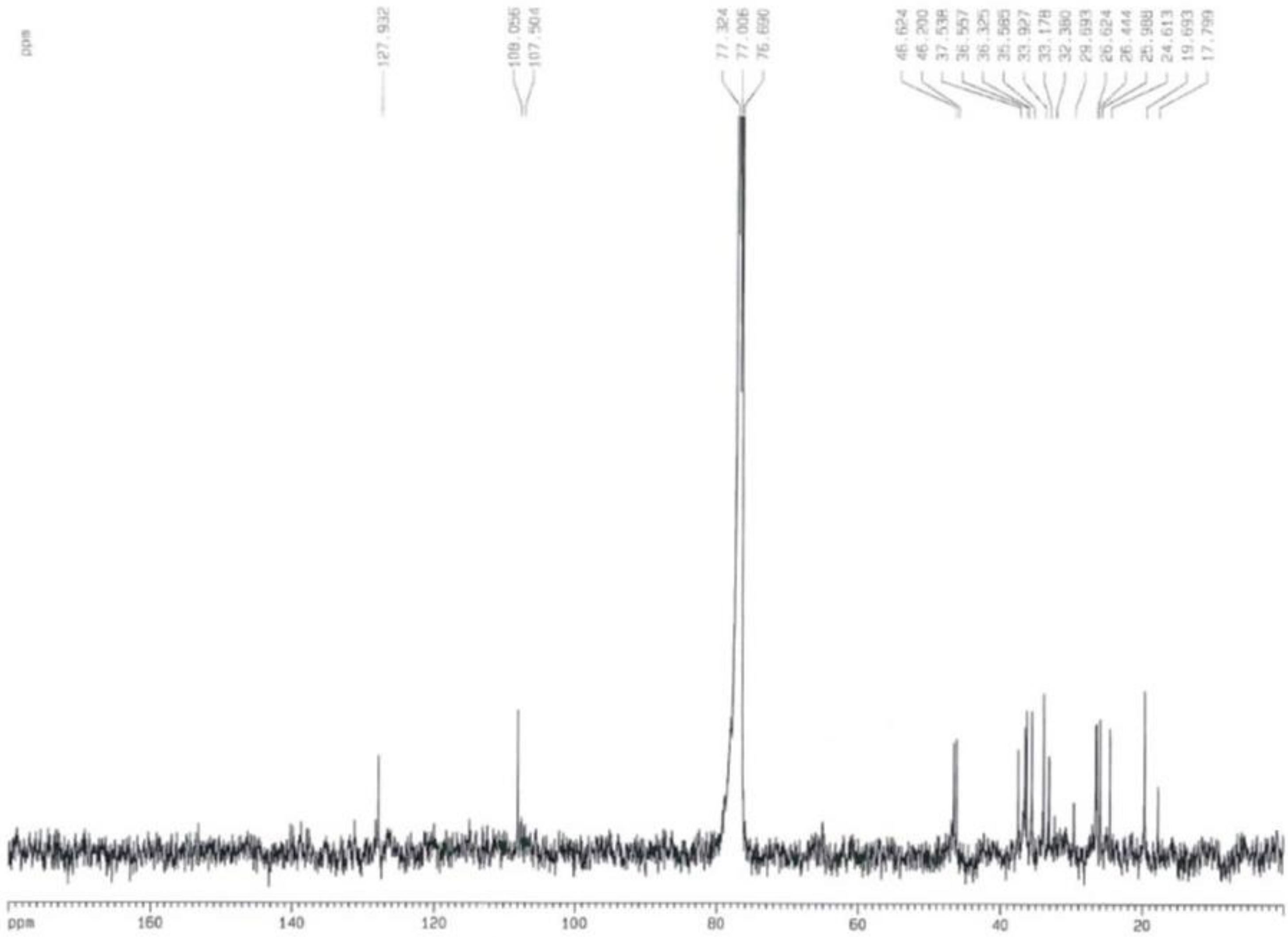

^{13}C NMR spectrum of 7-isocyanoamphilecta-11(20),14-diene (**7**) ($CDCl_3$, Bruker 300 MHz).

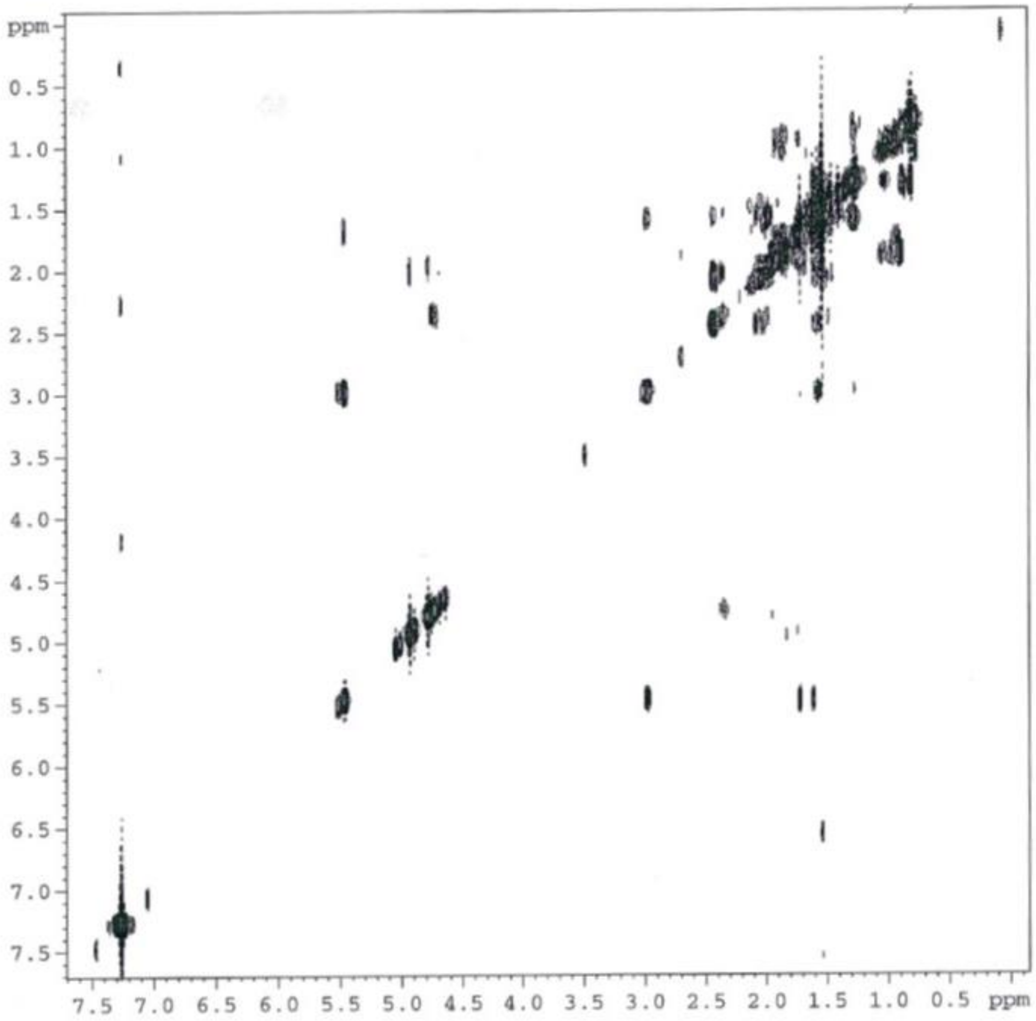

^{1}H-^{1}H COSY spectrum of 7-isocyanoamphilecta-11(20),14-diene (**7**) ($CDCl_3$, Bruker 500 MHz).

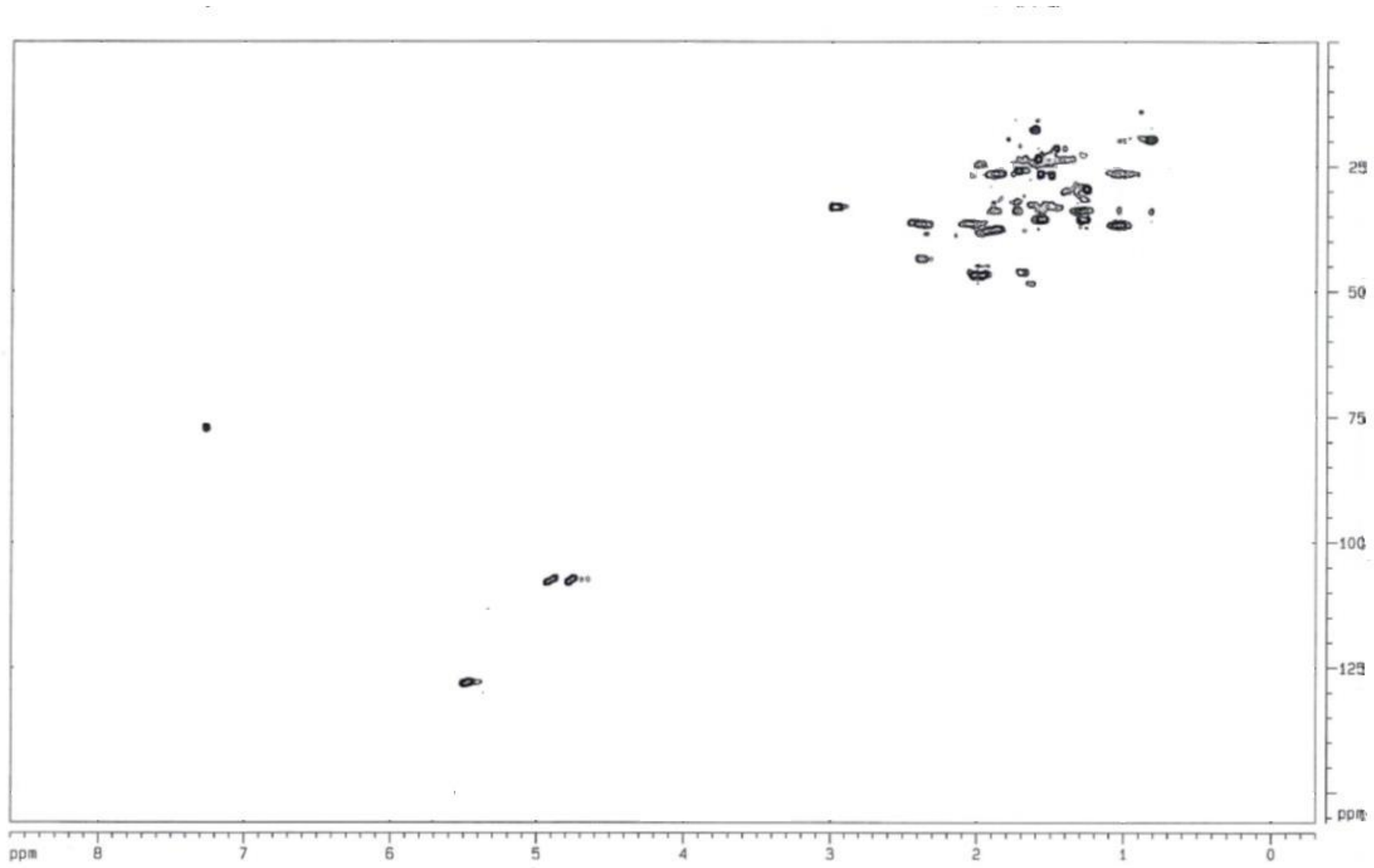

HSQC spectrum of 7-isocyanoamphilecta-11(20),14-diene (**7**) ($CDCl_3$, Bruker 500 MHz).

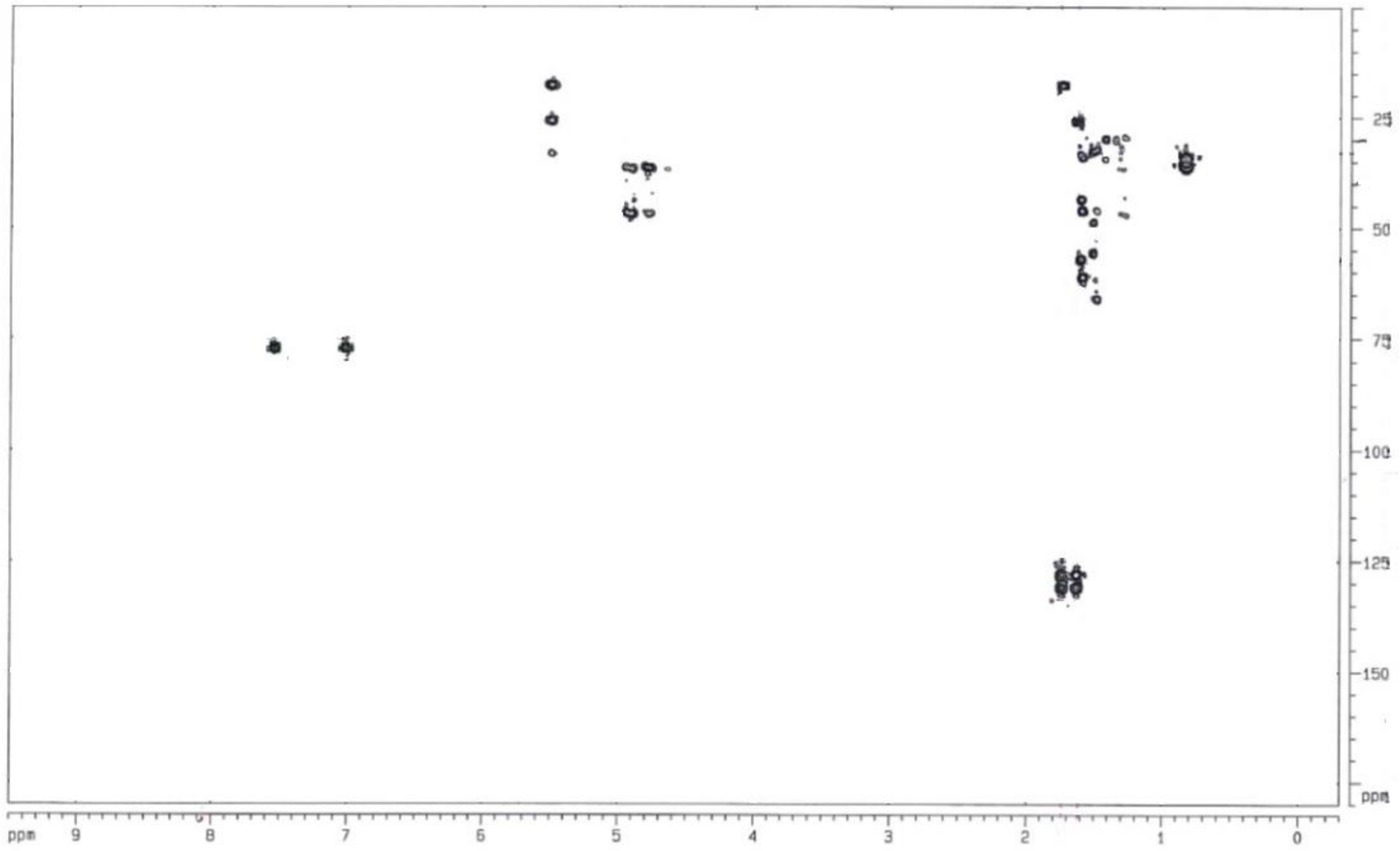

HMBC spectrum of 7-isocyanoamphilecta-11(20),14-diene (**7**) ($CDCl_3$, Bruker 500 MHz, *J*=7Hz).

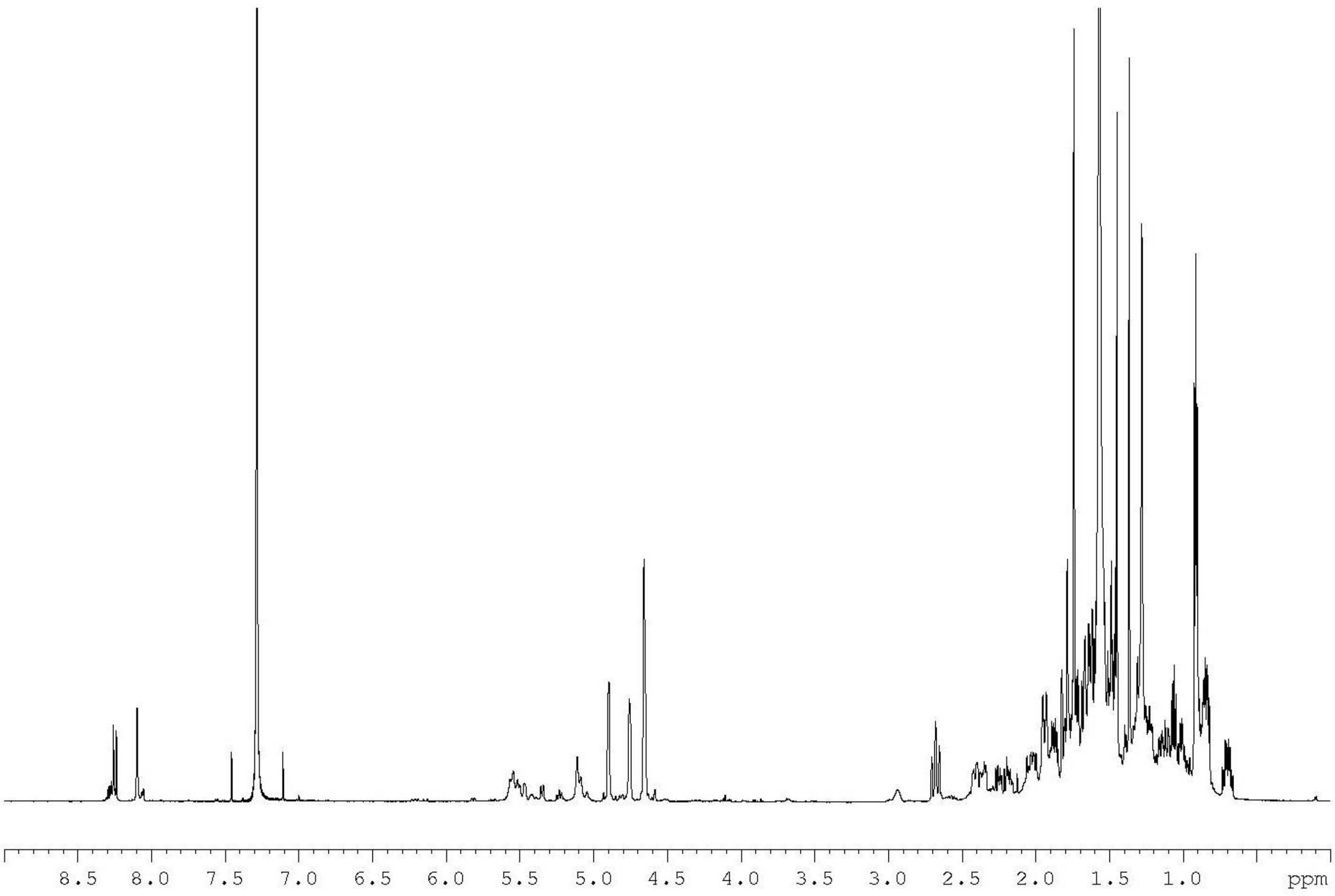

^{1}H NMR spectrum of 7-formamidoamphilecta-11(20),15-diene (**8**) ($CDCl_3$, Bruker 600 MHz).

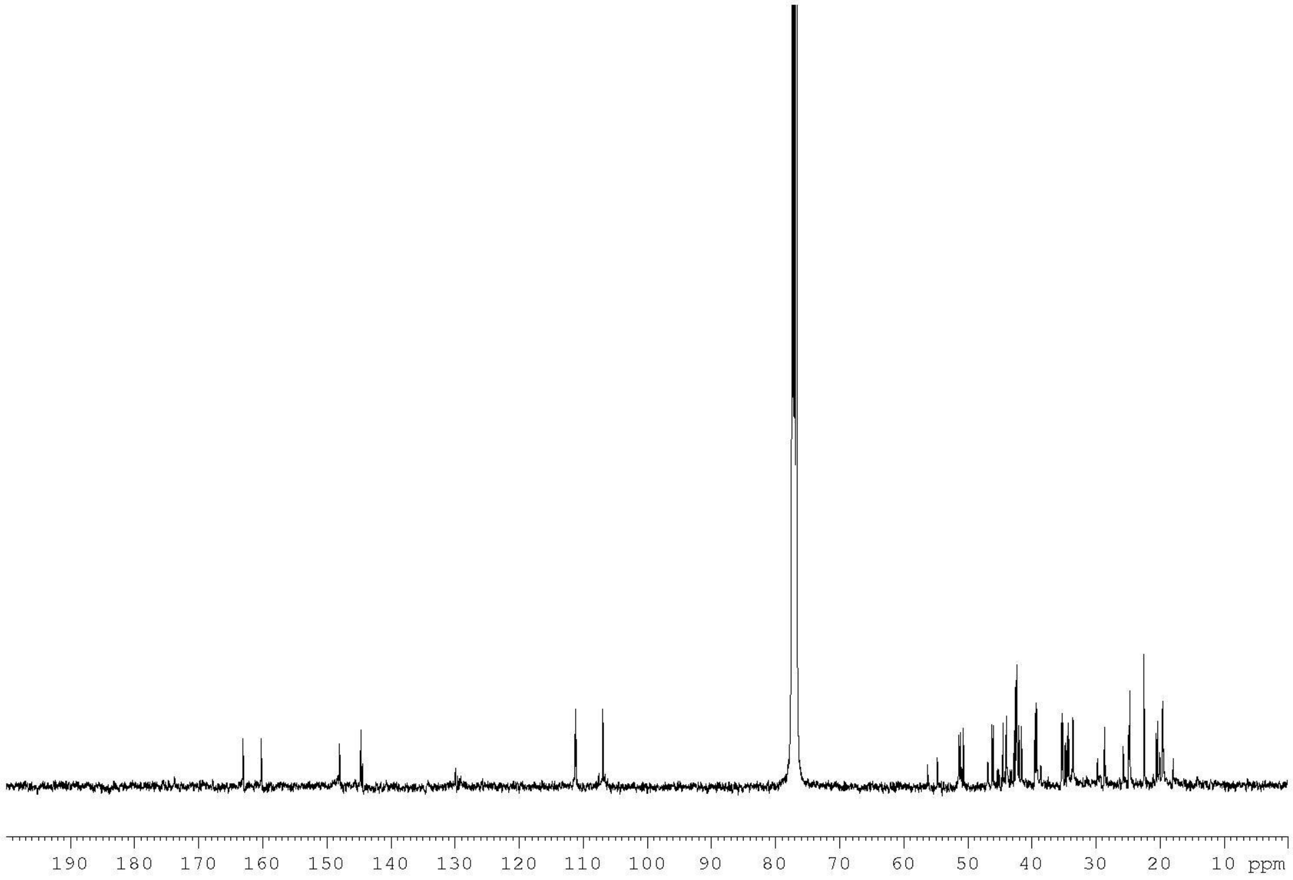

^{13}C NMR spectrum of 7-formamidoamphilecta-11(20),15-diene (**8**) ($CDCl_3$, Bruker 400 MHz).

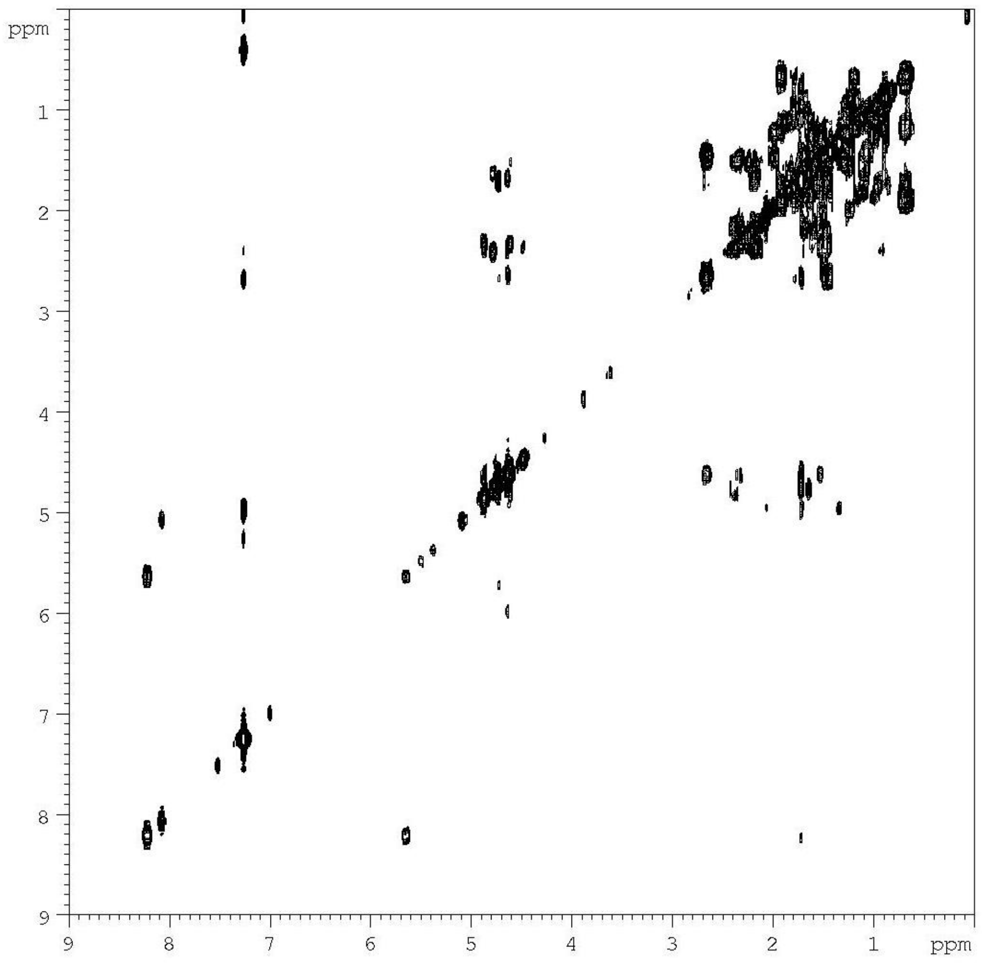

^{1}H-^{1}H COSY spectrum of 7-formamidoamphilecta-11(20),15-diene (**8**) ($CDCl_3$, Bruker 600 MHz).

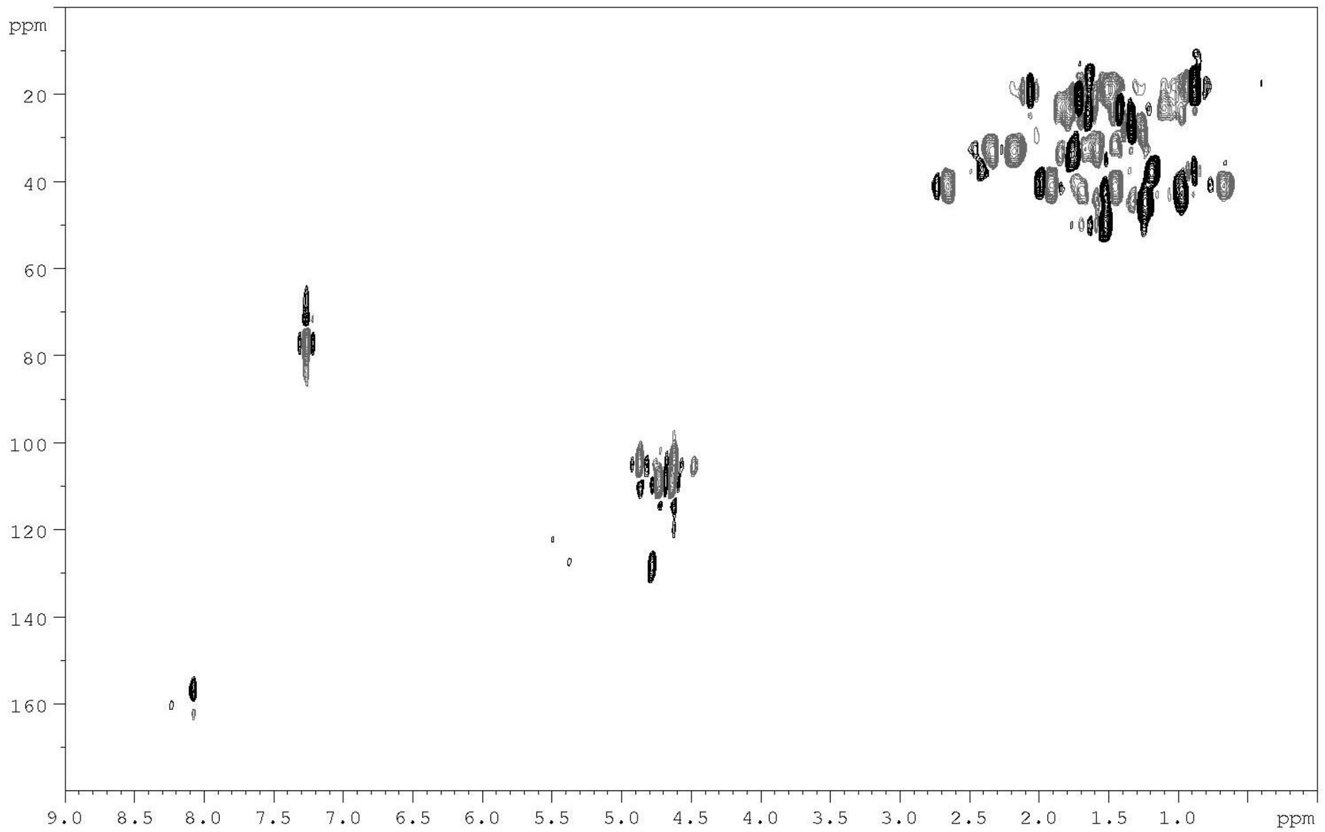

HSQC spectrum of 7-formamidoamphilecta-11(20),15-diene (**8**) ($CDCl_3$, Bruker 600 MHz).

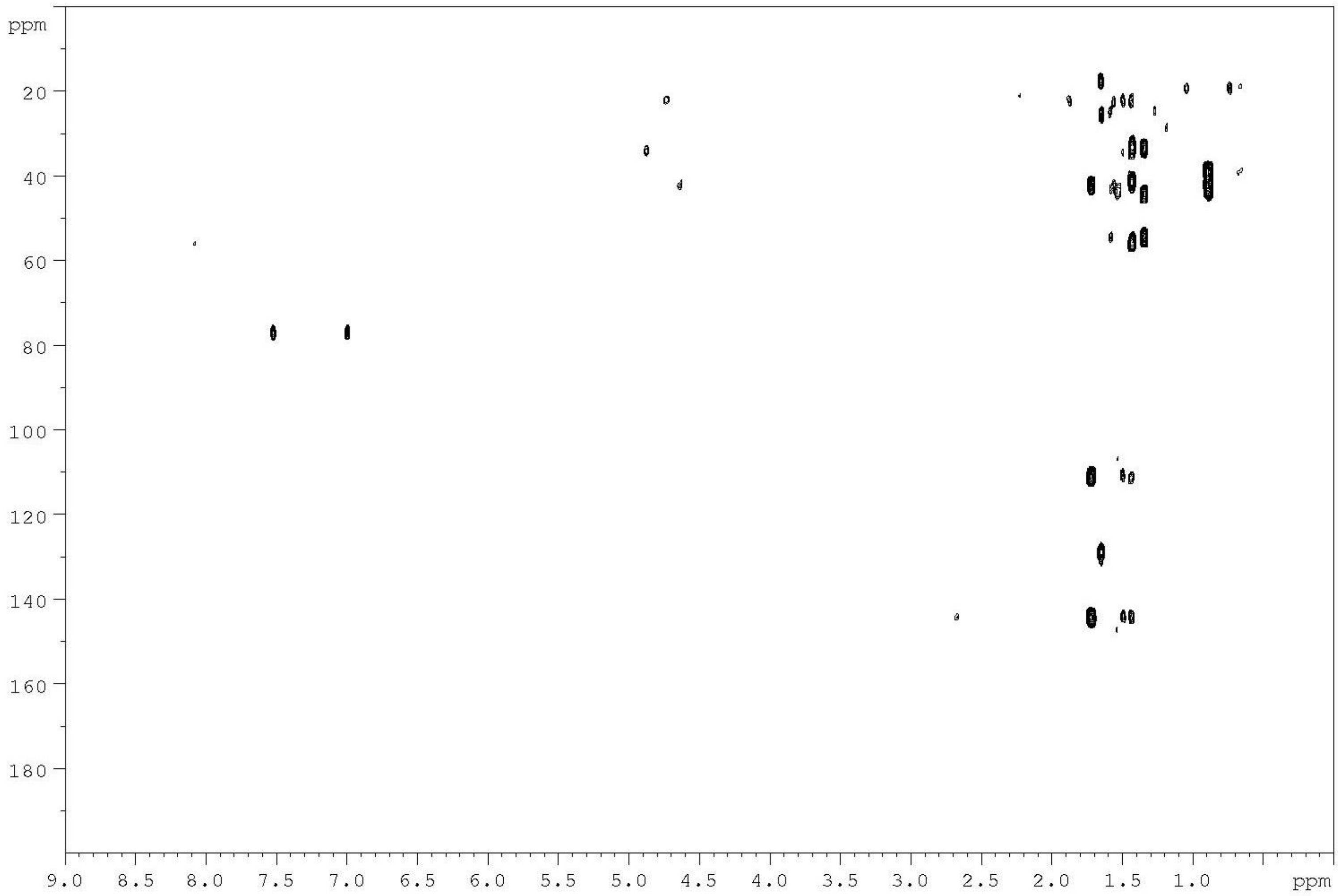

HMBC spectrum of 7-formamidoamphilecta-11(20),15-diene (**8**) ($CDCl_3$, Bruker 600 MHz, *J*=7Hz).

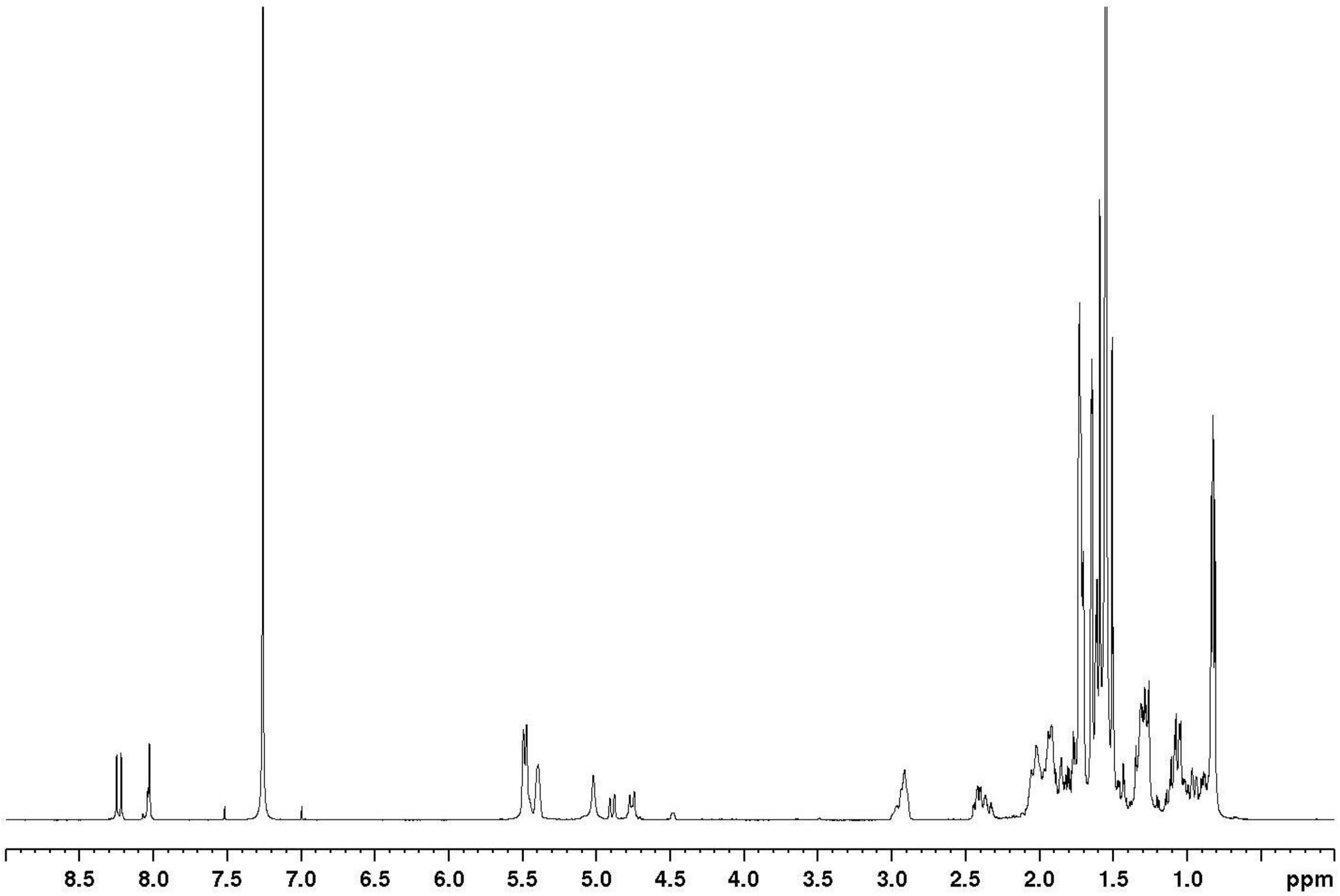

^{1}H NMR spectrum of 7-formamido amphilecta-10,14-diene (**9**) ($CDCl_3$, Bruker 400 MHz).

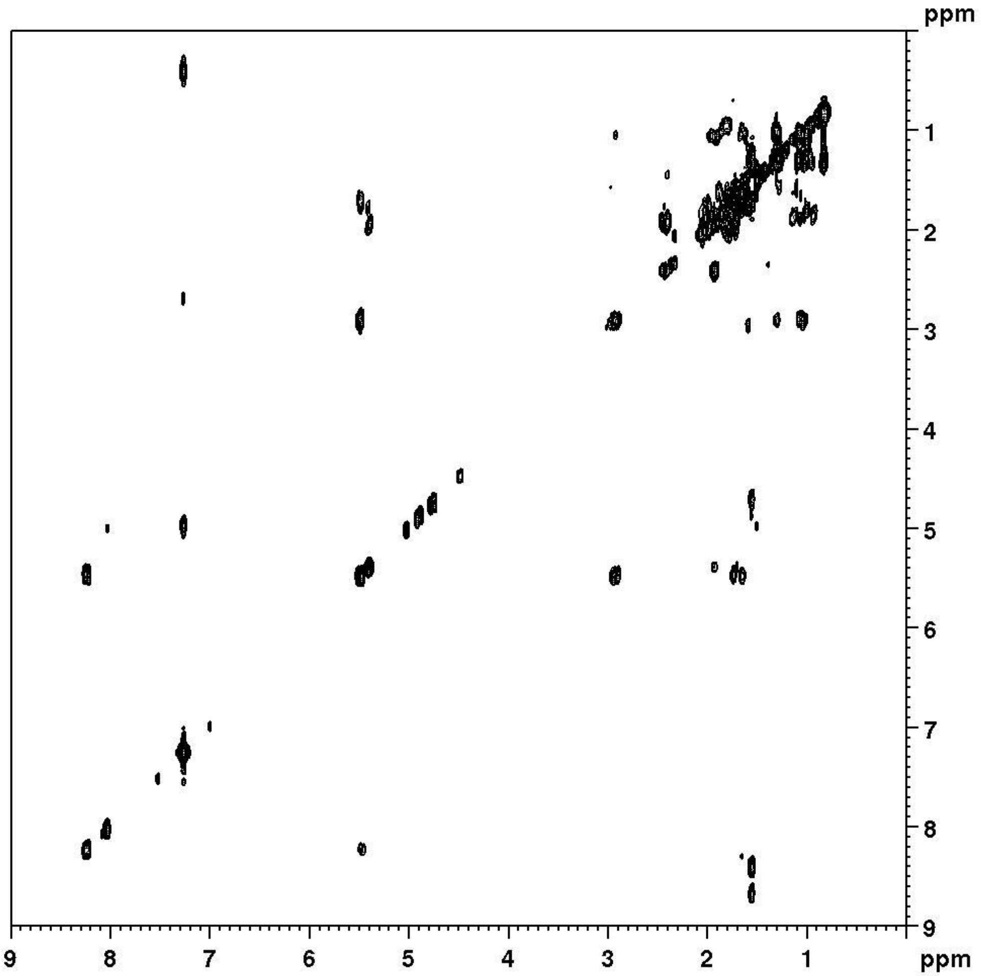

^{1}H-^{1}H COSY spectrum of 7-formamido amphilecta-10,14-diene (**9**) ($CDCl_3$, Bruker 400 MHz).

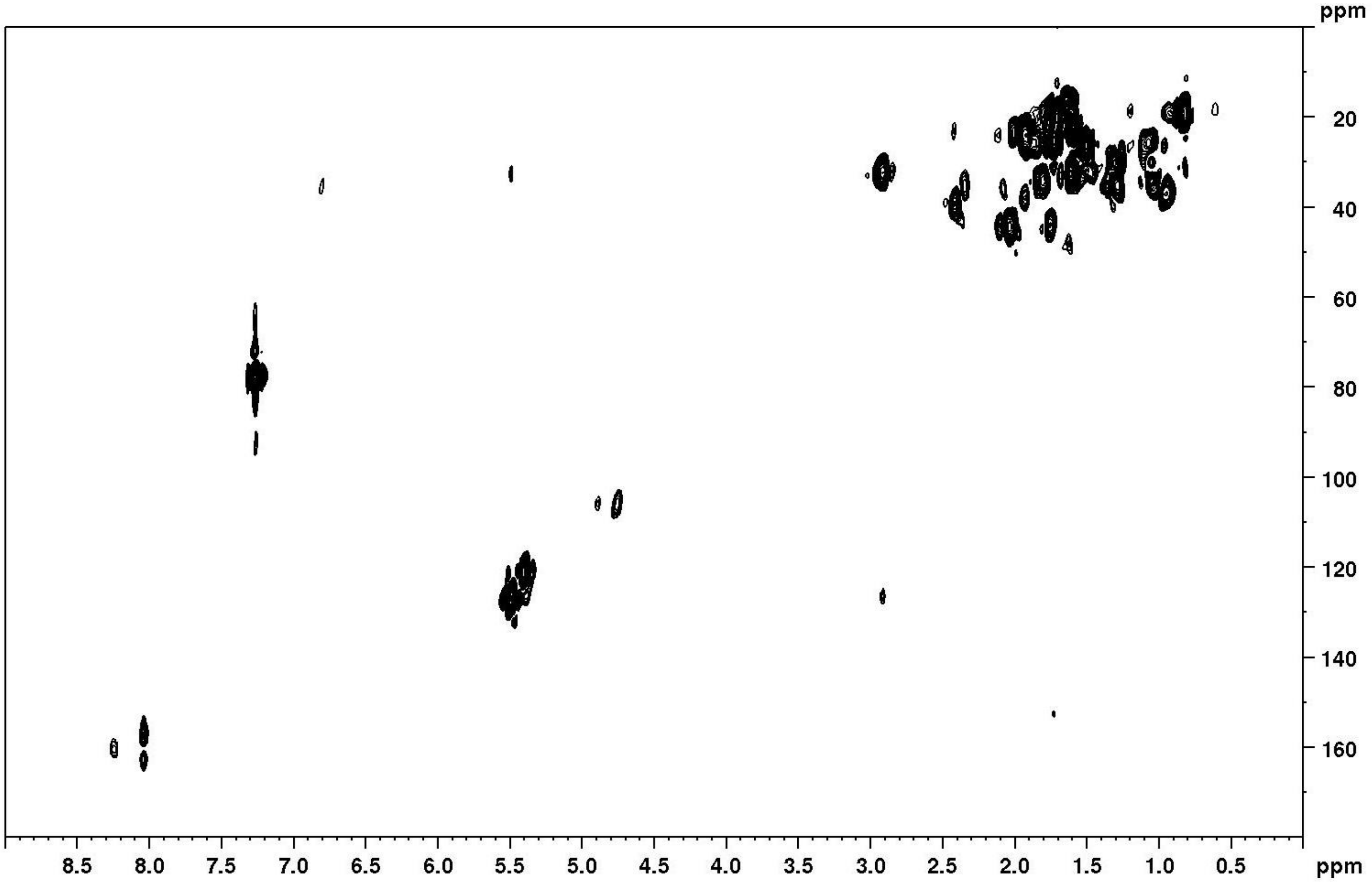

HSQC spectrum of 7-formamido amphilecta-10,14-diene (**9**) ($CDCl_3$, Bruker 400 MHz).

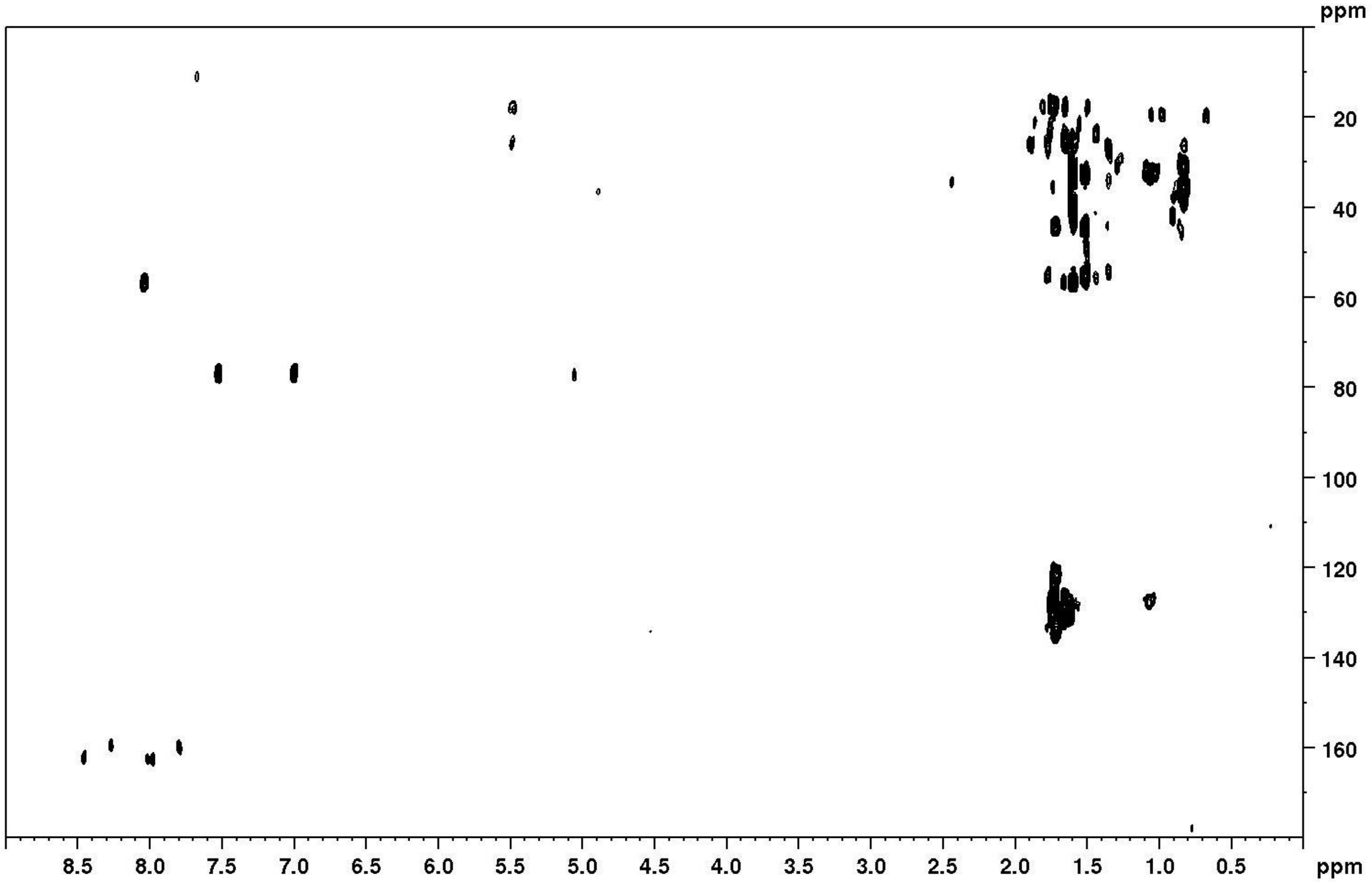

HMBC spectrum of 7-formamido amphilecta-10,14-diene (**9**) ($CDCl_3$, Bruker 400 MHz, *J*=7Hz).

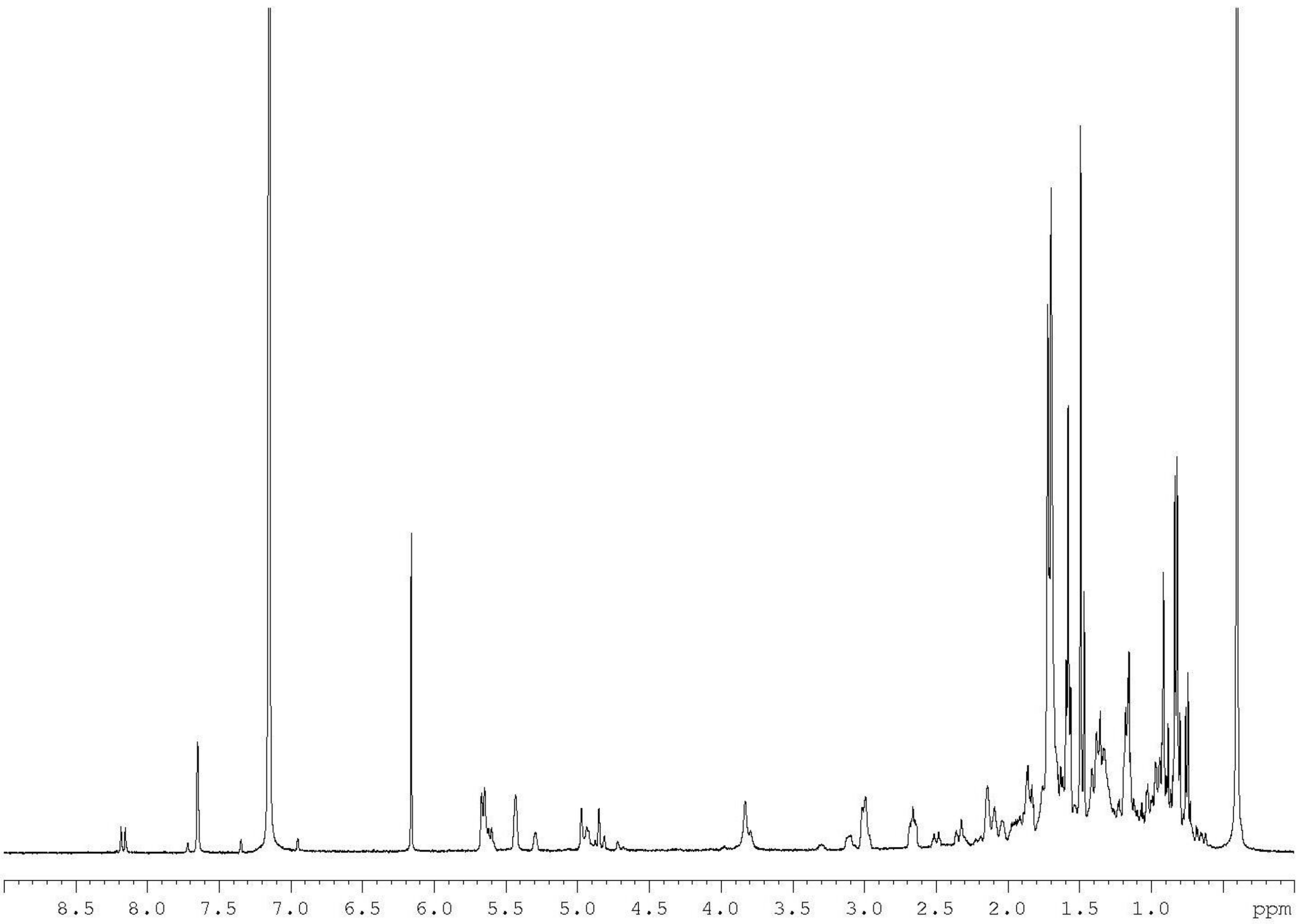

^{1}H NMR spectrum of 7-formamido amphilecta-10,14-diene (**9**) (C_6D_6, Bruker 400 MHz).

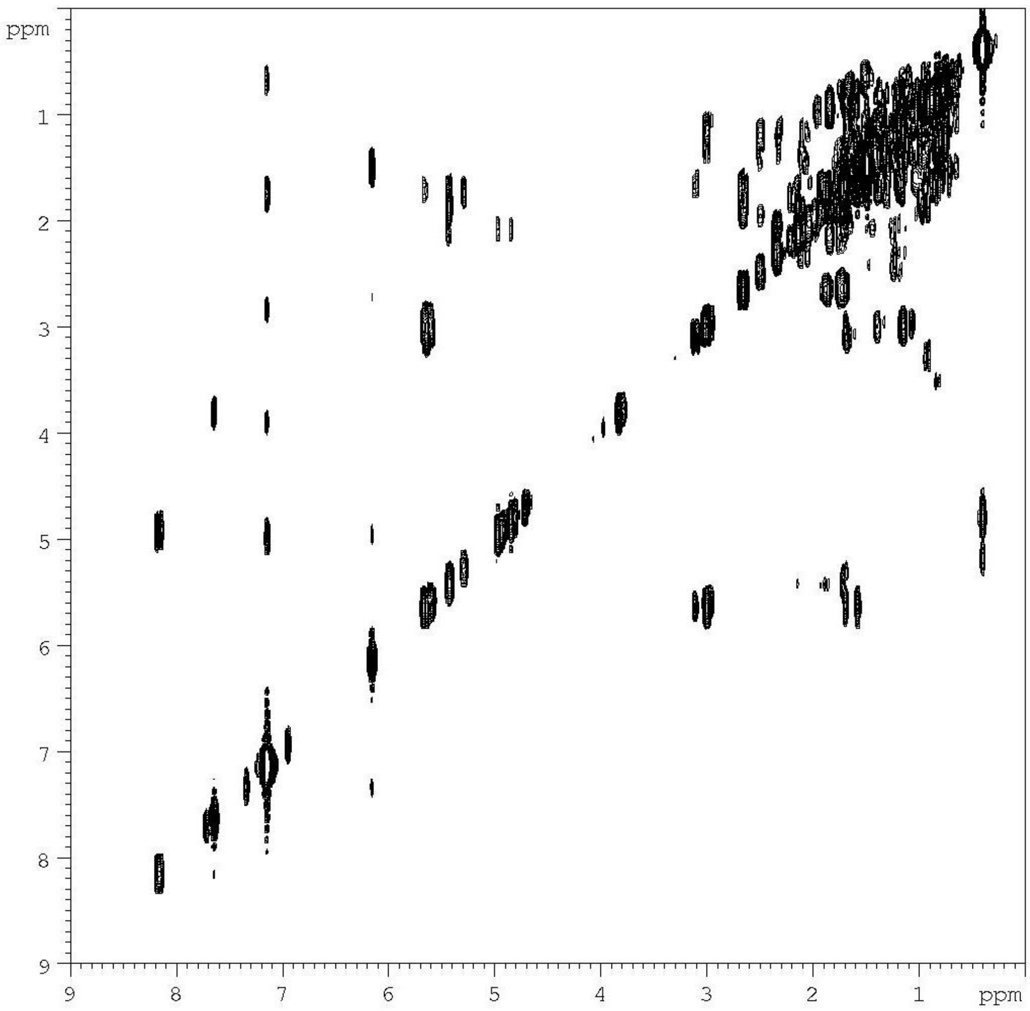

^{1}H-^{1}H COSY spectrum of 7-formamido amphilecta-10,14-diene (**9**) (C_6D_6, Bruker 400 MHz).

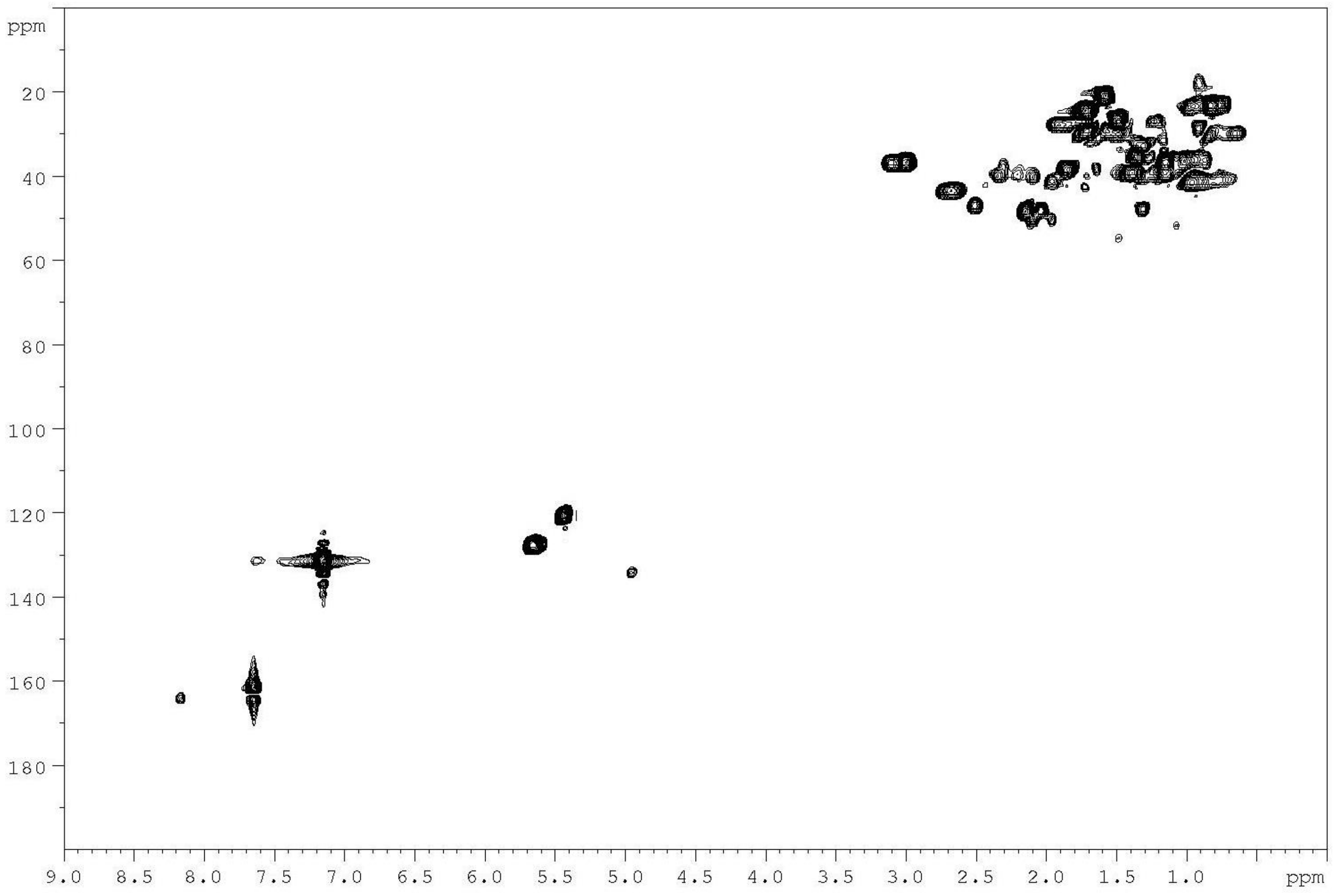

HSQC spectrum of 7-formamido amphilecta-10,14-diene (**9**) (C_6D_6, Bruker 400 MHz).

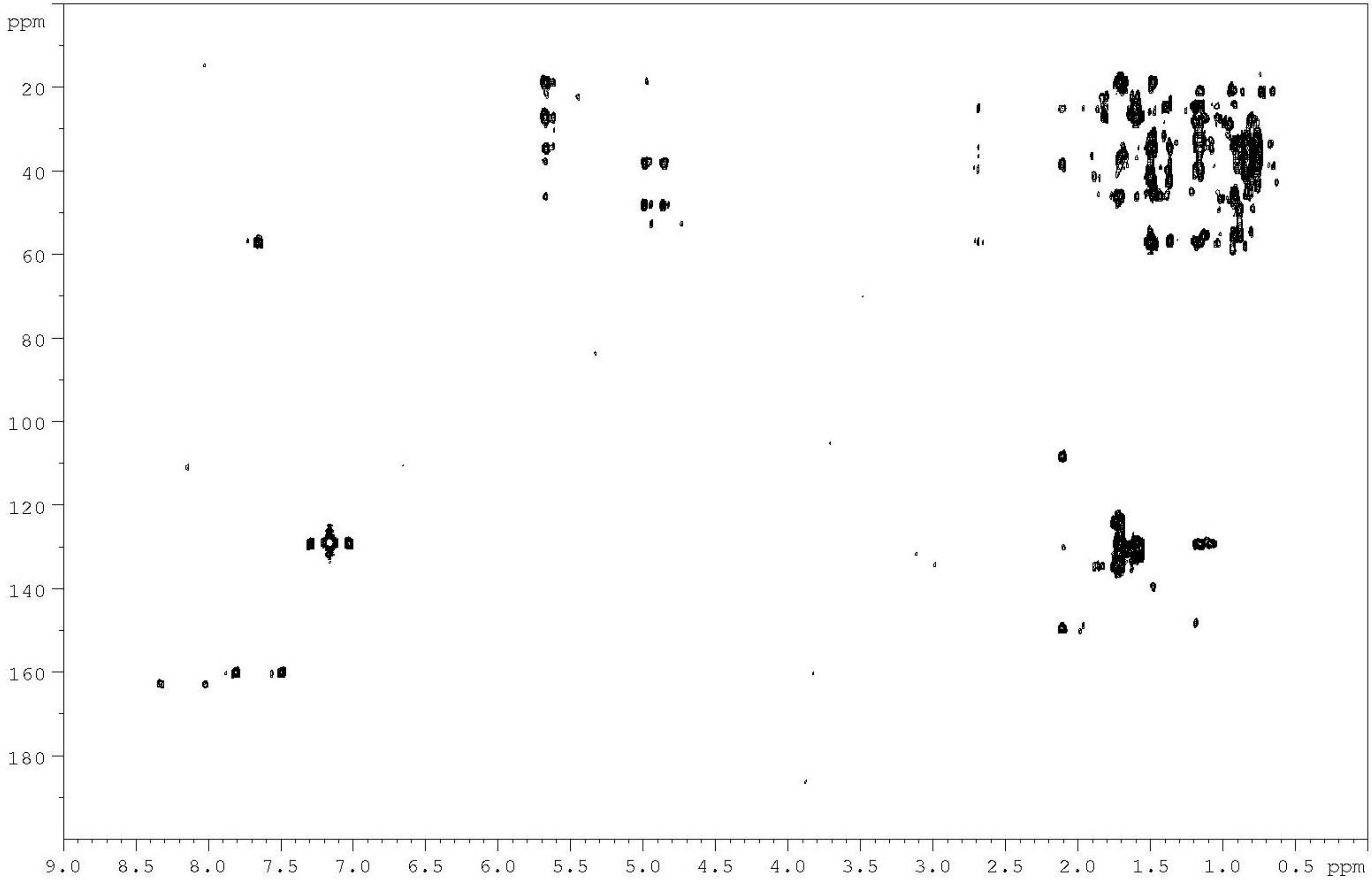

HMBC spectrum of 7-formamido amphilecta-10,14-diene (**9**) (C_6D_6, Bruker 400 MHz, *J*=7Hz).

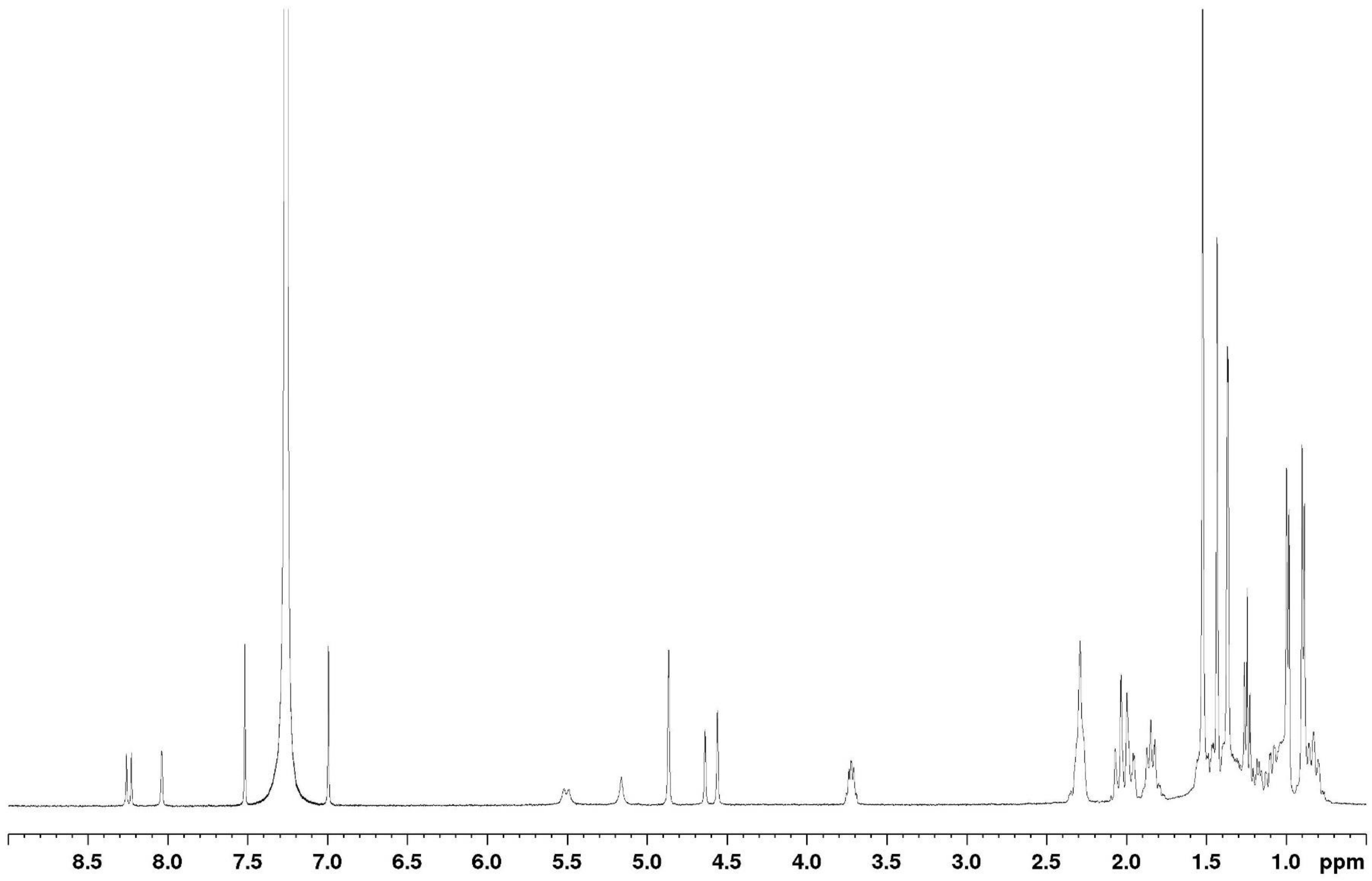

^{1}H NMR spectrum of 8-isocyano-15-formamido-11(20)-amphilectene (**10**) ($CDCl_3$, Bruker 400 MHz).

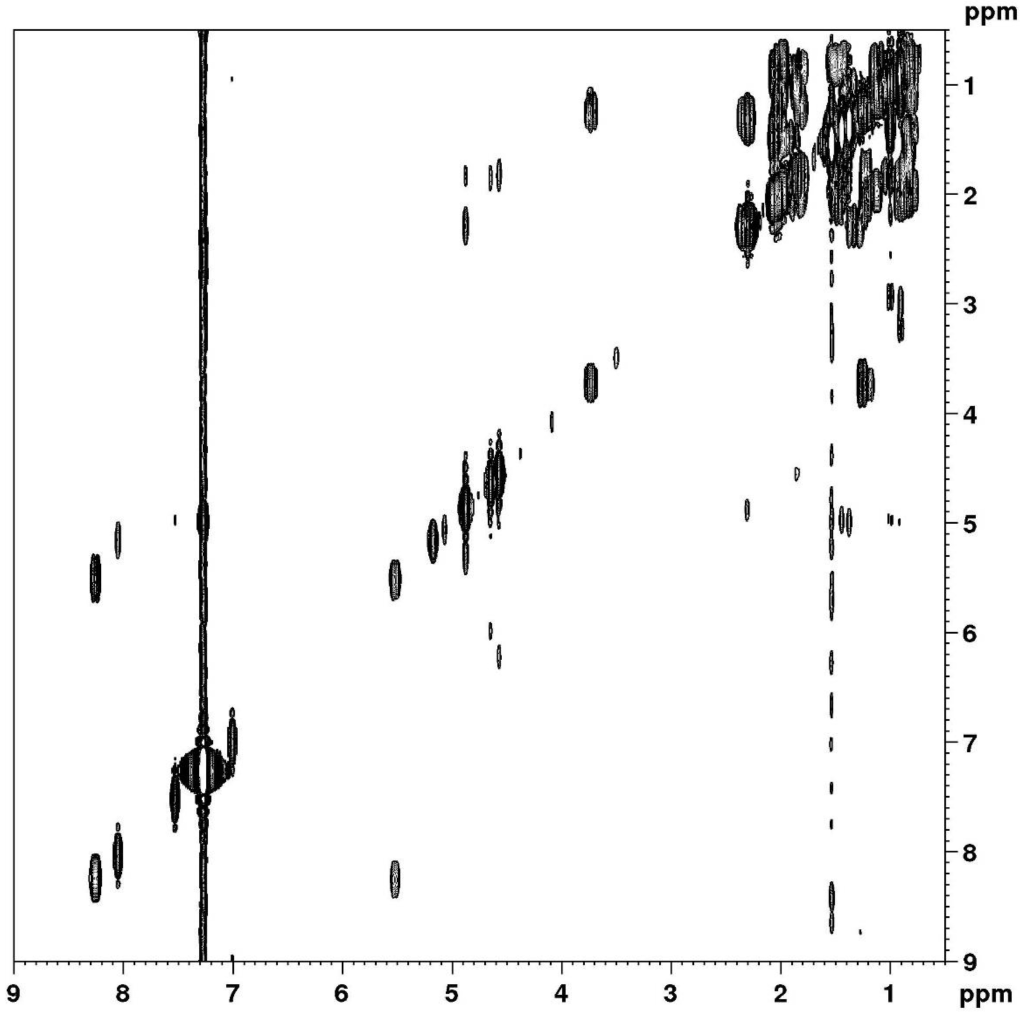

^{1}H-^{1}H COSY spectrum of 8-isocyano-15-formamido-11(20)-amphilectene (**10**) ($CDCl_3$, Bruker 400 MHz).

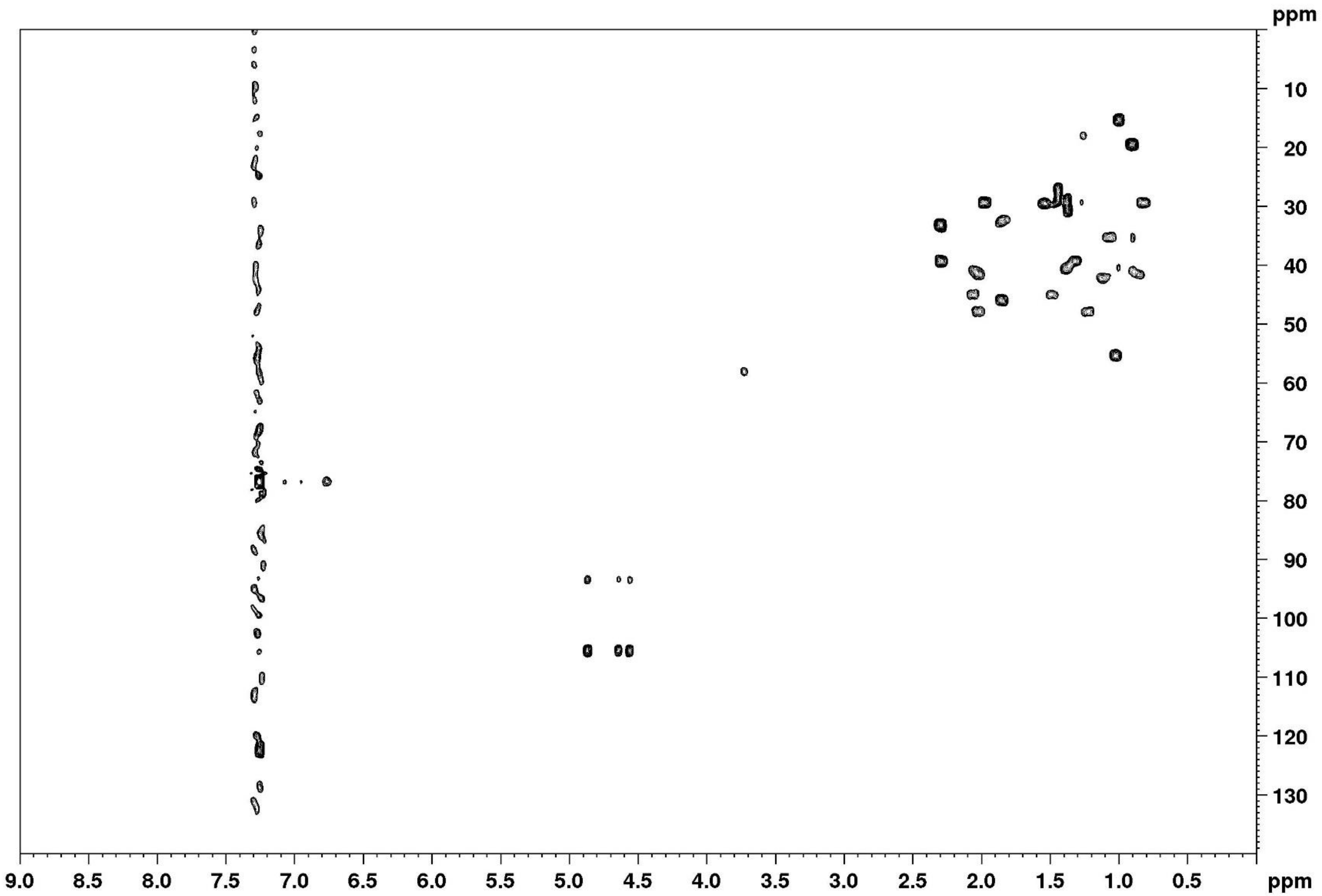

HSQC spectrum of 8-isocyano-15-formamido-11(20)-amphilectene (**10**) ($CDCl_3$, Bruker 400 MHz).

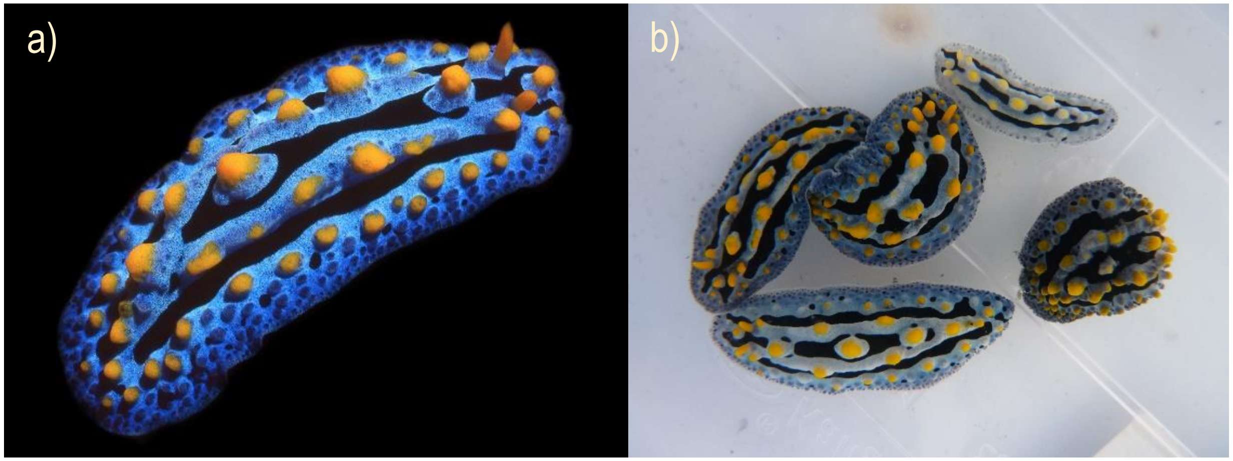

Figure 1. Individuals of *P. coelestis* collected in Hainan (China) in 2002 (a) and 2013 (b).
